# New hemisynthetic derivatives of sphaeropsidin phytotoxins triggering severe endoplasmic reticulum swelling in cancer cells

**DOI:** 10.1038/s41598-024-65335-3

**Published:** 2024-06-25

**Authors:** Aude Ingels, Robert Scott, Annie R. Hooper, Aletta E. van der Westhuyzen, Sachin B. Wagh, Joséphine de Meester, Lucia Maddau, Doris Marko, Georg Aichinger, Walter Berger, Marjorie Vermeersch, David Pérez-Morga, Vladimir A. Maslivetc, Antonio Evidente, Willem A. L. van Otterlo, Alexander Kornienko, Véronique Mathieu

**Affiliations:** 1 Department of Pharmacotherapy and Pharmaceutics, Chemistry and Biochemistry, Faculté de Pharmacie, Université Libre de Bruxelles, Brussels, Belgium; 2 ULB Cancer Research Center, U-CRC, Université Libre de Bruxelles, Brussels, Belgium; 3 Department of Chemistry and Biochemistry, Texas State University, San Marcos, TX 78666 USA; 4 Department of Chemistry and Polymer Science, University of Stellenbosch, Matieland, Stellenbosch, 7600 South Africa; 5 Department of Agriculture, Section of Plant Pathology and Entomology, University of Sassari, Sassari, Italy; 6 Department of Food Chemistry and Toxicology, Faculty of Chemistry, University of Vienna, Vienna, Austria; 7 Medical University of Vienna Center for Cancer Research, Vienna, Austria; 8 Electron Microscopy Laboratory, Center for Microscopy and Molecular Imaging (CMMI), Université Libre de Bruxelles (ULB), Gosselies, Belgium; 9 Institute of Biomolecular Chemistry, National Research Council, Pozzuoli, Italy

**Keywords:** Sphaeropsidin, Diterpene, Iso-pimarane, Endoplasmic reticulum swelling, ER stress, Natural product, Cancer, Cancer, Drug discovery

## Abstract

Sphaeropsidins are *iso*-pimarane diterpenes produced by phytopathogenic fungi that display promising anticancer activities. Sphaeropsidin A, in particular, has been shown to counteract regulatory volume increase, a process used by cancer cells to avoid apoptosis. This study reports the hemi-synthesis of new lipophilic derivatives obtained by modifications of the C15,C16-alkene moiety. Several of these compounds triggered severe ER swelling associated with strong proteasomal inhibition and consequently cell death, a feature that was not observed with respect to mode of action of the natural product. Significantly, an analysis from the National Cancer Institute sixty cell line testing did not reveal any correlations between the most potent derivative and any other compound in the database, except at high concentrations (LC_50_). This study led to the discovery of a new set of sphaeropsidin derivatives that may be exploited as potential anti-cancer agents, notably due to their maintained activity towards multidrug resistant models.

## Introduction

Changes in expression of ion channels and transporters play an integral part in cancer and represent promising targets for therapeutic intervention^1,2^. Indeed, 90% of human tumor samples are found to contain ion channel or transporter mutations^3^. These are often important mechanisms responsible for the development of cancer cell resistance to apoptosis^4^. One such mechanism of resistance involves the impairment of the reduction in cell volume, an important hallmark of apoptosis^5^. It precedes the cytochrome C release, caspase-3 activation, DNA laddering or any other detectable characteristic of apoptosis^6,7^. The reduction in cell volume is brought about by the decrease in intracellular ionic strength via the loss of K^+^ and Cl^−^^8^. Cells resist the apoptotic volume decrease by activating compensatory mechanisms such as regulatory volume increase (RVI) that aims to restore the volume of shrunken cells^9^.

**Figure 1:**
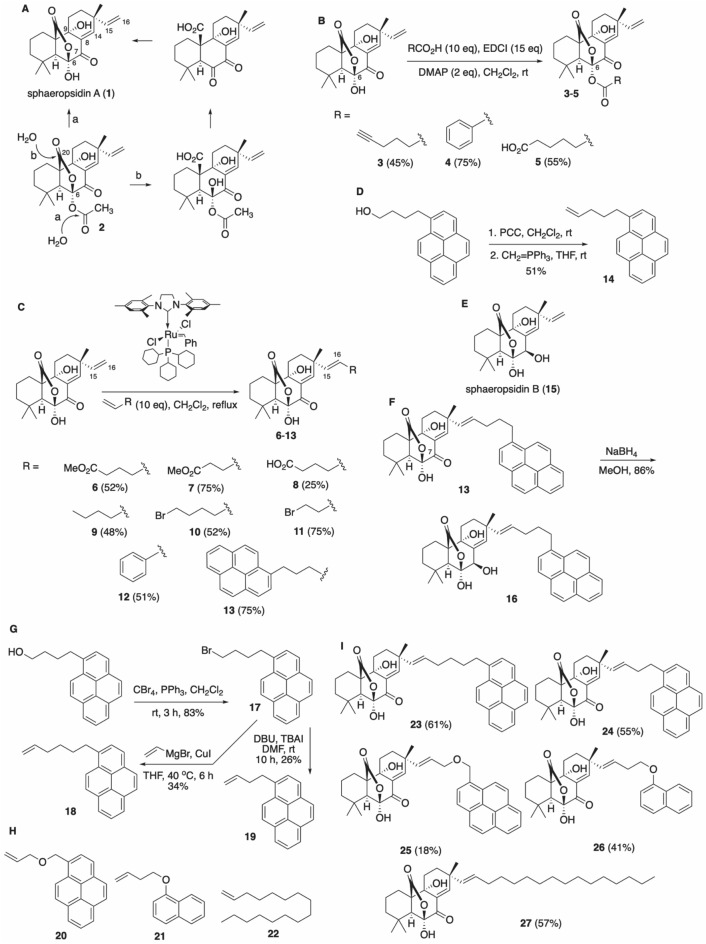
Synthetic sequences involved in the project. (**A**) Two possible hydrolysis pathways for acetate **2**. (**B**) Esterification of **1** at C-6 hydroxyl. (**C**) Cross-metathesis of **1** at C15,C16-alkene. (**D**) Preparation of pyrene alkene **14**. (**E**) Structure of sphaeropsidin B (**15**). (**F**) Synthesis of pyrene-conjugated sphaeropsidin B **16**. (**G**) Preparation of pyrene alkene with a longer linker (**18**) and a shorter linker (**19**). (**H**) Structures of additional alkenes utilized in cross-metathesis reactions with **1**. (**I**) Structures of additional cross-metathesis products synthesized in this work.

We previously discovered that sphaeropsidin A (**1**, Fig. 1A), which is the main phytotoxin produced by *Diplodia* cupressi^10^ showing several interesting biological activities^11^, overcomes apoptosis resistance by inducing a marked, rapid (within 6 h) and sustained cellular shrinkage^12^. This phenotypic change leads to apoptotic cell death 24–48 h later according to TUNEL staining^12^. The lack of close correlation of the differential cellular sensitivities with the 60 cancer cell line NCI panel containing > 763,000 compounds suggested a possibly novel mechanism of action. Sphaeropsidin A inhibits NKCC activity at low micromolar concentration, similar to its GI_50_ ranging from 1.6 to 6.3 µM (except for leukemia cell lines), but other cellular systems including anion exchangers have also been suggested as targets^12^.

To identify compounds more potent than sphaeropsidin A itself we set out to prepare derivatives containing structural changes at sites that were previously shown to retain activity. For example, work in our laboratories indicated that the C7-ketone, C9-OH and C8,C14-alkene functionalities are critical for activity, whereas the C6-OH and C15,C16-alkene can tolerate modifications (Fig. 1A)^13,14^. It should also be noted that previous research has demonstrated that the C6-OH of sphaeropsidin A is readily modified, be it through simple acetylation^13,15–17^ or esterification^14^ whilst structural extension of sphaeropsidin A’s terminal alkene via cross-metathesis has been a fairly recent disclosure^14^ (for a recent review including the structural extension and modification of the sphaeropsidin A skeleton see^18^).

The present report describes the preparation of seventeen new derivatives of sphaeropsidin A containing modifications in the tolerant sites and evaluation of these compounds as potential anti-cancer agents. The results show that we not only were able to improve the antiproliferative potency compared to the natural product itself, but also that several modifications actually led to interesting, and potentiality novel cellular effects related to severe endoplasmic reticulum swelling.

## Results

### Hemi-synthesis of sphaeropsidin A derivatives and their in vitro IC_50_ determination

We first intended to investigate whether the C6-hydroxyl functional group could be esterified with retention of activity, in light of our previous finding that the potency of C6-acetate **2** (Fig. 1A) was equal to that of sphaeropsidin A^13^. It could be hypothesized that acetate **2** readily undergoes intracellular hydrolysis to regenerate the C6-OH of the natural product. If this hydrolysis occurred through the attack of a water molecule on the carbonyl group of the acetate (path a), then introducing bulkier esters at C6 should prevent hydrolysis with a likely abolishment of the activity. In contrast, the attack at the C20 lactone carbonyl (path b) would result in regeneration of activity due to the departure of the C6-ester. To this end we prepared esters **3**–**5** (Fig. 1B) using optimized conditions employing a 10-fold excess of a selected acid, 15-fold excess of 1-ethyl-3-(3-dimethylaminopropyl)carbodiimide (EDCI) and dimethylaminopyridine (DMAP). Ester **3** was aliphatic, ester **4** aromatic, while ester **5** incorporated a polar carboxyl group. As can be seen in Table 1, all compounds **3**–**5** displayed low micromolar activities that were not very different from those of the natural product itself. The delay in the cellular shrinkage of a minimum of 24 h by esters **3** and **4** versus SphA **1**, as determined by videomicroscopy in SKMEL-28 cells (Fig. S1), prompted us to suggest that these compounds indeed undergo intracellular hydrolysis, probably through path b (Fig. 1A). We deduced this because for path a we would have expected the bulkier groups to slow down the direct hydrolytic reversion back to SphA. Confirmation by stability studies in these physiological conditions should be conducted in the future. Altogether, these results suggest that derivatizing the C6-hydroxyl as an ester can be utilized for a prodrug design, for example to enable tumor targeting.

**Table 1:** IC_50_ determined by MTT assays.

Compound	Resistant to apoptosis			Sensitive to apoptosis			Mean
	A549	SKMEL-28	U373	MCF7	Hs683	B16F10	
1*	1.71 ± 0.26	2.32 ± 0.15	2.13 ± 0.17	2.41 ± 0.35	2.31 ± 0.23	1.55 ± 0.27	2.07 ± 0.15
**2**^a^	2.29 ± 0.13	2.68 ± 0.04	0.95 ± 0.02	NA^b^	3.76 ± 0.04	1.56 ± 0.16	2.25 ± 0.48
**3**	2.4 ± 0.10	2.56 ± 0.09	2.37 ± 0.09	3.72 ± 0.27	2.83 ± 0.13	2.60 ± 0.07	2.75 ± 0.21
**4**	1.76 ± 0.29	2.29 ± 0.17	2.19 ± 0.07	1.95 ± 0.14	2.50 ± 0.12	1.88 ± 0.10	2.10 ± 0.11
**5**	1.92 ± 0.07	1.61 ± 0.11	1.82 ± 0.14	2.09 ± 0.07	1.96 ± 0.18	0.78 ± 0.09	1.70 ± 0.19
**6**	2.66 ± 0.04	2.74 ± 0.13	2.81 ± 0.08	2.48 ± 0.13	3.33 ± 0.10	1.81 ± 0.14	2.64 ± 0.20
**7**	4.5 ± 0.07	5.06 ± 0.58	7.75 ± 0.31	6.88 ± 0.26	3.63 ± 0.15	2.33 ± 0.29	5.03 ± 0.82
**8**	> 100	> 100	> 100	> 100	85.3 ± 5.25	81.33 ± 2.42	> 94.44 ± 3.55
**9**	0.63 ± 0.03	1.15 ± 0.11	1.39 ± 0.15	0.47 ± 0.03	1.11 ± 0.14	0.52 ± 0.02	0.88 ± 0.16
**10**	2.96 ± 0.12	3.07 ± 0.10	5.01 ± 0.18	5.36 ± 0.58	4.35 ± 0.27	2.00 ± 0.12	3.79 ± 0.54
**11**	2.00 ± 0.13	2.77 ± 0.18	2.81 ± 0.08	2.89 ± 0.08	2.99 ± 0.07	2.55 ± 0.13	2.67 ± 0.15
**12**	1.61 ± 0.14	2.29 ± 0.09	3.06 ± 0.07	3.06 ± 0.22	2.72 ± 0.11	2.52 ± 0.21	2.54 ± 0.22
13**	0.38 ± 0.03	0.31 ± 0.01	0.57 ± 0.08	0.30 ± 0.02	0.35 ± 0.02	0.29 ± 0.01	0.37 ± 0.04
**14**	78.79 ± 1.26	> 100	> 100	75.95 ± 1.55	96.12 ± 0.50	73.12 ± 1.32	> 87.33 ± 5.17
**1** + **14**	1.05 ± 0.09	2.67 ± 0.09	1.58 ± 0.26	2.65 ± 0.10	2.99 ± 0.12	2.22 ± 0.20	2.19 ± 0.30
**15**	44.84 ± 0.54	59.30 ± 4.51	37.76 ± 1.50	84.47 ± 8.29	43.11 ± 1.07	41.68 ± 1.49	51.85 ± 7.19
**14** + **15**	37.84 ± 1.35	52.13 ± 1.40	44.92 ± 1.11	29.59 ± 4.69	41.14 ± 2.32	22.99 ± 2.76	38.09 ± 4.29
**16**	6.61 ± 0.08	7.24 ± 0.07	8.88 ± 0.19	5.98 ± 0.19	7.34 ± 0.20	5.11 ± 0.42	6.86 ± 0.53

Mean concentration in µM required to reduce the viability of cells by 50% after a 72 h treatment relative to the control.

^a^Data are from ref. 13.

^b^NA = not available.

Data are expressed as mean ± SEM of the six replicates of one experiment except for **1** (*) for which data represent means ± SEM of 7 independent experiments and for **13** (**) for which data represent means ± SEM of 6 independent experiments.

Next, the derivatization of the C15,C16-alkene was performed using an olefin cross-metathesis (CM) strategy. Figure 1C shows that the reaction was quite tolerant of diverse functionality on the alkene component. We found that the reaction went to completion (based on disappearance of sphaeropsidin A) after 10 equivalents of the alkene were added in three portions during 2 h intervals.

Surprisingly, evaluation of compounds **6**, **7**, **9**, **10**, **11**, **12**, containing non-polar functional groups, gave activities comparable to those of sphaeropsidin A. These results suggested that this part of the molecule could be modified for drug optimization during pre-clinical development. Of interest, compound **8** containing a polar carboxyl group was found to be inactive. To test the limit of the size of a non-polar substituent obtained through the cross-metathesis process, we appended a pyrene moiety to obtain conjugate **13** (additionally, it was considered that the fluorescent properties of this fragment might be useful for biodistribution studies as presently done in Fig. 4). The pyrene cross-metathesis precursor **14** was prepared from commercially available 1-pyrenebutanol as illustrated in Fig. 1D. Interestingly, evaluation of compound **13** in our cancer cell panel revealed a significant increase in potency with an IC_50_ 5–10 times lower than compound **1** (Table 1; p = 0.0039 by Mann Whitney comparison). Compound **14** was determined to be inactive when utilized alone and its efficacy in combination with **1** (as a 1:1 mixture) was equivalent to **1** alone (not significant (NS: p > 0.05 by Mann Whitney comparison)). This suggested that **13** does not engage two separate targets in cancer cells, i.e. one target due to the sphaeropsidin A component (**1**) and another due to the pyrene portion (**14**). Furthermore, the activity of sphaeropsidin B (**15**, Fig. 1E), which is markedly lower than that of sphaeropsidin A, was also significantly improved upon its conjugation with the pyrene moiety (compound **16,** Fig. 1F; p = 0.0039 by Mann Whitney comparison), while again, the combined compound treatment with **14** and **15** was not significantly better than **15** alone (Table 1; NS). Clearly, pyrene conjugates **13** and **16** therefore engage new targets in cancer cells that are different from those affected by the natural products or pyrene element separately.

### The pyrene-substituted sphaeropsidin A derivative 13 induces cellular anti-*cancer* effects that differ from those of sphaeropsidin A and are related to severe ER swelling

**Figure 2:**
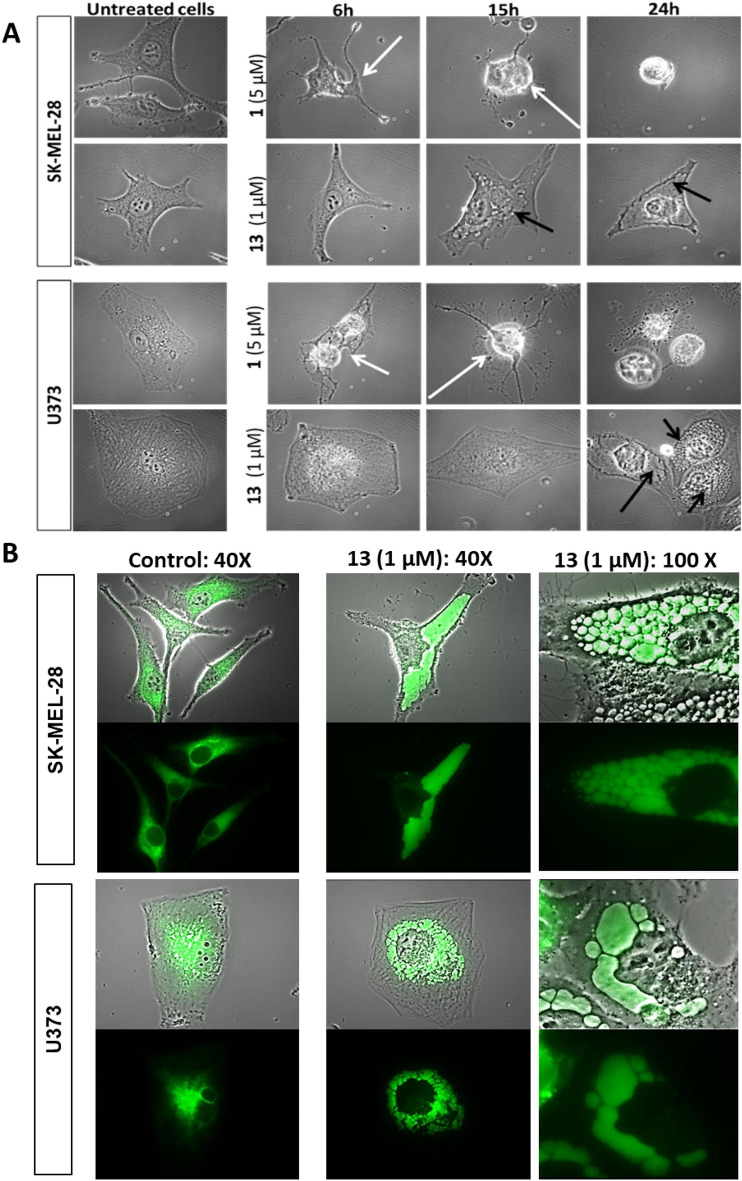
Microscopic study of the effects of **1** and derivative **13** on SK-MEL-28 human melanoma and U373 glioblastoma cells. (**A**) Representative pictures of brightfield morphological microscopic study of the effects. Black arrows: cytoplasmic vacuoles; white arrows: cell shrinkage. (**B**) Derivative **13** induces severe ER swelling in SK-MEL-28 human melanoma and U373 glioblastoma cells. Representative pictures of the staining of cells with the Cell Light® system. Upper pictures of each panel represent merged brightfield and fluorescent images while lower pictures of each panel are fluorescent pictures alone. Green fluorescence results from the transfection of fusion protein (GFP-KDEL-calreticulin based expression assay).

**Figure 3:**
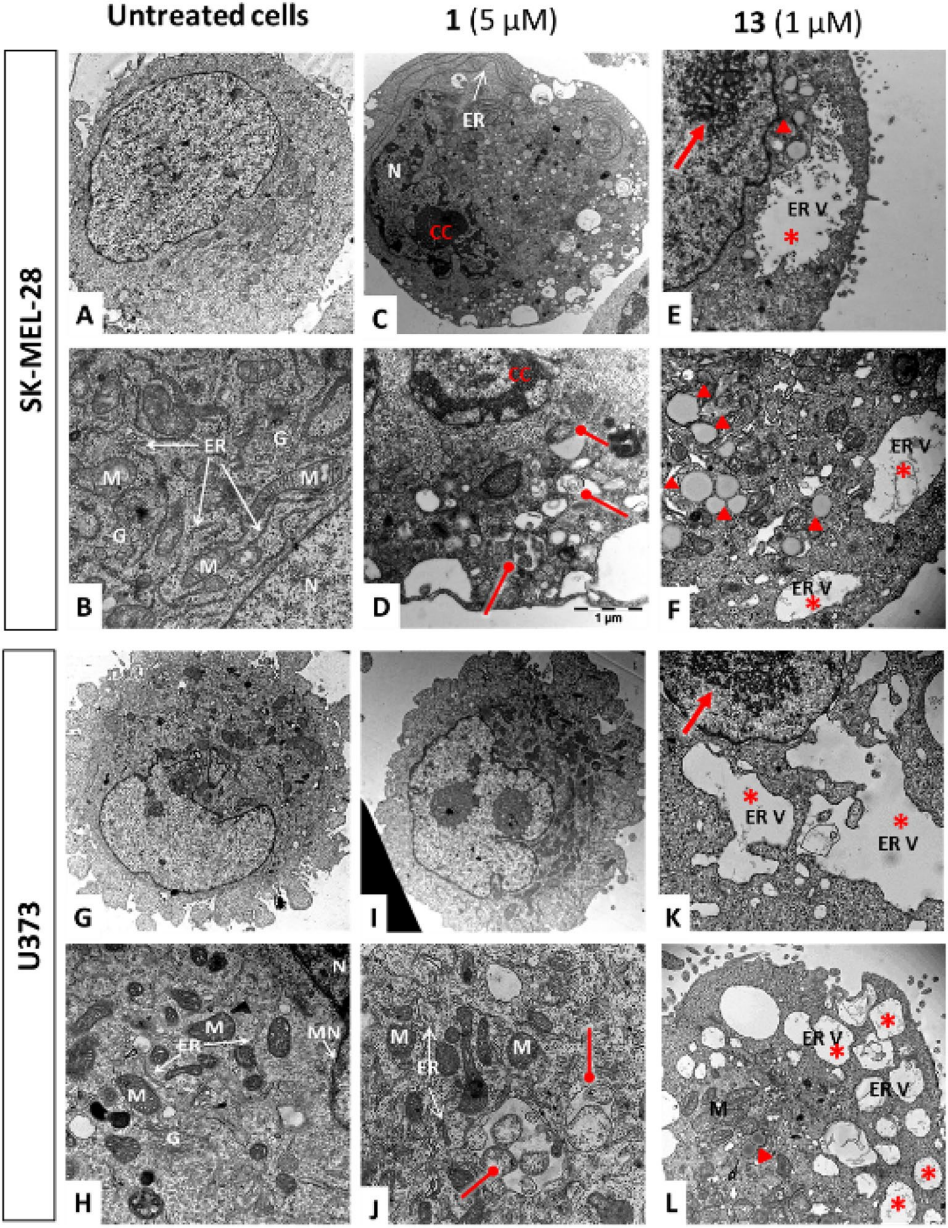
Ultrastructural changes induced by compounds **1** and **13** in SK-MEL-28 human melanoma and U373 glioblastoma cells. ER: endoplasmic reticulum; M: mitochondria; G: golgi apparatus; N: nucleus; MN: nuclear membrane; ER V: ER-derived vacuoles; CC: condensed chromatin; red stars: ER vacuoles; red arrows: Christmas trees, red arrow heads: lipid droplets; bullet-ended arrow: mitophagy.

Morphological comparison of cancer cells treated with **13** and **1** using phase contrast microscopy revealed that compound **13** induces severe vacuolization (black arrows) in cancer cells after 15–24 h depending on the cell model (SKMEL-28 melanoma and U373 glioblastoma cell models respectively), while **1** induces rapid cellular shrinkage (white arrows) within 6 h of treatment in both cell lines (Fig. 2A). Investigation of the vacuole origin in cancers cells treated with **13** was carried out using fluorescent probes for mitochondria, lysosome and endoplasmic reticulum (ER) cell compartments. While the vacuoles were negative to ER tracker®, MitoTracker® or LysoTracker® (data not shown), vacuoles accumulated a fusion protein made of GFP fused to the ER signal sequence of calreticulin and the ER retention signal (KDEL) (Fig. 2B). This fusion protein has been designed to be expressed and retained in the ER; specifically, calreticulin is a multifunctional protein that acts as a major Ca^2+^-binding protein of the lumen of the endoplasmic reticulum. The fusion with the KDEL sequence allows its retention in the ER. Compound **13** appeared thus to induce severe swelling of the ER leading to progressively enlarging vacuoles (Fig. 2B). Upon treatment with **13**, dysfunction of the ER membrane and/or sulfonylurea receptors targeted by the conventional ER tracker® dye could explain the absence of staining with that method. The origin of the vacuoles induced by compound **13** was further confirmed by transmission electron microscopy in both cell models (ER-derived vacuoles (ER V) marked by a red star in Fig. 3).

Although the appearance of the ER membrane is altered, the continuity of the ER V membrane with the nuclear envelope is clearly demonstrated in Fig. 3K. In addition to strong ER structural alterations, we also observed an increase in lipid droplets (red arrow heads) in the cytoplasm and changes in nucleolar ultrastructure characterized by “*Christmas trees*” formation (red arrows; Fig. 3E,K). The latter are composed of pre-rRNA assembled with chromatin^19–21^. A rapid swelling of the ER is associated with an increased demand for membrane and ribosomes that could lead to the features observed, i.e. lipid droplets and nucleolar “*Christmas trees*”. Importantly, these features were not observed with respect to cells treated with compound **1** (Fig. 3C,D,I,J). These cells displayed more affected mitochondrial structures including mitophagy and few ER-derived vacuoles (see red bullet-ended arrows in Fig. 3D,J).

### Compound 13 penetrates into the cell, reaching notably the ER and resulting in strong proteasomal inhibition leading to cell death

**Figure 4:**
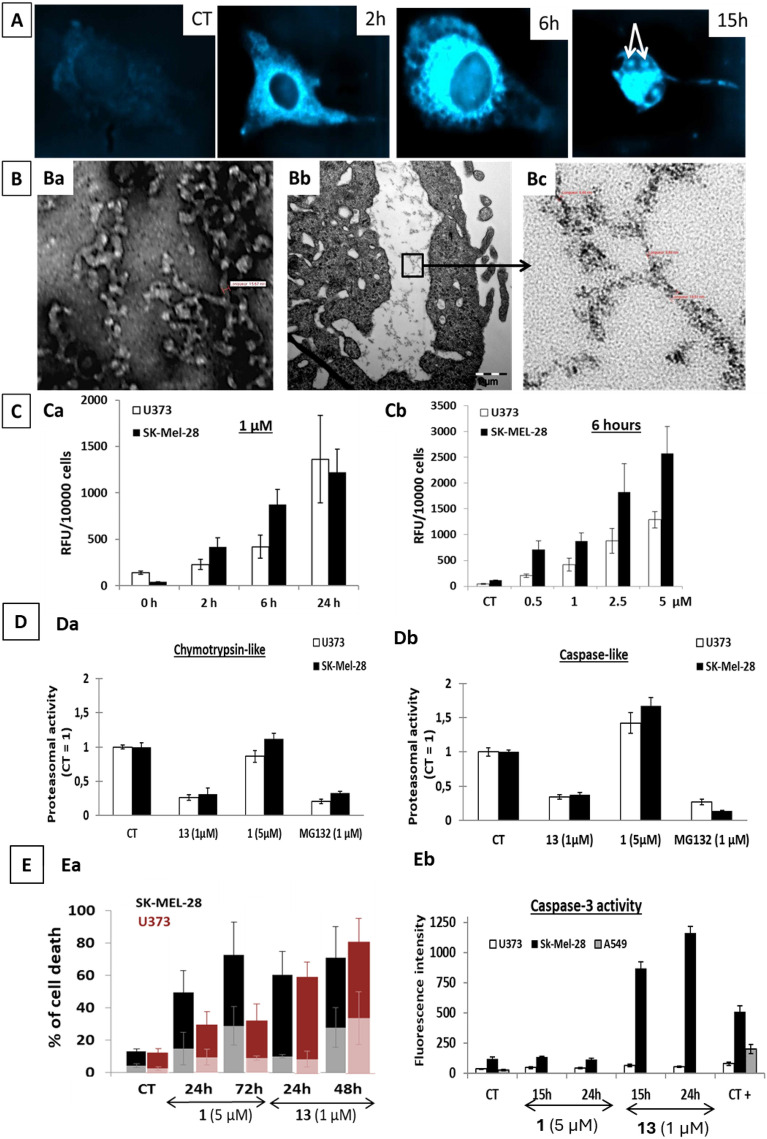
Compound 13 cellular internalization study and its consequences on proteasomal activity and cell death. (**A**) **13** fluorescence observed by fluorescent microscopy in SK-MEL-28 cells over time. Cells were treated with 1 µM of **13** or left untreated (CT). White arrows point nucleoli. (**B**) (**Ba**) TEM image of **13** obtained from dried **13** DMSO stock solution. (**Bb**) TEM illustration of an ER-V-containing materials zoomed in Bc where measurements of the size of the structure were similar (from 8.7 to 19.5 nm) to the one made on picture Ba (15.7 nm). (**C**) quantification of the fluorescence levels of cells treated with **13** at 1 µM over a 24 h period (**Ca**) or with various concentrations of **13** for 6 h (**Cb**). Data are expressed as mean ± SEM of the three replicates of the experiment. (**D**) Proteasomal inhibition induced by **13** treatment. Cells were treated with **13** or **1** for 6 h while treatment with the positive control MG132 lasted for 24 h. Data are expressed as mean ± SEM of three experiments. (**E**) Evaluation of cell death induced by **13** treatment. (**Ea**): Percentage of cells stained by annexin-V alone (A+/PI−: light color bars) and stained by both annexin-V and propidium iodide (A+/PI+; dark color bars) assessed by flow cytometry after treatment with** 1** or **13**. Data are presented as mean ± SEM of three independent experiments conducted each in simplicate. 10,000 events per sample were analyzed. (**Eb**): Caspase 3 activity measurement by fluorescence. For this experiment, A549 non-small cell lung carcinoma cells were used and treated with etoposide (50 µM for 24 h). Data are expressed as mean ± SEM of two independent experiments conducted each in duplicates and triplicates respectively.

Using the fluorescent properties of **13**, microscopic observation suggests ER and nucleoli localization for compound **13** (Fig. 4A), the sites where the ultrastructural modifications were noticed in TEM (Fig. 3). Accordingly, high magnification TEM captions of the ER-vacuoles (Fig. 4Bb,Bc) showed that they contain ultrastructure materials ascribed to compound **13** itself after comparison in terms of size and morphology with TEM images of a dried solution of **13** prepared in DMSO (Fig. 4Ba). Quantitative data revealed that **13** is getting inside the cell in a time- and concentration-dependent manner (Fig. 4C). This compound is incorporated by SKMEL-28 cells quicker than U373 glioma cells, a feature that could explain at least partly why **13**-induced effects are observed earlier and more markedly in those cells (Fig. 2A,B; note that time points for TEM analyses were chosen consistently for each cell line). According to the rapid cell penetration of **13**, effects are irreversible in both cell lines after 6 h of treatment as the compound had already penetrated the cells.

Again, the effects were more pronounced with respect to SKMEL-28 cells (Fig. S2). Noteworthily, we were unable to observe the presence of compound **1** within the cells by TEM, but this is not excluding its intracellular penetration. HPLC dosages are currently envisaged to compare the intracellular penetration of compounds **1** and **13**.

Proteasomal perturbations have been previously shown to lead to severe ER-swelling^22^. To evaluate this hypothesis, we studied the cellular proteasomal trypsin-like, chymotrypsin- like and caspase-like catalytic activities in both cell lines in the presence of **1** and **13**. Figure 4D shows that **13** inhibits chymotrypsin-like and caspase-like activities respectively by more than 60% after only 6 h of treatment in both models, similarly to the positive control MG-132^23,24^. By contrast, compound **1** appeared to stimulate caspase-like activity (Fig. 4D). However, whether proteasomal inhibition is a causal or consequent event to the ER swelling observed with **13** remains to be determined. Compound **13**-induced effects lead to cell death as assessed by annexin-V/PI staining (Fig. 4Ea). Nonetheless, the cell death pathway triggered, e.g. apoptotic versus paraptotic-like seems to be cell-type dependent when considering caspase-8, -9 and -3 activities data (Fig. 4Eb; Fig. S3).

Indeed, caspase -3 (Fig. 4Eb) and -8 (Fig. S3) activities were markedly increased after 15 h of treatment with **13** in SKMEL-28 cells according to high levels of annexin-V positive stained cells (Fig. 4Ea). By contrast, in U373 cells, no caspase activation could be detected (Fig. 4Eb; Fig. S3), at least until 24 h of treatment, although more than 60% of the cells were already positive to annexin-V (Fig. 4Ea). Notably, phosphatidyl-serine externalization could be observed in paraptosis-like cell death too^25^. On the other hand, compound **1** did not seem to induce strong caspase activation in any of these models, a feature that is compatible with its effects described before on the regulatory volume increase allowing the bypass of conventional apoptotic pathways. Finally, various drug resistant cell models, including the p53 null RKO model, highly ABCB1 overexpressing KB-C-1 cells as well as cell lines with acquired cisplatin and oxaliplatin resistance appeared equally sensitive to **13-**induced in vitro anti-cancer effects according to MTT data (Table S1). These encouraging results and the low vulnerability towards multiple important drug resistance mechanisms prompted us to further consider compound **13** as a new promising anti-cancer agent. However, the role of the pyrene moiety had first to be considered and deciphered.

### The DNA targeting properties of the pyrene moiety are not responsible for the ER swelling induced by compound 13 and analogues

Due to the mechanistic differences observed between compounds** 1** and **13**, as well as the increase in potency conferred by the pyrene substitution, compound **13** was accepted for testing by the NCI. The mean GI_50_ of 0.19 µM against the 60 cancer cell line panel was consistent with our results, which showed a mean IC_50_ 0.3 µM with few variations between the cell lines (Table 1; Fig. S4). By contrast, the leukemia cell lines displayed a mean LC_50_ 40 times higher than the global mean LC_50_ of 1.5 µM. Compound **1** was found to be less active against leukemia cell lines^12^. Nevertheless, of significance was that a comparison of the response profile of compound **13** to > 763,000 compounds of the database with the COMPARE algorithm^26^ did not show any correlation between compound **13** and **1** at both GI_50_ and LC_50_ levels. Moreover, at the GI_50_ level, no other compound from the database correlated with **13** (the highest correlation coefficient was only 0.39) suggesting that **13** displays different anti-proliferative mechanisms when compared to the other database compounds, including **1**. In contrast, the COMPARE analysis revealed seven compounds correlating with **13** with respect to LC_50_ profile with CC > 0.7, namely teroxirone, caracemide, tamoxifen, acodazole, amsacrine, pyrazolo acridine and cytembena (Table S2). Three compounds out of the seven are known for their capability to intercalate into DNA and be associated with topoisomerase inhibition^27–31^.

**Figure 5:**
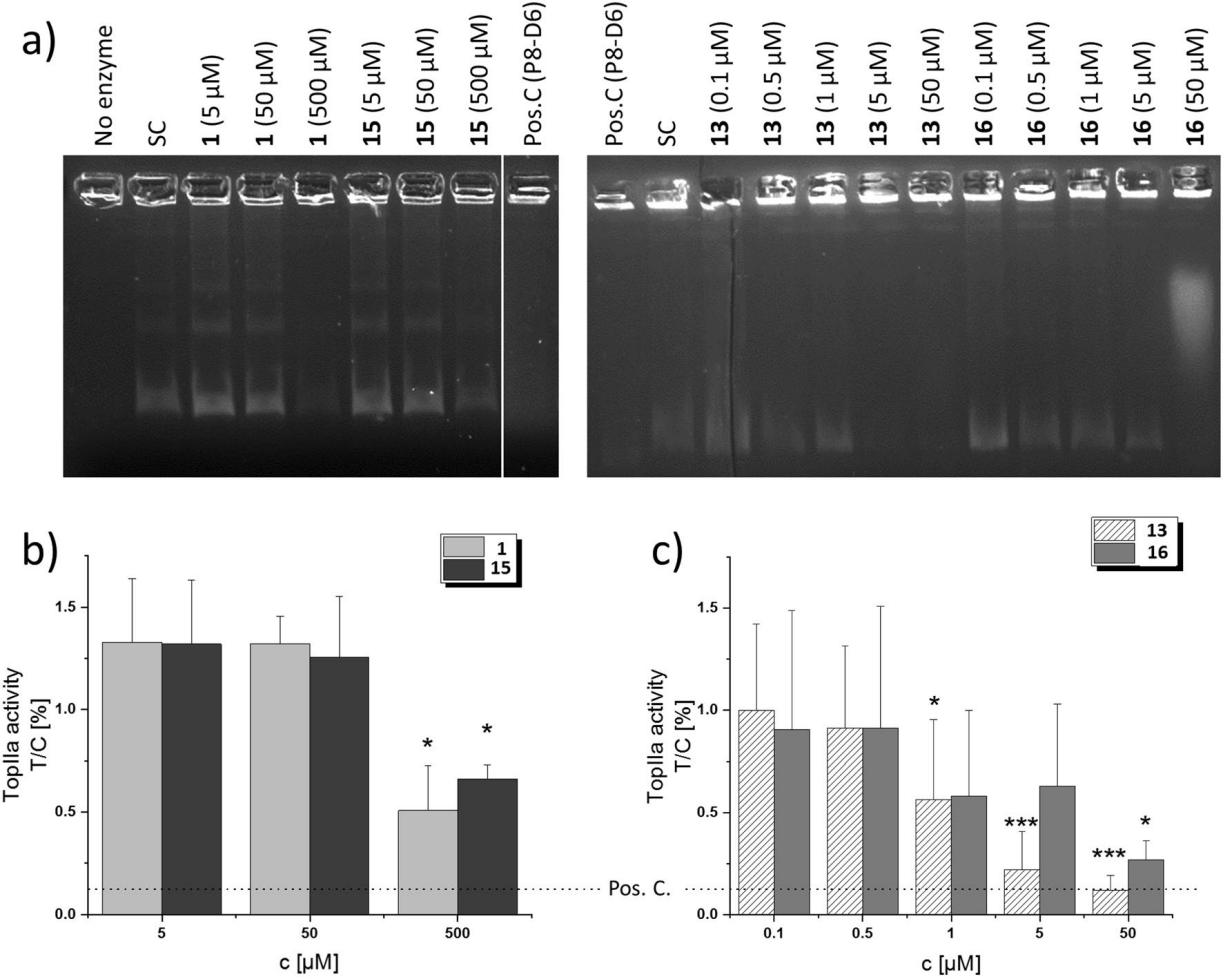
Decatenation Assay of compounds **1**, **15**, **13** and **16**. (**A**) Representative gels show the inhibition of TopIIa by the tested compounds, resulting in the gradual disappearance of decatenated DNA bands as compared to the solvent control (SC). The original uncropped images of the representative gels are supplied in Figs. S5 and Figs. S6. (**B**, **C**) show the relative quantification of decatenated DNA as T/C [%] values, which are expressed as means + SD of at least 3 independent experiments. Significant differences to the solvent control were calculated by one-way ANOVA, followed by Fisher’s LSD post-hoc testing, and are indicated by “*” (p < 0.05), “**” (p < 0.01), or “***” (p < 0.001).

These correlations with DNA interacting agents could thus be conferred by the pyrene moiety. Although this correlation appeared only at the LC_50_ concentration, we investigated such activity in vitro for both compounds **13** and **16** in comparison to their respective parental natural products **1** and **15**. All four compounds inhibited the catalytic activity of human TopIIa in cell-free decatenation assays, albeit they displayed considerable differences in the strength of the effect (Fig. 5). **1** and **15** reduced decatenated DNA levels only at the highest applied concentration of 500 µM (250 and ~ 8 times more than their respective IC_50_ values), at which they led to ~ 50% TopoIIa inhibition (Fig. 5b). Derivatives **13** and **16** both exhibited a concentration-dependent trend of TopIIa inhibition that was observed starting from 1 µM (Fig. 5c). Compound **13** exerted slightly stronger inhibitory properties, being significantly different from the solvent control at concentrations ≥ 1 µM, whereas **15** significantly inhibited the enzyme at 50 µM, although fluorescence images might also indicate a chemical-induced fragmentation of the incubated kDNA (Fig. 5a). These results were obtained in cell-free assays; the intracellular concentrations are probably less than those at which we observed 50% TopoIIa inhibition.

To get more insight into the contribution of the pyrene moiety to the in vitro anti-cancer effects of **13**, we prepared a second small set of derivatized compounds focused on differences involving the pyrene unit. Using our olefin CM process, we synthesized compounds incorporating a longer (**23**, Fig. 1I) and shorter (**24**, Fig. 1I) linkers, oxygen in the linker (**25**, Fig. 1I), a smaller aromatic naphthalene group (**26**, Fig. 1I), and a linear long-chain aliphatic substituent (**27**, Fig. 1I). Figure 1G illustrates the preparation of the requisite alkenes **18** and **19**, while Fig. 1H shows the structures of **20** and **21** prepared using literature methods. In addition, alkene **22** was commercially available.

**Figure 6:**
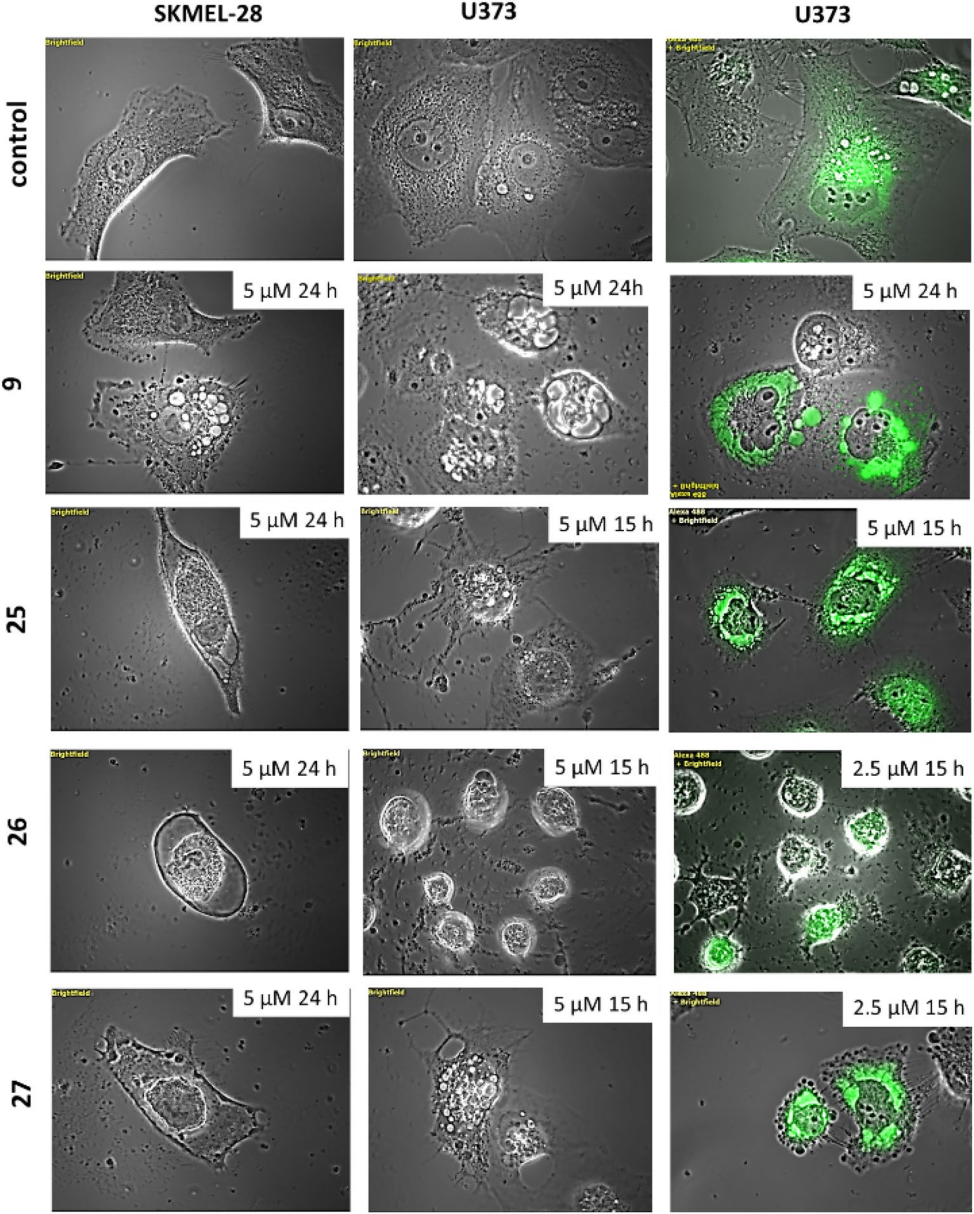
Compound **9**, **25**, **26** and **27** activity –induced vacuolization and other morphological effects in SK-Mel-28 human melanoma cells and U373 human glioblastoma cells. Representative pictures of brightfield images of cells treated with each compound as indicated on the picture itself. Right panel illustrates the staining of U373 cells stained with the Cell Light® system (merged brightfield and fluorescent pictures shown).

Although compounds **25**, **26** and **27** did not display increased in vitro anti-proliferative effects in comparison to **1** (Table 2), morphological evaluation of their effects highlighted in all cases the induction of ER-derived vacuolization (Fig. 6). Cell shrinkage was also observed with respect to **25** and **26**, while evidence of altered plasma membrane was observed after treatment with **27** (Fig. 6). This feature may be attributed to the long alkene chain that may disturb the plasma membrane. These three compounds led to cell detachment from their culture support (more efficiently than **9** and in a similar manner to natural product **1**). Considering these results, the ER swelling induced by several sphaeropsidin derivatives likely depends on the presence of a lipophilic substituent linked to C-16. This small set of pyrene-modified compounds nevertheless provides evidence that the pyrene substituent is not required to induce the severe ER swelling and therefore opens up new avenues for sphaeropsidin-based chemical optimization for anti-cancer purposes. Finally, the relevance of the length of the carbon chain linking the pyrene and the sphaeropsidin scaffolds was evaluated with compounds **23** (longer chain) and **24** (shorter chain). According to the results of Table 2, it appears that the chain linker has a minimal length requirement to increase the anti-cancer activity of the parental compound.

**Table 2:** IC50 determined by MTT assays.

Compound	Resistant to apoptosis			Sensitive to apoptosis			Mean
	A549	SKMEL-28	U373	MCF7	Hs683	B16F10	
**23**	0.60 ± 0.02	0.37 ± 0.01	0.66 ± 0.02	0.42 ± 0.03	0.64 ± 0.02	0.27 ± 0.01	0.49 ± 0.07
**24**	1.2 ± 0.2	1.3 ± 0.14	2.2 ± 0.2	1.2 ± 0.2	2.4 ± 0.1	0.098 ± 0.04	1.40 ± 0.34
**25**	3.47 ± 3.28	3.32 ± 0.30	3.93 ± 0.10	2.71 ± 0.14	3.32 ± 0.23	3.28 ± 0.30	3.34 ± 0.16
**26**	2.05 ± 0.20	2.46 ± 0.08	2.84 ± 0.12	1.30 ± 0.11	3.10 ± 0.23	1.08 ± 0.14	2.14 ± 0.33
**27**	3.10 ± 0.23	0.96 ± 0.02	3.25 ± 0.10	1.78 ± 0.35	2.59 ± 0.38	0.69 ± 0.02	2.06 ± 0.45

Mean concentration in µM required to reduce the viability of cells by 50% after a 72 h treatment relative to the control. Data are expressed as mean ± SEM of the six replicates of one experiment.

## Discussion

Several reports, including our work, highlighted the interesting anti-cancer effects in vitro of sphaeropsidin A (**1**)^12–14^. In particular, **1** has been shown to display both anti-migratory and cytotoxic effects against melanoma cells^12^. These have been associated partly with ion homeostasis disruption via NKCC1 inhibition and/or the targeting of anion exchangers.^12^ Ion channels and transporters represent targets of growing interest since they appear to be involved in cancer development, chemoresistance and migration^4,31–33^. Accordingly, several inhibitors of ion channels, transporters or pumps are being studied in clinical trials either alone (for example the TRPV6 calcium channel inhibitor SOR-C13)^34^ or in combination with approved chemotherapeutic agents (for example digoxine in combination with trametinib, vemurafenib, cisplatin or decitabine; clinical trials.gov)^35^. Despite the deregulation of their targets’ expression in cancer cells^32^, specific delivery to the tumor may be needed to avoid toxicity^36^. This may be particularly the case with respect to natural product** 1**, knowing the high expression levels of NKCC and ion exchangers in the kidneys^37,38^. In addition, we recently reported on **1**’s instability in physiological medium that also needs to be overcome for in vivo administration^39^.

In this study we thus investigated derivatization on two sites on the sphaeropsidin A (**1**) skeleton, namely the C6-OH and C15,C16-alkene previously shown to be tolerant of modifications without losing in vitro anti-cancer activity^13,14^. In terms of a brief overview of the structure activity relationships (SAR) extractable from the small library of compounds synthesized, the following aspects could be determined. As mentioned previously, the sphaeropsidin A-derived esters (**2**–**6**) all had comparable mean IC_50_ values similar to their parent sphaeropsidin A **1** (IC_50_ = 2.07 ± 0.15 μM), indicating probable hydrolysis of these compounds in situ. When sphaeropsidin A’s C_15_=C_16_ alkene was metathetically extended to a long aliphatic chain (**27**, mean IC_50_ = 2.06 ± 0.45 μM) the activity was retained, while a much shorter butyl extension to give **9** gave the best aliphatic derivative (mean IC_50_ = 0.88 ± 0.16 μM). In terms of further comparison of this set of compounds, terminally substituted aliphatic chains featuring bromo atoms (**10** and **11**) or methyl ester groups (**6** and **7**), all had similar mean activities compared to the parent sphaeropsidin A **1**. Of interest, was that the carboxylic acid derivative of **6**, namely compound **8**, lost its activity completely and had a mean IC_50_ of > 94 μM. Concerning the sphaeropsidin A **1** derivatives generated by metathesis extension with alkenes bearing aromatic functional groups, the styrene-derived compound **12** was only slightly less active than its parent **1**. On the other hand, the synthesis of a sphaeropsidin A **1** derivative with a three methylene alkyl chain tethered to the 1-position on the pyrene scaffold gave the most active compound **13** (mean IC_50_ = 0.37 ± 0.04 μM). Extending this chain by one methylene gave compound **23** with similar activity (mean IC_50_ = 0.49 ± 0.07 μM), whilst decreasing the aliphatic tether by one CH_2_ reversed the activity back to that of the parent compound (**24**, mean IC_50_ = 1.40 ± 0.34 μM). Finally, the introduction of an oxygen atom into the 3 carbon-tethered pyrene (to give **25**) or to a 1-naphthyl group (to afford **26**) resulted in sphaeropsidin A **1** derivatives with activities indistinguishable from the parent compound (mean IC_50_’s of 3.34 ± 0.16 and 2.14 ± 0.33 μM respectively).

The most potent derivative of this study, i.e. compound **13**, induces rapid and severe ER-swelling as assessed through fluorescent GFP-KDEL-calrecticulin expression assay and electron microscopy (Figs. 2B and 3). Whether this swelling results from a proteasomal inhibition leading to an ER stress^40,41^ or that the swelling of the ER is the causal event resulting in an inhibition of the proteasome still remains to be investigated. We indeed observed rapid and strong inhibition of chymotrypsin-like and caspase-like proteasomal activities as early as 6 h after treatment initiation with **13** (Fig. 4D). On the other hand, **1** may target NKCC transporters and induces rapid cell shrinkage associated with ion homeostasis disruption^12^. Hypertonic stress has been previously shown to trigger accumulation of unfolded and oxidized protein in the ER that stimulate degradation mechanisms^42^. Under such conditions, increased autophagy and proteasomal activities were previously described, similarly to what we observed herein with respect to **1** (including mitophagy and capsase-like increased activity shown in Figs. 3 and 4D, respectively)^43,44^. Interestingly, NKCC transporters targeted by **1** have been found in abundance within the ER and the Golgi^41^. Although they might be non-functional at those sites^45^, their presence in the ER may facilitate the accumulation of **13** in that organelle after its rapid cellular penetration as observed in Fig. 4A–C. We hypothesize that the addition of a hydrophobic substituent on C-16 may increase the penetration of the compound into the cell, allowing new intracellular distribution. The addition of a hydrocarbon chain is well known to allow an increased cellular uptake in a length dependent manner^46^. To further compare the cellular bioavailability of **1** versus **13**, but also to other derivatives including for example **25**, **26** and **27**, we needed to develop analytical methods and proposed to use them according to Teuscher et al.^47^.

Additional effects linked to ER stress and/or swelling have been observed in **13**-treated cancer cells; these include an increase in lipid droplet content and the appearance of nucleolar “*Christmas trees*” (Fig. 3). Indeed, the ER stress activates lipogenesis through the three main actors of the unfolded protein response (UPR), namely IRE-1 α, Perk and Atf-6^48,49^. The IRE-1 α/XBP-1 pathway activates phosphatidylcholine synthesis. PERK/eIF2 α pathway stimulates Chop and PPARɣ expression promoting lipogenesis, while Atf-6 stimulates phospholipid synthesis. On the other hand, “*Christmas trees*”, formed of pre-transcripted rRNA assembled to chromatin, reflect over-activation of ribosomal biosynthesis^27,50^. The lipid droplets and “*Christmas trees*” may thus be induced by the ER-swelling to face the need for ER-membrane.

The role of the pyrene substituent in the anti-cancer effects observed was further considered. Unsurprisingly, it could contribute to the intercalating properties of **13** at high concentrations only, according to the NCI COMPARE data and in vitro DNA decatenation assay (Fig. 5). Even derivatives bearing only the alkyl tail, such as **27**, also induced strong ER-derived vacuolization, suggesting that the pyrene moiety is not required to induce the ER-swelling observed. By contrast, the pyrene skeleton seems to result in increased potency of the in vitro anti-cancer effects when comparing **13**’s IC_50_ values to those of **1, 9**, **25** and **26** (Table 2). This hypothesis is also supported by the relevance of the length of the carbon chain separating the pyrene unit from the sphaeropsidin A skeleton and its effect on in vitro potency (comparison between **13**, **23** and **24**; Table 2); when the chain is too short, we observe lower potency. Finally, the chemical linkage of the two scaffolds appeared also mandatory to get increased anti-cancer effects and ER-swelling as co-treatment with **1** and **14** did not show any additive effects (Table 1).

Importantly, **13**-induced effects were shown to trigger cell death in both SK-MEL-28 and U373 cell models with resistance to various pro-apoptotic stimuli^51^. In general, the activation of the unfolded protein response (UPR) triggered by ER-stress induces cell cycle arrest and apoptotic cell death^30^. This may be the case with respect to SK-MEL-28 cells that displayed strong capsase-3 activation upon **13**-treatment (Fig. 4E). By contrast, we observed phosphatidylserine externalization and sub-G1 increase (data not shown) in U373 without caspase activation (Fig. 4E, Fig. S3). In these cells, **13**-induced effects may lead to paraptosis-like cell death according to the ER swelling, the proteasomal inhibition, the externalization of the phosphatidylserines and the absence of caspase activation^52,53^. Cell death pathway induction may actually depend on the cell type according to their biochemical characteristics, including for example the expression levels of proteins involved in the UPR. Grp78 is indeed overexpressed by U373 cells in comparison to melanoma cells^54^. Upon ER-stress, Grp78 chaperone dissociates from IRE-1α to be activated and then contributes to unfolded proteasomal protein decay^55^.

Considering our recent findings that the instability of **1** in physiological media is enhanced in the presence of amino-acids^39^, we also worked on sphaeropsidin B skeleton characterized by the C7-alcohol (instead of **1**’s ketone functionality). Importantly, the analogue of **13** prepared using the sphaeropsidin B skeleton (derivative **16**) turned out to be an order of magnitude more potent than sphaeropsidin B itself, displaying an IC_50_ in the low micromolar range and it induced severe ER-swelling at relevant concentrations. Future efforts include evaluation of sphaeropsidin B derivatization series at C15–C16 alkene, development of formulations based on C6-derivatization involving esterase-sensitive prodrug design as well as developing formulation methods to address solubility and stability issues for future in vivo evaluation.

## Conclusions

We recently discovered that sphaeropsidin A, a fungal phytotoxin isolated from *Diplodia cupressi*, overcomes apoptosis resistance in cancer cells by inducing a marked and rapid cellular shrinkage through the impairment of regulatory volume increase^12^. Cell shrinkage was determined to be the cause, not the consequence of apoptosis, and was related to the rapid loss of intracellular chloride^12^. Our results suggest that the prolonged effect of SphA on chloride concentrations, leading to sustained cellular shrinkage, triggers apoptosis directly, bypassing the classical signaling pathways and overcoming apoptosis resistance^12^.

Here, we describe very unusual chemistry of sphaeropsidin A and hemi-synthesis of seventeen new derivatives involving lipophilic substitutions at the C15,C16-alkene. These compounds were obtained in one-step by an efficient cross-metathesis reaction of the natural product with terminal alkene-containing compounds. Our lead derivative containing a pyrene moiety at this position led to potency enhancement of 5–10 times over that of the natural product itself and altered the biological mode of action of the natural product. These analogues now triggered severe ER swelling associated with strong proteasomal inhibition and consequently cell death, a feature that was not observed with respect to the mode of action of the natural product. Importantly, the profile of responses of the 60 cancer cell lines of the NCI to the lead compound did not match with any profile of the NCI compound collection. This study led to the discovery of a new set of sphaeropsidin A derivatives that may be exploited as potential anti-cancer agents, notably due to their novel cell killing mechanism allowing to overcome cancer cell resistance to chemotherapy as assessed by its effects on multidrug resistant models.

## Materials and methods

### Chemistry

#### General

All reagents, solvents and catalysts were purchased from commercial sources (Acros Organics and Sigma-Aldrich) and used without purification. All reactions were performed in oven-dried flasks open to the atmosphere or under nitrogen as described and monitored by thin layer chromatography (TLC) on TLC precoated (250 µm) silica gel 60 F254 glass-backed plates (EMD Chemicals Inc.). Visualization was accomplished with UV light. Flash column chromatography was performed on silica gel (32–63 µm, 60 Å pore size). ^1^H and ^13^C NMR spectra were recorded on Bruker 300, 400 and 500 MHz spectrometers. Chemical shifts are reported in ppm (d) relative to the TMS internal standard. Abbreviations are as follows: s (singlet), d (doublet), dd (double doublet), t (triplet), dt (double triplet), ddt (doublet of double triplets), q (quartet), m (multiplet). HRMS analyses were performed using Waters Synapt G2 LCMS. Compounds **14**^56^, **20**^57^, **21**^58^ were prepared according to literature methods. Sphaeropsidin A (**1**) was obtained by purification of the organic extract of *Diplodia cupressi* culture filtrates as reported previously^10^.

#### General procedure for Steglich esterification

The desired carboxylic acid (0.28 mmol), DMAP (7.1 mg, 0.058 mmol) and EDCI (67.3 mg, 0.43 mmol) were added to a round-bottom flask, under N_2_ and dissolved in 2.5 mL of CH_2_Cl_2_. The reaction was stirred for 1.5 h and monitored by TLC to observe the activation of the acid. Sphaeropsidin A (**1**) (10 mg, 0.028 mmol) was added in one portion and reaction was stirred overnight to produce the desired analogue (as witnessed by TLC). The solvent was evaporated under reduced pressure after which the crude material was dissolved in EtOAc, washed 3 times with deionized water and brine, and dried over MgSO_4_. The desired analogue was purified by flash chromatography (*n*-hexane/CH_2_Cl_2_, 40:1) or by preparatory TLC (CHCl_3_) to obtain the compounds as described below:

**3** (45%): ^1^H NMR (400 MHz, CDCl_3_) δ 6.53 (d, *J* = 1.8 Hz, 1H), 5.79 (dd, *J* = 17.5, 10.7 Hz, 1H), 5.08 (dd, *J* = 17.7, 0.5 Hz, 1H), 5.05 (dd, *J* = 10.0, 0.7 Hz, 1H), 2.86 (s, 1H), 2.76–2.57 (m, 2H), 2.34 (td, *J* = 6.9, 2.6 Hz, 2H), 2.29–2.16 (m, 2H), 1.99 (t, *J* = 2.6 Hz, 1H), 1.96–1.89 (m, 2H), 1.87–1.54 (m, 6H), 1.39–1.30 (m, 1H), 1.27–1.20 (m, 2H), 1.19 (s, 3H), 1.10 (s, 3H), 1.08 (s, 3H). ^13^C NMR (100 MHz, CDCl_3_) δ 189.6, 174.1, 169.3, 148.5, 144.7, 135.9, 113.1, 105.4, 83.2, 71.7, 69.5, 56.2, 51.0, 40.6, 38.9, 33.1, 32.8, 32.7, 29.7, 26.7, 24.5, 23.5, 22.7, 22.5, 17.9, 17.8. HRMS *m/z* (ESI+) calcd for C_26_H_32_NaO_6_ [M + Na]^+^ 463.2091, found 463.2075.

**4** (75%): ^1^H NMR (400 MHz, CDCl_3_) δ 8.13–8.05 (m, 2H), 7.68–7.58 (m, 1H), 7.54–7.44 (m, 2H), 6.61 (br. s, 1H), 5.80 (dd, *J* = 17.5, 10.6 Hz, 1H), 5.07 (dd, *J* = 17.5, 0.7 Hz, 1H), 5.04 (dd, *J* = 10.6, 0.7 Hz, 1H), 2.63 (s, 1H), 2.25 (d, *J* = 13.5 Hz, 1H), 1.97–1.56 (m, 7H), 1.41–1.28 (m, 3H), 1.25 (s, 3H), 1.17 (s, 3H), 1.11 (s, 3H). HRMS *m/z* (ESI+) calcd for C_27_H_30_NaO_6_ [M + Na]^+^ 473.1935, found 473.1920.

**5** (55%): ^1^H NMR (400 MHz, CDCl_3_) δ 6.54 (d, *J* = 1.7 Hz, 1H), 5.80 (dd, *J* = 17.5, 10.6 Hz, 1H), 5.08 (dd, *J* = 17.2, 0.5 Hz, 1H), 5.06 (dd, *J* = 10.9, 0.5 Hz, 1H), 2.86 (s, 1H), 2.65–2.47 (m, 2H), 2.45–2.39 (m, 2H), 2.22 (br. d, *J* = 13.3 Hz, 1H), 1.94–1.87 (m, 1H), 1.85–1.82 (m, 1H), 1.81–1.75 (m, 5H), 1.75–1.54 (m, 5H), 1.40–1.32 (m, 1H), 1.27–1.21 (m, 1H), 1.19 (s, 3H), 1.10 (s, 3H), 1.09 (s, 3H). ^13^C NMR (100 MHz, CDCl_3_) δ 189.8, 178.1, 174.1, 169.4, 148.5, 144.7, 135.9, 113.1, 105.4, 71.8, 56.2, 53.5, 40.6, 38.9, 34.1, 33.5, 32.8, 32.7, 29.7, 26.7, 24.5, 24.0, 24.0, 22.7, 22.5, 17.9. HRMS (ESI) *m/z* calcd for C_26_H_34_NaO_8_ [M + Na]^+^ 497.2146, found 497.2154.

#### General procedure for cross-metathesis

Sphaeropsidin A (**1**) (10 mg, 0.028 mmol) and Grubbs II catalyst (7.4 mg, 0.008 mmol) were dissolved in CH_2_Cl_2_ in a round-bottom flask, a condenser applied, placed under N_2_ and brought to reflux. The desired alkene (0.28 mmol) was added in three portions over 2 h intervals. The solvent was evaporated under reduced pressure and purified by flash column chromatography (*n*-hexane/Et_2_O, 9:1) or by preparatory TLC (*n*-hexane/EtOAc, 7:3).

**6** (38%): ^1^H NMR (400 MHz, CDCl_3_) δ 6.82 (d, *J* = 1.8 Hz, 1H), 5.52–5.36 (m, 2H), 3.67 (s, 3H), 2.72 (s, 1H), 2.30 (t, *J* = 7.4 Hz, 2H), 2.26–2.16 (m, 1H), 2.13–2.04 (m, 3H), 1.90–1.79 (m, 3H), 1.72 (t, *J* = 7.4 Hz, 2H), 1.68–1.60 (m, 5H), 1.37–1.32 (m, 2H), 1.20 (s, 3H), 1.19 (s, 3H), 1.06 (s, 3H). HRMS (ESI) *m/z* calcd for C_25_H_34_NaO_7_ [M + Na]^+^ 469.2197, found 469.2185.

**7** (52%): ^1^H NMR (400 MHz, CDCl_3_) δ 6.80 (d, *J* = 1.7 Hz, 1H), 5.55–5.42 (m, 2H), 5.19 (s, 1H), 3.67 (s, 3H), 2.71 (s, 1H), 2.44–2.31 (m, 4H), 2.22 (br. d, *J* = 9.2 Hz, 1H), 1.92–1.75 (m, 5H), 1.67–1.54 (m, 3H), 1.42–1.31 (m, 2H), 1.19 (s, 3H), 1.19 (s, 3H), 1.06 (s, 3H). HRMS (ESI) *m/z* calcd for C_24_H_33_O_7_ [M + H]^+^ 433.2221, found 433.2223.

**8** (25%): ^1^H NMR (400 MHz, CDCl_3_) δ 6.79 (d, *J* = 1.7 Hz, 1H), 5.53–5.36 (m, 2H), 5.14 (s, 1H), 2.72 (s, 1H), 2.32 (t, *J* = 6.7 Hz, 2H), 2.25–2.21 (m, 1H), 2.15–2.08 (m, 1H), 1.96–1.74 (m, 4H), 1.67–1.54 (m, 5H), 1.40–1.20 (m, 4H), 1.20 (s, 3H), 1.19 (s, 3H), 1.07 (s, 3H). ^13^C NMR (100 MHz, CDCl_3_) δ 192.6, 174.7, 153.4, 137.2, 132.5, 130.4, 128.7, 103.7, 71.2, 57.2, 51.5, 40.5, 38.7, 33.2, 32.7, 32.4, 31.9, 29.9, 27.0, 25.2, 24.1, 23.0, 22.5, 18.1. HRMS (ESI) *m/z* calcd for C_24_H_33_O_7_ [M + H]^+^ 433.2221, found 433.2223.

**9** (48%): ^1^H NMR (400 MHz, CDCl_3_) δ 6.84 (d, *J* = 1.8 Hz, 1H), 5.54–5.35 (m, 2H), 5.20 (s, 1H), 2.72 (s, 1H), 2.22 (br. d, *J* = 8.0 Hz, 1H), 2.02 (q, *J* = 6.4 Hz, 2H), 1.91–1.77 (m, 3H), 1.69–1.54 (m, 5H), 1.40–1.28 (m, 6H), 1.19 (s, 3H), 1.19 (s, 3H), 1.06 (s, 3H), 0.89 (t, *J* = 7.0 Hz, 3H). ^13^C NMR (100 MHz, CDCl_3_) δ 191.8, 174.7, 153.9, 136.2, 132.5, 129.8, 103.7, 71.2, 57.2, 51.4, 40.5, 38.7, 32.7, 32.5, 32.3, 31.6, 30.3, 27.1, 24.9, 23.0, 22.5, 22.3, 18.1, 14.1. HRMS (ESI) *m/z* calcd for C_24_H_34_NaO_5_ [M + Na]^+^ 425.2298, found 425.2283.

**10** (52%): ^1^H NMR (400 MHz, CDCl_3_) δ 6.83 (d, *J* = 1.7 Hz, 1H), 5.52–5.40 (m, 2H), 5.19 (s, 1H), 3.41 (t, *J* = 6.7 Hz, 2H), 2.71 (s, 1H), 2.26–2.21 (m, 1H), 2.10–2.03 (m, 2H), 1.92–1.79 (m, 4H), 1.68–1.49 (m, 9H), 1.39–1.34 (m, 1H), 1.20 (s, 3H), 1.19 (s, 3H), 1.07 (s, 3H). HRMS (ESI) *m/z* calcd for C_24_H_33_BrNaO_5_ [M + Na]^+^ 503.1404, found 503.1561.

**11** (65%): ^1^H NMR (400 MHz, CDCl_3_) δ 6.83 (d, *J* = 1.8 Hz, 1H), 5.56 (dt, *J* = 15.8, 1.0 Hz, 1H), 5.46 (dt, *J* = 15.8, 6.5 Hz, 1H), 5.19 (s, 1H), 3.65 (s, 1H), 3.39 (t, *J* = 6.9 Hz, 2H), 2.72 (s, 1H), 2.64–2.54 (m, 2H), 2.23 (br. d, *J* = 6.3 Hz, 1H), 1.94–1.78 (m, 4H), 1.71–1.56 (m, 3H), 1.40–1.29 (m, 2H), 1.20 (s, 3H), 1.19 (s, 3H), 1.09 (s, 3H).HRMS (ESI) *m/z* calcd for C_22_H_29_BrNaO_5_ [M + Na]^+^ 475.1091, found 475.1091.

**12** (51%): ^1^H NMR (400 MHz, CDCl_3_) δ 7.41–7.28 (m, 4H), 7.25–7.21 (m, 1H), 6.94 (d, *J* = 1.7 Hz, 1H), 6.41 (d, *J* = 16.3 Hz, 1H), 6.18 (d, *J* = 16.3 Hz, 1H), 5.20 (s, 1H), 3.65 (s, 1H), 2.74 (s, 1H), 2.25 (br. d, *J* = 8.1 Hz, 1H), 2.02–1.87 (m, 4H), 1.86–1.73 (m, 1H), 1.66–1.57 (m, 2H), 1.41–1.32 (m, 2H), 1.21 (s, 3H), 1.20 (s, 3H), 1.20 (s, 3H). HRMS (ESI) *m/z* calcd for C_26_H_30_NaO_5_ [M + Na]^+^ 445.1985, found 445.1981.

**13** (75%): ^1^H NMR (300 MHz, CDCl_3_) δ 8.25 (d, *J* = 9.3 Hz, 1H), 8.20–8.07 (m, 4H), 8.06–7.95 (m, 3H), 7.85 (d, *J* = 7.8 Hz, 1H), 6.82 (d, *J* = 1.6 Hz, 1H), 5.57–5.40 (m, 2H), 5.20 (s, 1H), 3.34 (t, *J* = 7.6 Hz, 2H), 2.70 (s, 1H), 2.24–2.14 (m, 2H), 2.01–1.89 (m, 3H), 1.87–1.70 (m, 3H), 1.65–1.51 (m, 4H), 1.41–1.28 (m, 3H), 1.19 (s, 6H), 1.04 (s, 3H). ^13^C NMR (75 MHz, CDCl_3_) δ 191.7, 174.7, 153.6, 136.9, 136.7, 132.5, 131.6, 131.0, 129.9, 129.2, 128.7, 127.6, 127.4, 127.4, 126.7, 126.0, 125.2, 125.1, 125.0, 124.9, 124.8, 123.5, 103.7, 71.1, 57.1, 51.3, 40.4, 38.7, 33.1, 32.7, 32.6, 32.3, 31.5, 30.2, 27.0, 24.9, 22.9, 22.5, 18.1. HRMS (ESI) *m/z* calcd for C_39_H_39_O_5_ [M − H]^−^ 587.2803, found 587.2816.

**16** (86%): ^1^H NMR (300 MHz, CDCl_3_) δ 8.25 (d, *J* = 9.3 Hz, 1H), 8.19–8.06 (m, 4H), 8.03–7.94 (m, 3H), 7.85 (d, *J* = 7.8 Hz, 1H), 5.83 (s, 1H), 5.46 (br. s, 2H), 4.24 (s, 1H), 3.33 (t, *J* = 7.6 Hz, 2H), 2.55 (s, 1H), 2.23–2.06 (m, 3H), 2.00–1.88 (m, 3H), 1.67–1.44 (m, 5H), 1.34–1.16 (m, 4H), 1.19 (s, 3H), 1.10 (s, 3H), 0.96 (s, 3H), 0.96–0.87 (m, 2H). HRMS (ESI) *m/z* calcd for C_39_H_43_O_5_ [M + H]^+^ 591.3105, found 591.3111.

**23** (61%): ^1^H NMR (500 MHz, CDCl_3_) δ 8.26 (d, *J* = 9.2 Hz, 1H), 8.17–8.09 (m, 4H), 8.05–7.97 (m, 3H), 7.86 (d, *J* = 7.8 Hz, 1H), 6.81 (d, *J* = 1.8 Hz, 1H), 5.48–5.36 (m, 2H), 5.19 (s, 1H), 3.35 (t, 2H), 2.69 (s, 1H), 2.19 (d, *J* = 9.1 Hz, 1H), 2.10 (q, *J* = 6.9 Hz, 2H), 1.90–1.80 (m, 4H), 1.77–1.69 (m, 2H), 1.61–1.55 (m, 8H), 1.19 (3, *3*H), 1.19 (3, *3*H), 1.02 (s, 3H). ^13^C NMR (125 MHz, CDCl_3_) δ 191.8, 174.7, 153.7, 137.1, 136.7, 132.6, 131.6, 131.1, 129.9, 129.4, 128.8, 127.7, 127.5, 127.3, 126.7, 126.0, 125.3, 125.2, 125.0, 125.0, 124.8, 123.6, 103.7, 71.1, 57.1, 51.4, 40.5, 38.7, 33.5, 32.7 (2C), 32.3, 31.3, 30.3, 29.4, 27.1, 24.9, 23.0, 22.5, 18.1. HRMS (ESI) *m/z* calcd for C_40_H_42_NaO_5_ [M + Na]^+^ 625.2924; found 625.2938.

**24** (55%): ^1^H NMR (CDCl_3_, 500 MHz) δ 8.25 (d, *J* = 9.3 Hz, 1H), 8.20–8.08 (m, 4H), 8.06–7.96 (m, 3H), 7.83 (d, *J* = 7.7 Hz, 1H), 6.74 (s, 1H), 5.61–5.57 (m, 1H), 5.35 (d, *J* = 16.4 Hz, 1H), 5.16 (s, 1H), 3.43 (t, *J* = 7.6 Hz, 2H), 2.66 (s, 1H), 2.59 (d, *J* = 7.5 Hz, 2H), 2.19 (d, *J* = 8.3 Hz, 1H), 1.86–1.63 (m, 6H), 1.57 (s, 4H), 1.21 (s, 3H), 1.18 (s, 3H), 0.98 (s, 3H). HRMS (ESI) *m/z* calcd for C_38_H_38_NaO_5_ [M + Na]^+^ 597.2611; found 597.2613.

**25** (18%): H NMR (400 MHz, CDCl_3_) δ 8.37 (d, *J* = 9.2 Hz, 1H), 8.26–8.12 (m, 4H), 8.10–7.96 (m, 4H), 6.81 (d, *J* = 1.7 Hz, 1H), 5.76–5.59 (m, 2H), 5.27–5.02 (m, 3H), 4.14 (d, *J* = 4.6 Hz, 2H), 2.67 (s, 1H), 2.19 (br. d, *J* = 10.3 Hz, 1H), 1.89–1.79 (m, 3H), 1.71–1.55 (m, 5H), 1.33–1.27 (m, 2H), 1.19 (s, 3H), 1.18 (s, 3H), 1.06 (s, 3H). HRMS (ESI) *m/z* calcd for C_38_H_37_O_6_ [M − H]^−^ 589.2596, found 589.2599.

**26** (41%): ^1^H NMR (400 MHz, CDCl_3_) δ 8.29–8.19 (m, 1H), 7.86–7.73 (m, 1H), 7.53–7.33 (m, 4H), 6.86 (d, *J* = 1.7 Hz, 1H), 6.81 (d, *J* = 7.4 Hz, 1H), 5.67–5.64 (m, 2H), 5.20 (s, 1H), 4.18 (t, *J* = 6.4 Hz, 2H), 2.70 (s, 1H), 2.69–2.61 (m, 2H), 2.22 (d, *J* = 5.9 Hz, 1H), 1.97–1.78 (m, 3H), 1.67–1.55 (m, 5H), 1.39–1.27 (m, 2H), 1.19 (s, 6H), 1.09 (s, 3H). HRMS (ESI) m/z calcd for C_32_H_35_O_6_ [M − H]^−^ 515.2439, found 515.2441.

**27** (57%): ^1^H NMR (500 MHz, CDCl_3_) δ 6.84 (d, *J* = 1.8 Hz, 1H), 5.50–5.37 (m, 2H), 5.20 (s, 1H), 2.72 (s, 1H), 2.22 (d, *J* = 9.1 Hz, 1H), 2.05–1.96 (m, 2H), 1.96–1.77 (m, 3H), 1.75–1.52 (m, 6H), 1.26 (s, 25H), 1.19 (s, 3H), 1.19 (s, 3H), 1.06 (s, 3H), 0.88 (t, *J* = 6.9 Hz, 3H). ^13^C NMR (125 MHz, CDCl_3_) δ 191.8, 174.7, 153.9, 136.2, 132.5, 129.9, 103.7, 71.2, 57.2, 51.4, 40.5, 38.7, 32.9, 32.7, 32.3, 32.1, 30.3, 29.9, 29.8, 29.8, 29.8, 29.8, 29.7, 29.6, 29.5, 29.5, 29.3, 27.1, 24.9, 23.0, 22.8, 22.5, 18.1, 14.3. HRMS (ESI) *m/z* calcd for C_34_H_54_NaO_5_ [M + Na]^+^ 565.3863; found 565.3867.

### Synthesis of CM precursor 18

To a solution of 1-pyrenebutanol (200 mg, 0.728 mmol) and CBr_4_ (266 mg, 0.801 mmol) in CH_2_Cl_2_, in a round-bottom flask, PPh_3_ (210 mg, 0.801 mmol) was added at 0 °C. The resulting mixture was allowed to warm to rt, and the reaction progress were monitored by TLC. After the complete consumption of starting alcohol (~ 3 h), the solvent was evaporated under reduced pressure. To the resulting brown residue, *n*-hexane was added while stirring, resulting in a white precipitate which was removed by filtration, and after which the filtrate was concentrated under reduced pressure. Column purification of the corresponding residue on silica gel (EtOAc/hexane (5/95)) afforded bromide **17** (205 mg, 0.607 mmol, 83%). Compound **17** (100 mg, 0.297 mmol) was then dissolved in THF in a round-bottom flask. To this solution copper iodide (62 mg, 0.326 mmol) was added at rt and the reaction was stirred for 30 min. At the same temperature, vinylmagnesium bromide (4.24 mL, 2.97 mmol) was added dropwise over 20 min and then reaction mixture was heated at 40 °C. The reaction progress was monitored by TLC. After complete consumption of the starting bromo compound (6 h), the reaction was quenched with aqueous NH_4_Cl. The reaction mixture was extracted with Et_2_O (3 × 5 mL), the combined organic layers were washed with brine (5 mL), dried with MgSO_4,_ and concentrated under reduced pressure. The crude product was initially purified by chromatographic column using EtOAc/*n*-hexane (2/98) yielding a 1:0.7 mixture of compounds **18** and **19** (59 mg, 0.207 mmol, 70%). This mixture of alkenes was again subjected to a gravity column using silica gel and pentane as an eluent. This column purification was found to be successful in separating the two alkenes.

**18** (34%): ^1^H NMR (CDCl_3_, 500 MHz) δ 8.28 (d, *J* = 9.3 Hz, 1H), 8.19–8.08 (m, 4H), 8.06–7.94 (m, 3H), 7.87 (d, *J* = 7.8 Hz, 1H), 5.83 (ddt, *J* = 16.9, 10.2, 6.7, 6.7 Hz, 1H), 5.06–4.94 (m, 2H), 3.36 (d, *J* = 7.9 Hz, 2H), 2.19–2.13 (m, 2H), 1.89 (d, *J* = 146.4 Hz, 2H), 1.71–1.54 (m, 2H). ^13^C NMR (CDCl_3_, 126 MHz) δ 139.0, 137.2, 131.6, 131.1, 129.9, 128.8, 127.7, 127.4, 127.3, 126.7, 125.9, 125.3, 125.2, 125.0, 124.9, 124.8, 123.6, 114.7, 33.9, 33.6, 31.5 29.2. HRMS (ESI) m/z calcd for C_22_H_21_ [M + H]^+^ 285.1638; found 285.1641.

### Synthesis of cross-metathesis precursor 19

Compound **17** (100 mg, 0.297 mmol) was dissolved in 1:1 ratio of dimethylformamide and toluene in a round-bottom flask. To this solution, DBU (89 µL, 0.59 mmol), tetrabutylammonium iodide (11 mg, 0.030 mmol), were added and the resulting solution was heated at 110 °C for 10 h. The reaction mixture was then quenched with the addition of water and extracted with ether (3 × 5 mL). Combined organic layers were washed with brine (5 mL), dried with MgSO_4,_ and concentrated in vacuo. The crude product was purified by chromatographic column using EtOAc/*n*-hexane (3/97) as an eluent. The corresponding alkene product **19** (20 mg, 0.078 mmol, 26%) was isolated as a colorless liquid.

**19** (26%): ^1^H NMR (CDCl_3_, 500 MHz) δ 8.28 (d, *J* = 9.3 Hz, 1H), 8.18–8.10 (m, 4H), 8.05–7.97 (m, 3H), 7.88 (d, *J* = 7.8 Hz, 1H), 6.19–5.85 (m, 1H), 5.23–4.90 (m, 2H), 3.45 (t, *J* = 7.9 Hz, 2H), 2.66–2.53 (m, 2H); ^13^C NMR (CDCl_3_, 125 MHz) δ 138.3, 136.3, 131.6, 131.1, 130.0, 128.8, 127.7, 127.4, 127.4, 126.8, 126.0, 125.3, 125.2, 125.0, 125.0, 124.9, 123.5, 115.3, 36.0, 33.2. HRMS (ESI) *m/z* calcd for C_20_H_16_Na [M + Na]^+^ 279.1144; found 279.1143.

### Cell biology assays

#### Cell culture

Cancer cell lines were obtained from either the European Collection of Cell Cultures (ECACC; Salisbury, UK) or the American Type Culture Collection (ATCC; Manassas, VA). The two glioma cell lines were the U373 (ECACC code 08061901) and Hs683 (ATCC HTB-138) cell lines. The carcinoma cell lines were the A549 non-small cell carcinoma cell line (ATCC code CCL 185) and the MCF-7 mammary breast carcinoma cell line (ATCC code HTB-22). The melanoma cell lines were the mouse B16F10 cells (ATCC code CRL-6475) and the human SKMEL-28 cells (ATCC code HTB-72). The ovarian cancer cell line A2780 and its cisplatin-resistant subline A2780cis were obtained from Sigma-Aldrich (St. Louis, MO, USA). Colon cancer HCT116 cells were obtained from ATCC (CCL-247) and the oxaliplatin-resistant subline HCT116/OxR established in the W. Berger lab^59^. The HeLa cell derivative KB-3-1 and the colchicine-selected, highly ABCB1-overexpressing subline KB-C-1 were generously donated by Dr. D. W. Shen, Bethesda, MD. RKOp53wt colon cancer cells together with the RKOp53KO (homozygous knock-out) subline^60^ were generously provided by Dr. Vogelstein from John Hopkins University, Baltimore. The correct origin of all cell models was confirmed by STR during performance of the experiments. All cell lines were cultured in RPMI1640 culture medium supplemented with 10% heat-inactivated fetal bovine serum, 2% l-glutamine (0.6 mg/mL), 2% penicillin/streptomycin (200 IU/mL and 200 μg/mL) and 0.1 mg/mL gentamicin.

#### Colorimetric MTT assay

The MTT (3-(4,5-dimethylthiazol-2-yl)-2,5-diphenyl tetrazolium bromide) colorimetric assay has been conducted as described previously^61^. Briefly, cells were seeded 24 h before their treatment with each compound for a period of 72 h. The concentrations tested ranged from 10 nM to 100 µM. Each assay was conducted in sextuplicates. The number of independent experiments is mentioned in the legend of the tables.

### Reversibility assay

The reversibility of the effects induced by compounds 13 was evaluated through qualitative microscopic observation of the cell cultures (morphology and confluence of the cell culture) over time after having been exposed or not to the compound for 1, 3 or 6 h. Negative control corresponds to untreated cells while positive control corresponds to cells treated with **13** continuously over the whole experiment. Briefly, 6 × 10^4^ cells per well were seeded in 6 well plates and incubated for 24 h before their use. Culture medium was then replaced by fresh medium with or without compound 13. After 1, 3 or 6 h of treatment, cells were washed and the culture medium was renewed. Pictures were taken at different time points starting at t = 0 when the treatment was applied to the cells, 6 h, 24 h and 72 h thereafter.

### Phase contrast microscopy

For the morphological evaluation of the effects induced by compounds 1 and 13, 6 × 10^4^ cells were seeded on glass coverslips placed in 6 well plates a minimum 24 h before their treatment. At the end of the incubation period with the treatment, coverslips were rinsed once in PBS buffer and directly mounted on slides to allow visualization of the cell with an Axio.Imager.M2 microscope (G X40 and G X100).

### Fluorescent microscopy

To decipher the origin of vacuoles, fluorescent probes staining different cellular compartments were used: the lysosomal Red DND-99 LysoTracker®, the glibenclamide Red ER tracker®, the green FM MitoTracker® that were all purchased from Lifes Technologies (Invitrogen, Belgium) and used according to manufacturer’s instructions. The CellLight ER-GFP (Thermofisher, Belgium) staining of the ER is based on the transfection of a plasmid coding for a fusion protein made of GFP fused to the ER signal sequence of calreticulin and the KDEL ER retention signal. Briefly, 6 × 10^4^ cells were seeded on glass coverslips in 6 well plates. After a minimum of 24 h, cells were treated or not with the compounds of interest. Note that concentration and duration of the treatment were determined separately for each compound prior to the experiment on the basis of the apparition of marked morphological changes in phase contrast microscopy. Sixteen hours before the end of the experiment, 4 µL/well of the reagent were added to allow transfection and expression of the GFP-fused protein by the cells. Coverslips were then washed twice in PBS and mounted on slides without fixation step to take captions with the Axio.Imager.M2 microscope (Carl Zeiss, Belgium). To evaluate the cellular distribution of **13**, the Axio.Imager.M2 was specifically equipped with an M340L4 excitation source (Thorlabs) and the pseudoconfocal Zeiss ZEN deconvolution system kindly provided for demo by Carl Zeiss (Belgium) to allow direct visualization of the compound **13** itself thanks to its intrinsic fluorescent properties.

### Transmission electron microscopy

For TEM, cells were fixed with glutaraldehyde 2.5% (EM grade, Electron Microscopy Sciences, EMS) at 4 °C overnight, washed tree times in cacodylate buffer (pH 7.4) and postfixed 1 h at room temperature in 1% OsO4, (EMS), 1.5% potassium ferrocyanide (Sigma-Aldrich) in 0.15 M cacodylate buffer. This was immediately followed by a second incubation in 1% OsO4 for 1 h at room temperature. After washing in distilled water, samples were stained with 1% uranyl acetate (EMS) and serially dehydrated in increasing ethanol concentrations. Samples were then embedded in epoxy resin (Agar 100 resin, Agar Scientific Ltd) and left to polymerize for 2 days at 60 °C. Ultrathin sections (50–70 nm thick) were collected with a Leica UC6 ultra-microtome on formvar-carbon-coated copper grids and further stained with uranyl acetate and lead citrate by standard procedures. Observations were made on a Tecnai 10 transmission electron microscope (FEI) and images were captured with a Veleta camera and processed with SIS iTEM software (Olympus).

### Fluorimetric quantification of 13 cellular content

The fluorescent properties of **13** (maximal excitation at 340 nm and emission at 380 nm in PBS buffer with 5% FBS) allowed us to quantify the cellular content of **13** over time after exposure to the compound at various concentrations. Briefly, 5 × 10^3^ cells/well were seeded in 24 well plates 24 h before their treatment with **13** for 2–24 h. At the end of the treatment, cells where washed three times with PBS and further detached with trypsin/EDTA (100 µL per well). 150 µL of PBS with 5% FBS were added to each well to inactivate trypsin/EDTA. 100 µL of the cell suspension were then transferred to black 96 well plates for fluorescent measurement (Ex: 340 nm; Em: 380 nm; SynergyMX Biotek, USA). The fluorescence levels were finally adjusted according to the cell concentration and normalized to 10^4^ cells.

### Catalytic proteasomal activities measurement in cells

The chemotrypsin-like, trypsin-like and caspase-like activities of the proteasome were evaluated in cells with the G8660, G8861 and G8761 kits respectively according to the manufacturer’s instructions (Promega, USA). Briefly, the assays are based on the use of short amino-acid sequences coupled with luciferin and that can be specifically cleaved by each catalytic unit of the proteasomal complex. For this purpose, cells were seeded in 96 well plates 24 h before their treatment with **1** (5 µM), **13** (1 µM) for 6 h or with the positive control MG132 (1 µM) for 24 h. At the end of the treatments, cells were washed three times with PBS and permeabilized with digitonin (0.5 mg/mL; 50 µL per well) under shaking for 10 min. Then, 50 µL of a mixture of the substrate of interest and luciferase were added for 15 min under slow shaking. Luminescence levels quantified with the Active-Glo LR-100 luminometer (DSLabs, Suisse) were normalized according the number of viable cells evaluated in parallel in the same conditions by means of MTT assay.

### Cell death evaluation

Cell death induced by compounds **1** and **13** was evaluated by means of the double annexin-V/propidium iodide staining by flow cytometry with the FITC Annexin V apoptosis detection kit from BD Pharmingen (USA) according to the manufacturer’s instructions. Briefly, cells were cultured and treated in 25 cm^2^ flasks. At the end of the treatment, all cells (adherent and floating cells) were collected, washed and resuspended at 10^6^ cells/mL. 100 µL of the cell suspension were stained with 5 µL of FITC-annexin-V and 5 µL of propidium iodide for 15 min at rt in the dark. 400 µL of buffer were then added before data acquisition of 10,000 events per sample (Gallios, Beckman Coulter, Belgium). Caspase activation is usually evaluated to confirm or not the apoptotic feature of the cell death induced. For this purpose, we used the caspase-3, -8 and 9 multiplex assay (Ab219915, Abcam, UK) following manufacters’ instructions. In this assay, cells were exposed to short peptidic sequences linked to different fluorochromes and that could be specifically cleaved by each of these caspases. This assay thereby allowed simultaneous evaluation of the three caspase activities thanks to the quantification of the three fluorophores released in a SynergyMX plate fluorimeter (Biotek, USA).

### Topoisomerase activity assay

The topoisomerase (Top) activity assay kit, including kinetoplast DNA (kDNA and recombinant human TopIIa enzyme, was obtained from TopoGen (Port Orange, FL, USA), agarose from PeqLab (Erlangen, Germany), 50 × TAE buffer from Roth (Karlsruhe, Germany) and ethidium bromide solution (10 mg/mL) from Sigma-Aldrich (Taufkirchen, Germany). Decatenation assays were performed as described previously^62^ with slight modifications according to the manufacturer’s protocol. 100 µM of the recently described dual topoisomerase poison P8-D6 served as a positive control^63^, and 5% DMSO as a solvent control. Briefly, the compounds were dissolved in DMSO and diluted to 20× the concentration to be tested. 1 µL of the resulting stock solutions were mixed with 18 µL of a buffer containing 200 ng kDNA and 1 mM ATP. Subsequently, 1 µL of a 1u/µL dilution of TopIIa enzyme was added and incubated at 37 °C for 30 min. Afterwards, the reaction was stopped by addition of Stop buffer, and electrophoresis was carried out on a 1% agarose gel in TAE buffer. The gel was stained with 0.5 µg/mL ethidium bromide, washed with water, and fluorescence images were captured with the LAS4000 system (Fujifilm, Duesseldorf, Germany). All steps were carried out on ice.

### Supplementary Information

Supplementary Information.

## Data Availability

The authors declare that the data supporting the findings of this study are available within the paper and its Supplementary Information files. Should any raw data files be needed in another format they are available from the corresponding author upon reasonable request. Source data are provided with this paper.
