# Supplementary material for: New hemisynthetic derivatives of sphaeropsidin phytotoxins triggering severe endoplasmic reticulum swelling in cancer cells
Source: Sci Rep. 2024 Jun 25;14:14674. doi: 10.1038/s41598-024-65335-3 (PMC11199504; doi:10.1038/s41598-024-65335-3)
Supplement: Supplementary file 1 — Supplementary Information. [file 41598_2024_65335_MOESM1_ESM.pdf]

Supplementary Information

New hemisynthetic derivatives of sphaeropsidin phytotoxins triggering severe endoplasmic reticulum swelling in cancer cells

Aude Ingels,[1,2,‡] Robert Scott,[3,‡] Annie R. Hooper,[3,‡], Aletta E. van der Westhuyzen,[4] Sachin B. Wagh,[3] Joséphine de Meester,[4] Lucia Maddau,[5] Doris Marko,[6] Georg Aichinger,[6] Walter Berger,[7] Marjorie Vermeersch,[8] David Pérez-Morga,[8] Vladimir A. Maslivetc,[3] Antonio Evidente,[9] Willem A. L. van Otterlo,[4] Alexander Kornienko,[3,*] and Véronique Mathieu[1,2*]

[1] Department of Pharmacotherapy and Pharmaceutics, Chemistry and Biochemistry, Faculté de Pharmacie, Université Libre de Bruxelles, Brussels, Belgium
[2] ULB Cancer Research Center, U-CRC, Université Libre de Bruxelles, Brussels, Belgium
[3] Department of Chemistry and Biochemistry, Texas State University, San Marcos, Texas 78666, USA
[4] Department of Chemistry and Polymer Science, University of Stellenbosch, Matieland, Stellenbosch, 7600, South Africa
[5] Department of Agriculture, Section of Plant Pathology and Entomology, University of Sassari, Sassari, Italy
[6] Department of Food Chemistry and Toxicology, Faculty of Chemistry, University of Vienna, Austria
[7] Medical University of Vienna Center for Cancer Research, Vienna, Austria
[8] Electron Microscopy Laboratory, Center for Microscopy and Molecular Imaging (CMMI), Université Libre de Bruxelles (ULB), Gosselies, Belgium
[9] Institute of Biomolecular Chemistry, National Research Council, Pozzuoli, Italy

‡ Co-first authors

* Corresponding authors

SI Figure 1: time-lapse cellular morphological effects of **1**, **3** and **4** on SKMEL-28 melanoma cells.

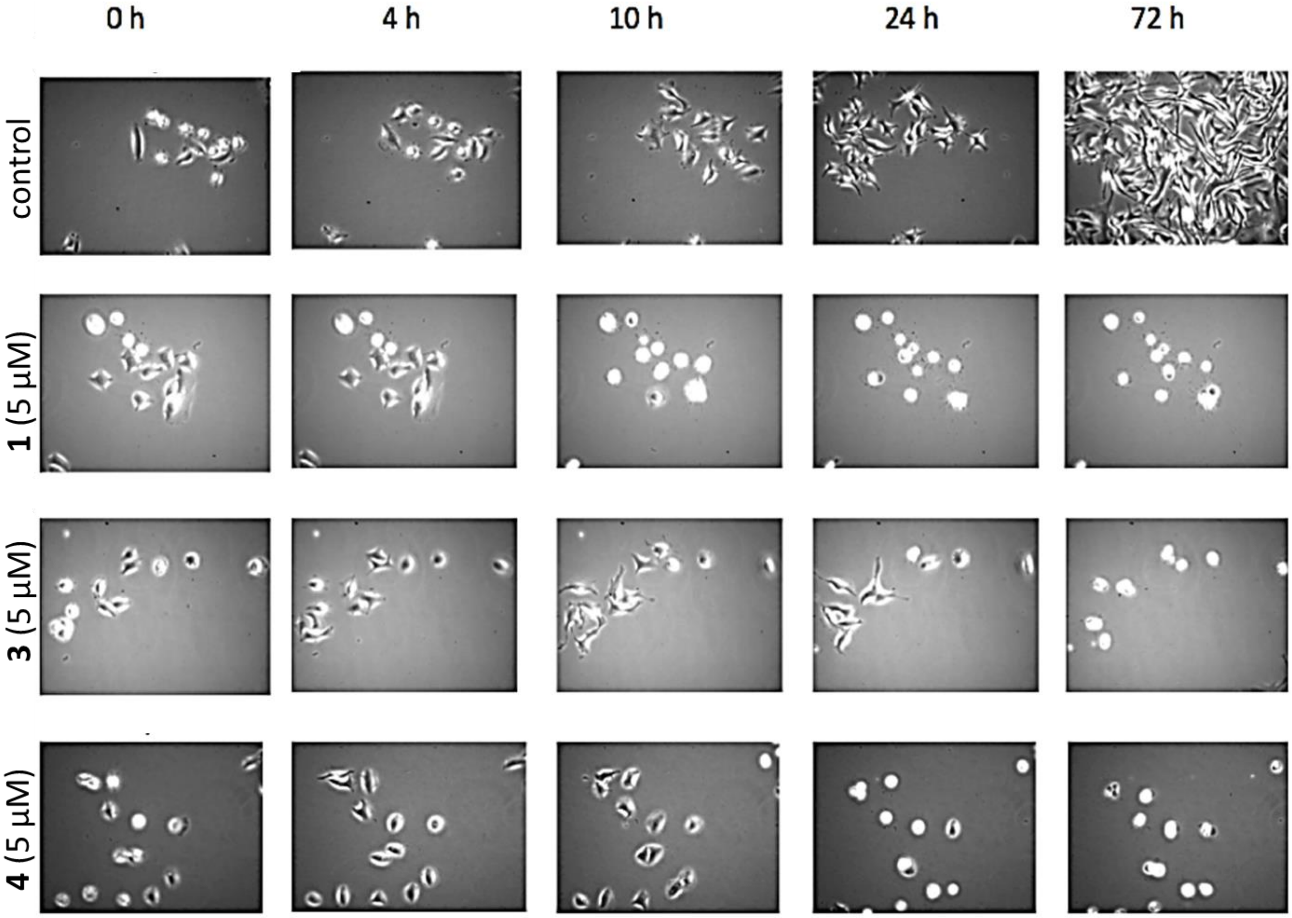

SI Figure 2: reversibility assay. Morphological micrographs of SKMEL-28 and U373 cells treated or not with **13** for indicated periods of time before refresh with new culture medium till the end of the experiment.

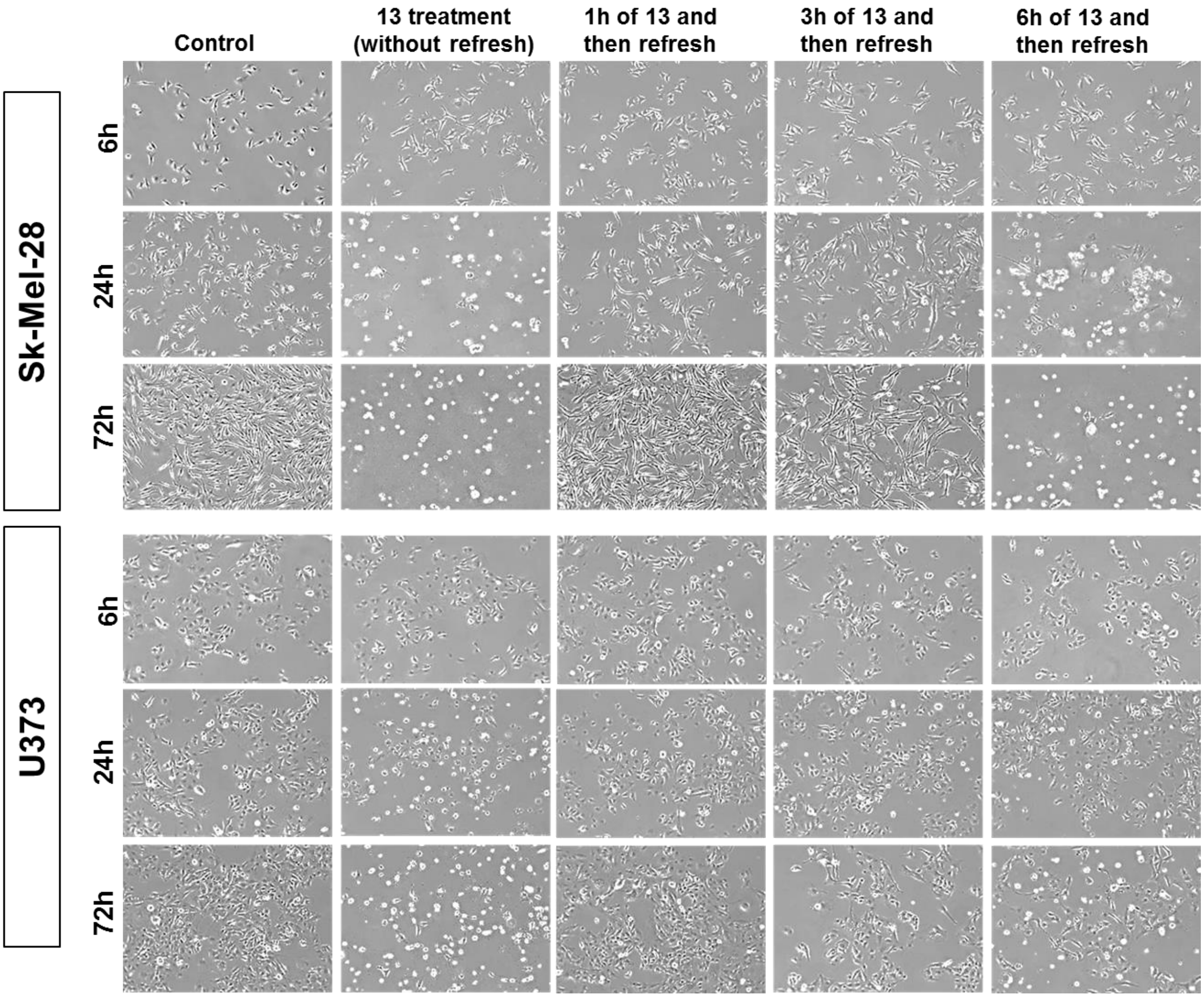

SI Figure 3: caspase-8 and caspase-9 activities as measured by the fluorescence levels of their cleaved substrates. Data are expressed as mean +/- SEM of two independent experiments conducted each in duplicates and triplicates respectively.

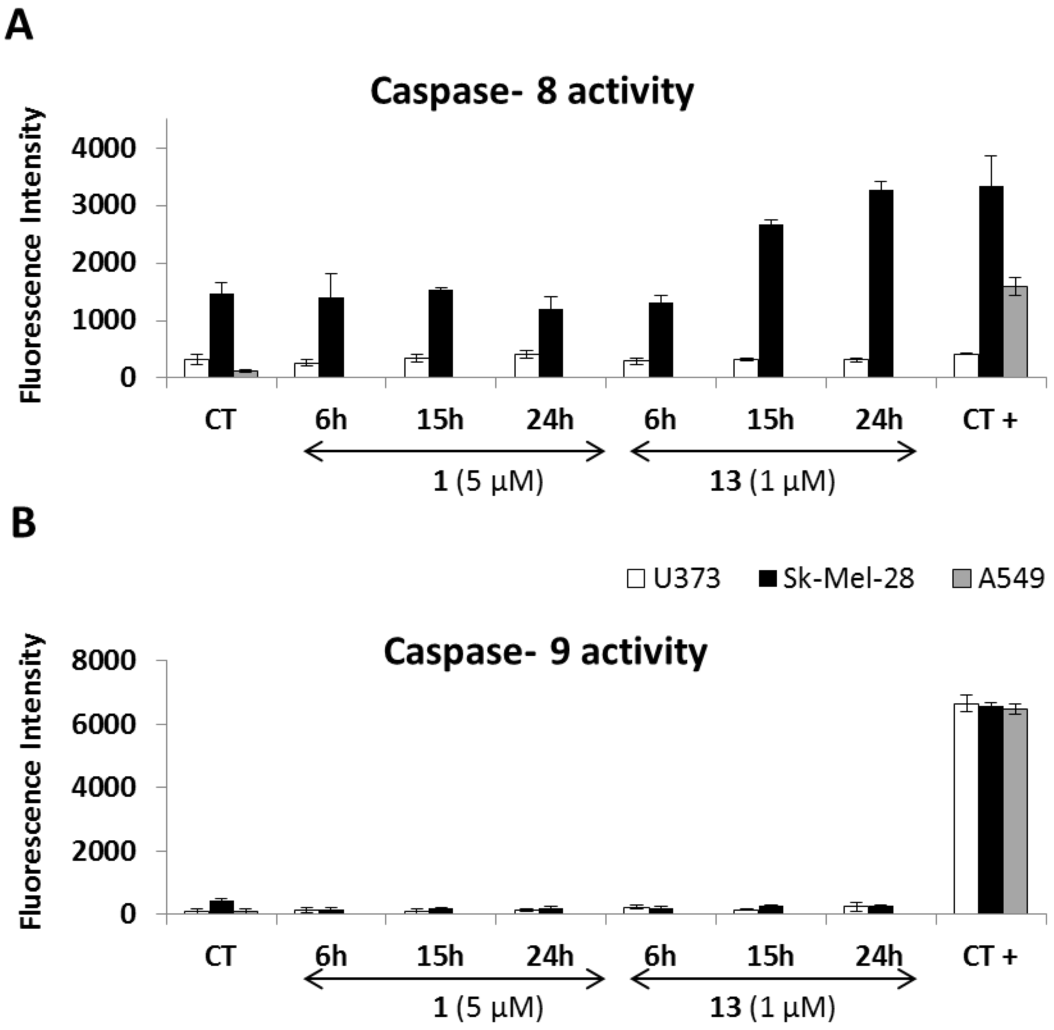

SI Figure 4: NCI analyses of the in vitro anti-cancer activity of 13 and 1 on the 60 cancer cell line panel. Cell lines are grouped by cancer type [origin]. Determination of the concentration that reduced by 50 % the global growth [GI_{50}] of each cell line after 48 h of culture. ‘‘0’’ represents the mean GI_{50} of the 60 cell lines. Log10 differences are represented by the bars. If a cell line is more sensitive, the bar is on the right, if it is less sensitive, the bar is on the left. Similar data are represented for the 50 % lethal concentration [LC_{50}] of each cell line. Both data for compounds **13** and **1** are provided.

13

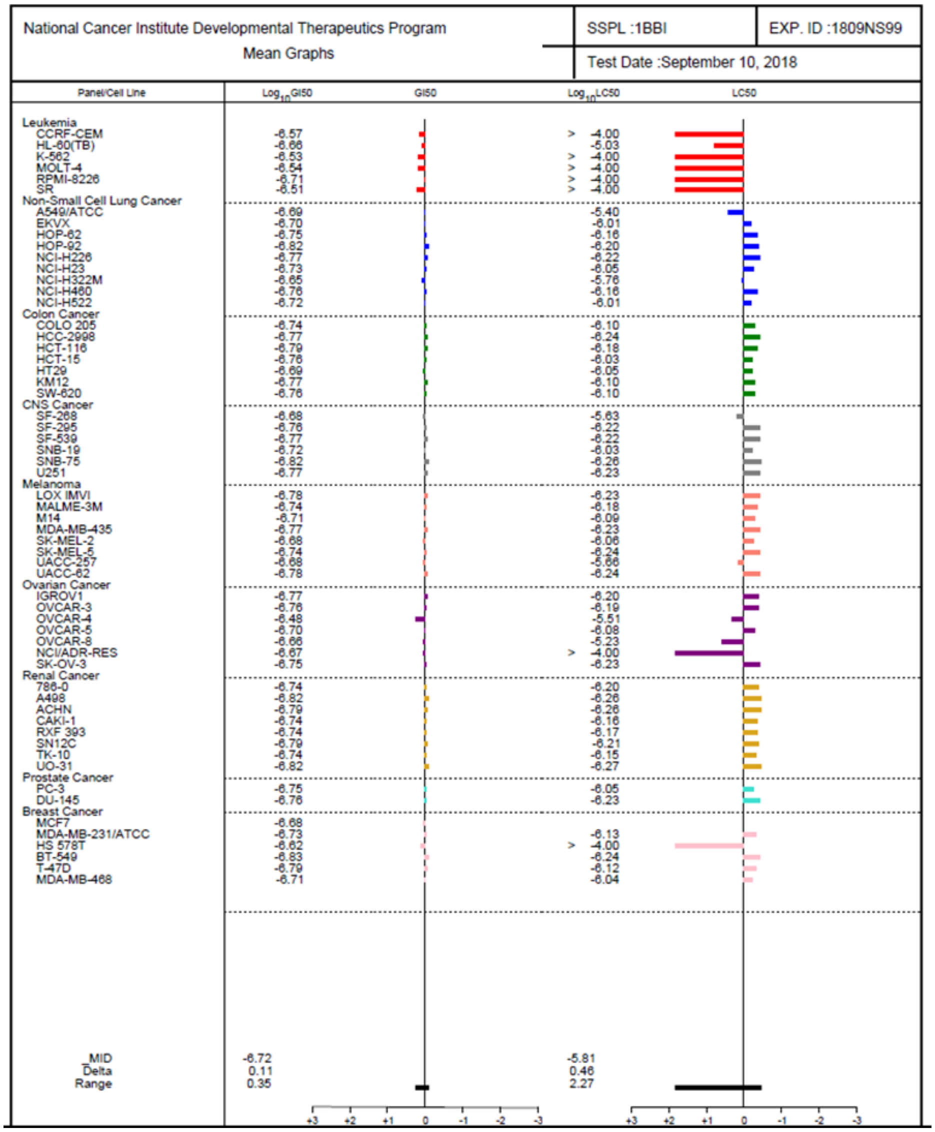

1

National Cancer Institute Developmental Therapeutics Program Mean Graphs	SSPL :0YBN	EXP. ID :1208NS28
	Test Date :August 13, 2012	

Panel/Cell Line	Log_{10}GI50	Log_{10}LC50
**Leukemia**		
CCRF-CEM	-6.42	> -4.00
HL-60(TB)	-5.91	> -4.00
K-562	-5.67	> -4.00
MOLT-4	-5.76	> -4.00
RPMI-8226	-5.95	> -4.00
SR	-5.99	> -4.00
**Non-Small Cell Lung Cancer**		
A549/ATCC	-5.62	> -4.00
HOP-62	-5.38	-4.33
HOP-92	-6.55	-5.29
NCI-H226	-5.29	> -4.00
NCI-H23	-5.67	> -4.00
NCI-H322M	-5.30	-4.31
NCI-H460	-5.47	-4.06
NCI-H522	-6.24	-5.25
**Colon Cancer**		
COLO 205	-5.79	-5.26
HCC-2998	-5.67	-5.17
HCT-116	-5.89	-5.20
HCT-15	-6.10	-4.50
HT29	-5.67	> -4.00
KM12	-5.47	> -4.00
SW-620	-6.02	> -4.00
**CNS Cancer**		
SF-268	-5.72	-4.81
SF-295	-5.35	-4.28
SF-539	-5.86	-5.26
SNB-19	-5.20	-4.33
SNB-75	-5.96	-5.26
U251	-5.66	-4.66
**Melanoma**		
LOX IMVI	-5.76	-5.22
MALME-3M	-6.03	-5.09
M14	-5.75	-5.09
MDA-MB-435	-5.75	-4.32
SK-MEL-2	-5.64	-4.74
SK-MEL-28	-5.78	-5.21
SK-MEL-5	-5.80	-5.25
UACC-257	-5.87	-5.20
UACC-62	-5.86	-5.21
**Ovarian Cancer**		
IGROV1	-5.67	-5.01
OVCAR-3	-5.97	-5.23
OVCAR-4	-5.98	-5.11
OVCAR-5	-5.70	-5.12
OVCAR-8	-5.77	-4.26
NCI/ADR-RES	-5.54	> -4.00
SK-OV-3	-5.33	-4.38
**Renal Cancer**		
786-0	-5.78	-5.23
A498	-5.82	-5.07
ACHN	-5.95	-5.30
CAKI-1	-5.88	-5.16
RXF 393	-6.22	-5.35
SN12C	-5.71	-4.82
TK-10	-5.83	-5.24
UO-31	-5.88	-5.27
**Prostate Cancer**		
PC-3	-5.84	> -4.00
DU-145	-5.78	-5.09
**Breast Cancer**		
MCF7	-5.85	> -4.00
MDA-MB-231/ATCC	-5.84	-5.09
HS 578T	-5.56	> -4.00
BT-549	-5.97	-5.24
T-47D	-5.96	> -4.00
MDA-MB-468	-6.38	-5.19
MID	-5.8	-4.69
Delta	0.75	0.66
Range	1.35	1.35

GI50

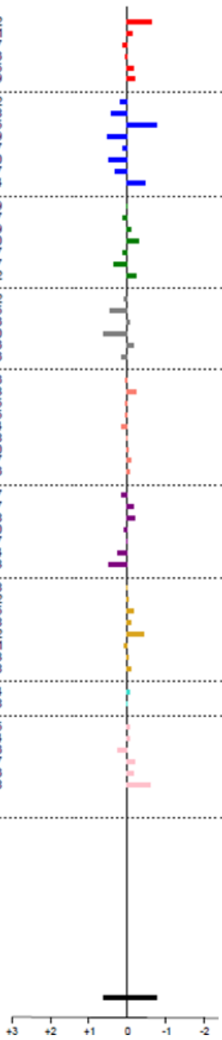

LC50

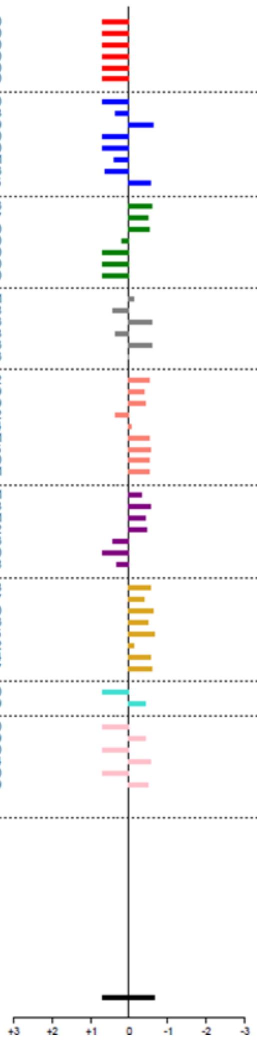

SI Table 1: IC_{50} determined by MTT assays. Mean concentration (± standard deviation) in µM required to reduce the viability of cells by 50% after a 72 h treatment relative to the control.

Compound	Parental sensitive models					Drug-resistant models				
	A2780	Hct116	RKO/ P53wt	KB-3-1	mean	A2780R	Hct116/ OxR	RKO/ p53ko	KB-C-1	mean
**1**	2.4±0.5	3.4±0.5	1.9±0.3	5.4±1.0	3	2.4±0.10	1.7±0.4	1.5±0.1	8.1±0.7	4
**13**	1.6±0.02	1.0±0.1	1.1±0.06	1.9±0.05	1	1.6±0.09	0.8±0.05	1.5±0.05	3.2±0.6	2
**15**	> 100	> 100	> 100	> 100	> 100	> 100	> 100	> 100	> 100	> 100
**16**	7.7±0.9	7.1±0.2	5.6±0.2	13.8±1.4	8	8.3±0.3	6.6±1.3	6.9±0.4	15.2±1.3	9
oxaliplatin		0.6±0.08	-	-		-	22.1±2.3	-	-	
cisplatin	1.8±0.09	-	2.0±0.3	-		>10	-	3.7±1.2	-	
doxorubicin	-	-	-	0.059±0.03		-	-		>250	

SI Table 2 : Information about the compounds displaying a COMPARE correlation coefficient (CCC) > 0.7 when comparing the 60 cancer cell line panel profile of **13** at the LC_{50} level to the whole NCI database

Compound	CCC	LC_{50} (NCI , µM)	Scientific information	References
Teroxirone (NSC 296934)	0.850	387	Anti-cancer effects against lung and liver cancers , NCI in vivo efficacy data	[1-4]
Caracemide (NSC 253272)	0.803	625	Inhibits bacterial ribonucleases , No effects in Phase II clinical trials	[5-8]
Acodazole (NSC 305884)	0.795	114	DNA intercalating agent , in vivo efficacy but cardiotoxic	[9]
Amsacrine (NSC 249992)	0.792	25	Marketed anti-cancer agent against acute leukemia , DNA interaclating agent and topo II inhibitory agent	[10,11]
Tamoxifen (NSC 180973)	0.788	26	Marketed hormonotherapeutic agent as oestrogen receptor modulator	[12]
Pyrazolo acridine (NSC 366140)	0.732	23.3	Topo I and topo II inhibitor , moderate anti-cancer effects in phase II clinical trial	[13]
Cytembena (NSC 104801)	0.706	802	DNA synthesis inhibition (Direct inhibitor of DNA replication complex) Phase II clinical trial showed regression in 20% of the uterine cervix cancers	[14-16]

SI Figure 5: Exemplary full gel 1 of decatenation assay. From left to right: no enzyme control, solvent control, 1 (5 µM), 1 (50 µM), 1 (500 µM) , 15 (5 µM), 15 (50 µM), 15 (500 µM), 13 (5 µM), 13 (50 µM), 13 (500 µM) , 16 (5 µM), 16 (50 µM), 16 (500 µM), positive control, solvent control. The cropped image provided in Fig. 5 shows the first 8 lanes from left to right as well as the positive control (second lane from the right).

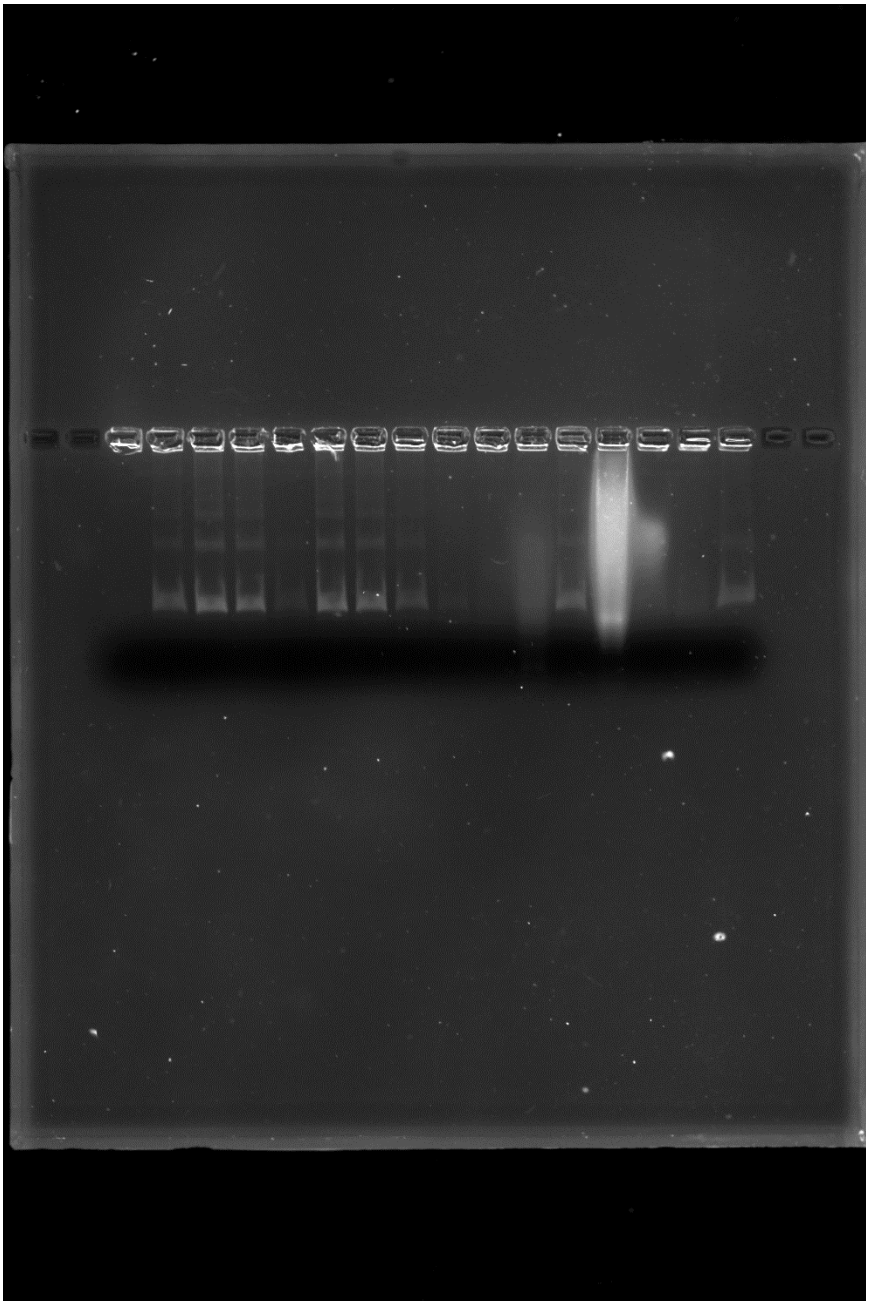

SI Figure 6: Exemplary full gel 2 of decatenation assay. From left to right: no enzyme control, positive control, solvent control, 13 (0.1 µM), 13 (0.5 µM), 13 (1 µM), 13 (5 µM), 13 (50 µM), 16 (0.1 µM), 16 (0.5 µM), 16 (1 µM), 16 (5 µM), 16 (50 µM), solvent control, 13 (0.1 µM), 13 (1 µM), 16 (0.1 µM), 16 (1 µM). The cropped image provided in Fig. 5 shows the lane number 2 to lane number 13 from left to right.

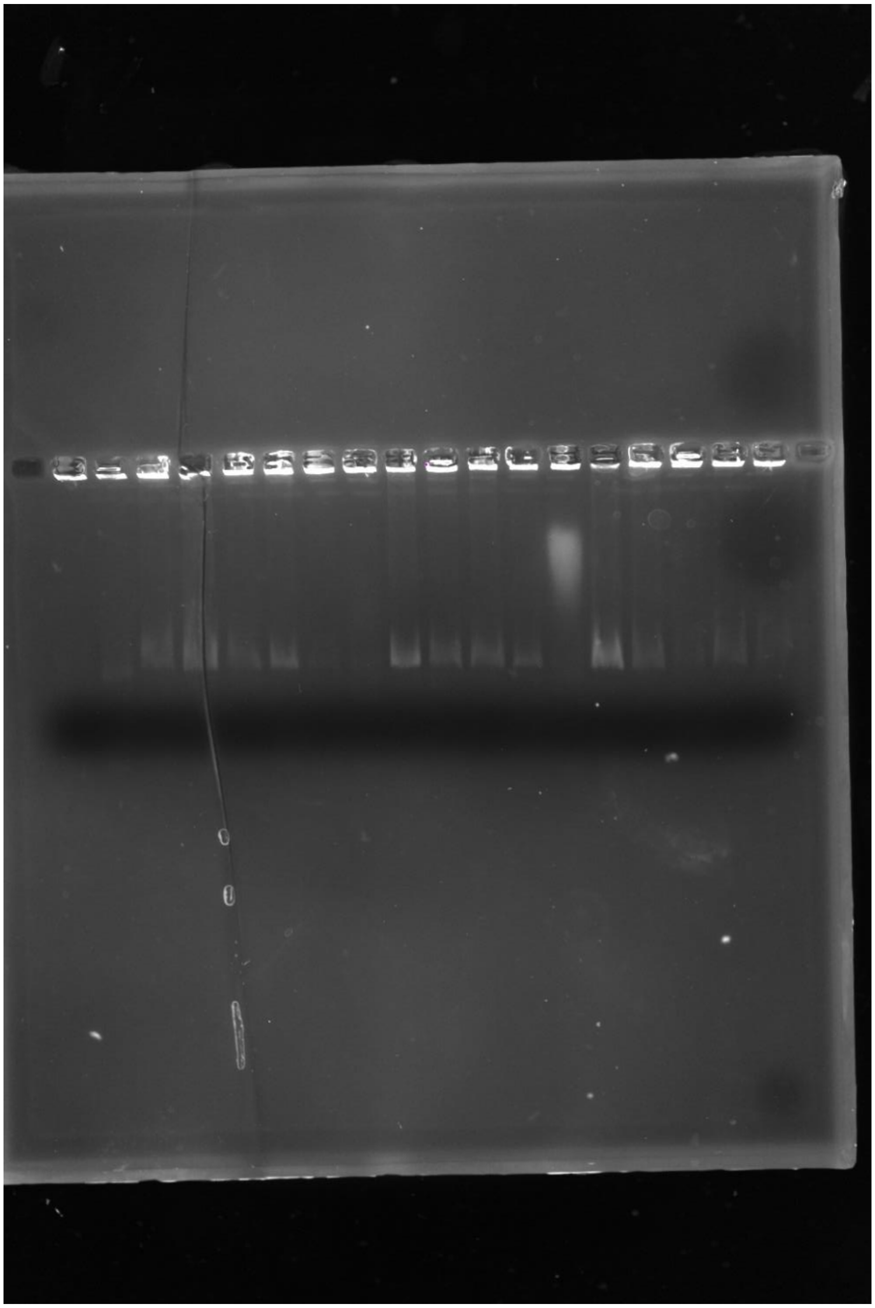

^{1}H NMR and ^{13}C NMR spectra of compound **3**

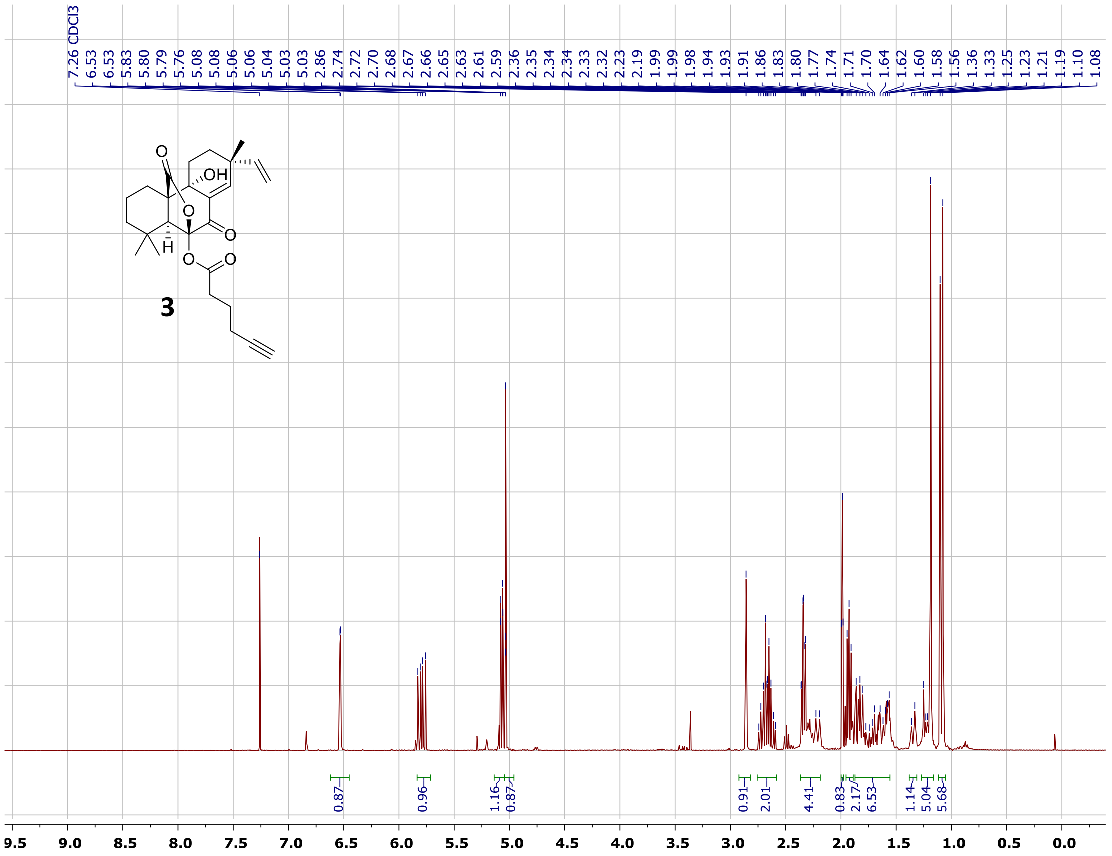

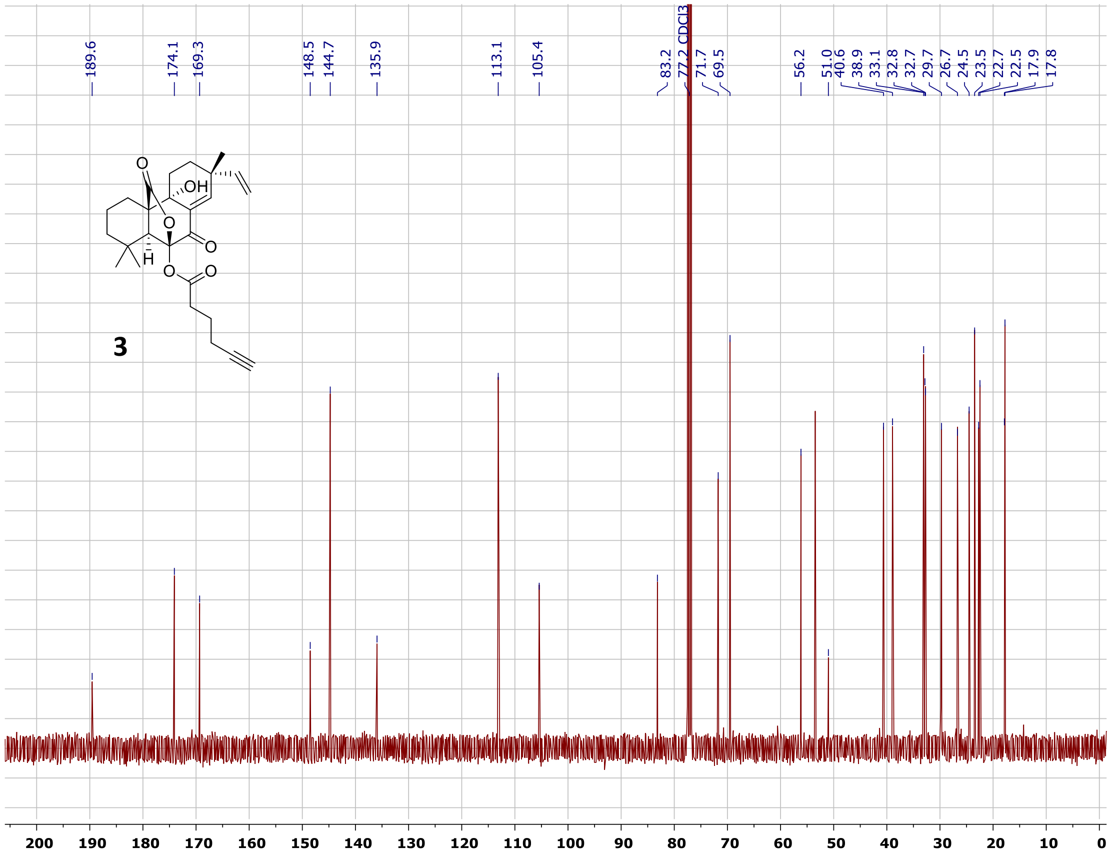

^{1}H NMR spectrum of compound **4**

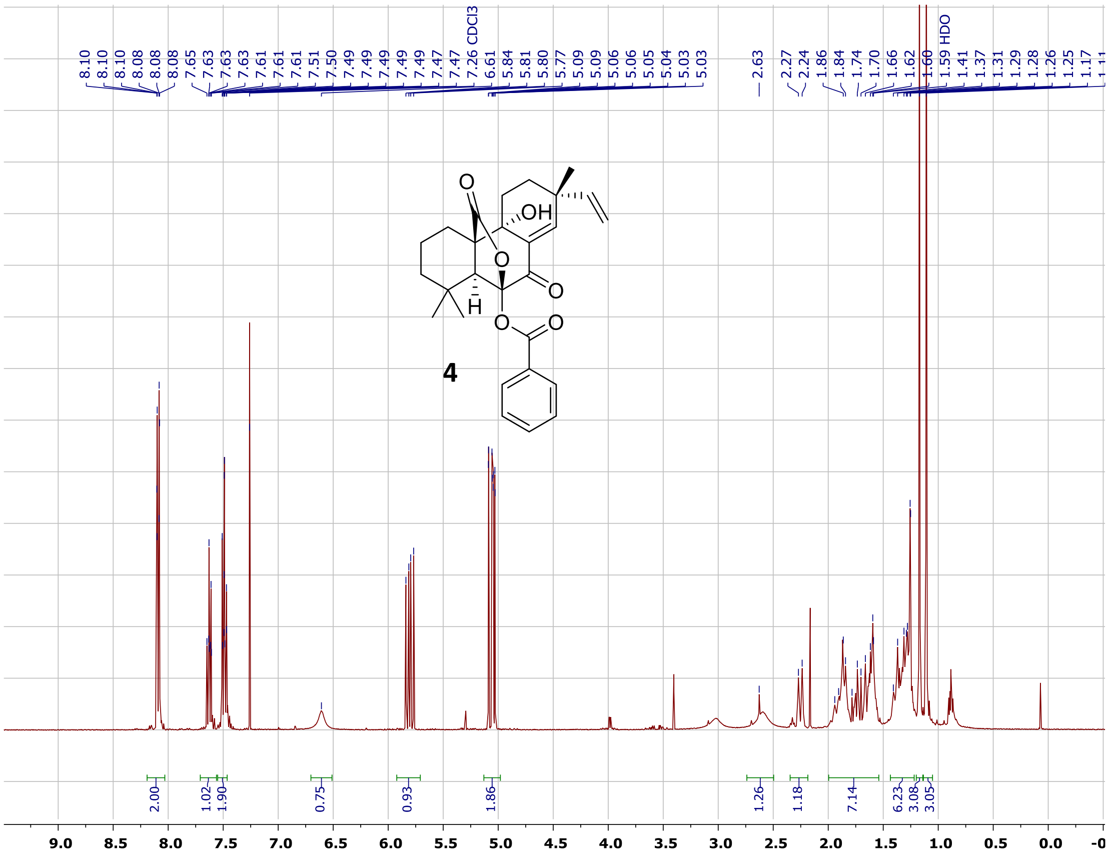

^{1}H NMR and ^{13}C NMR spectra of compound **5**

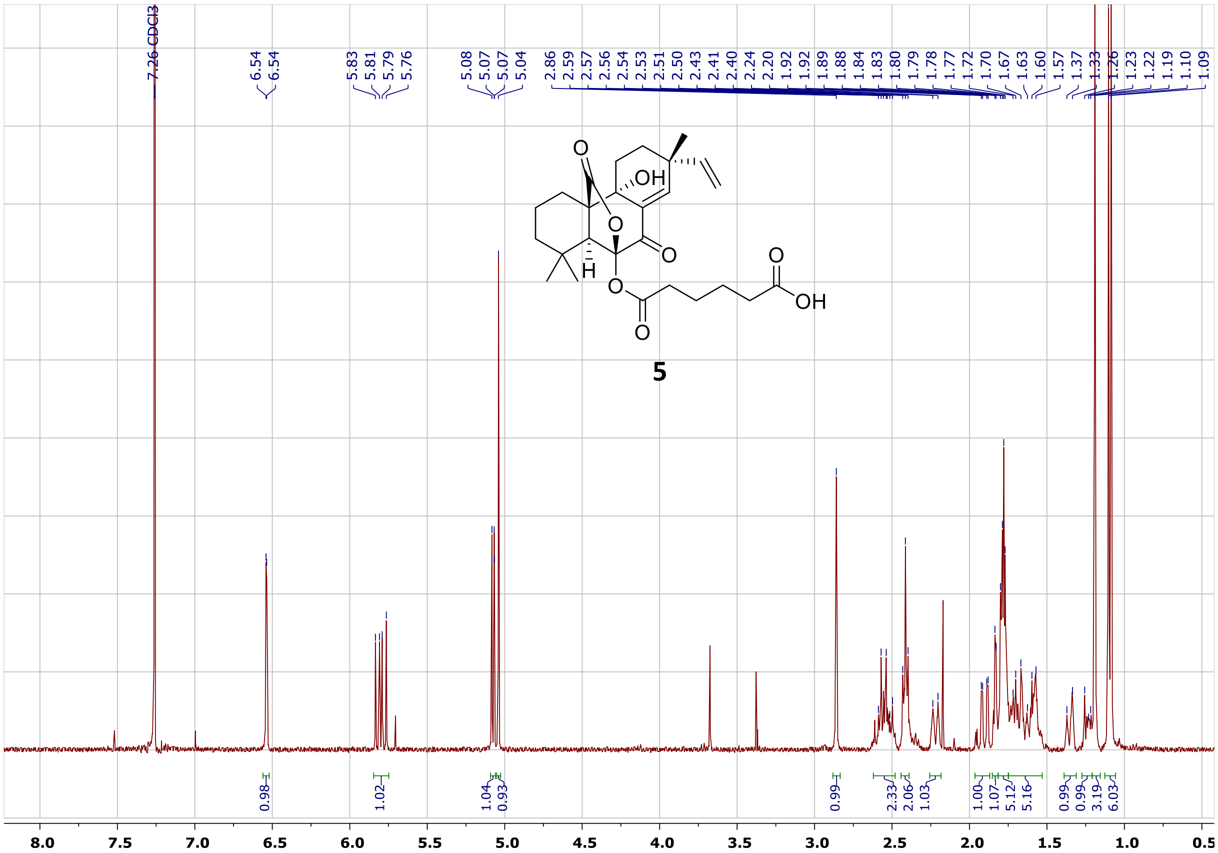

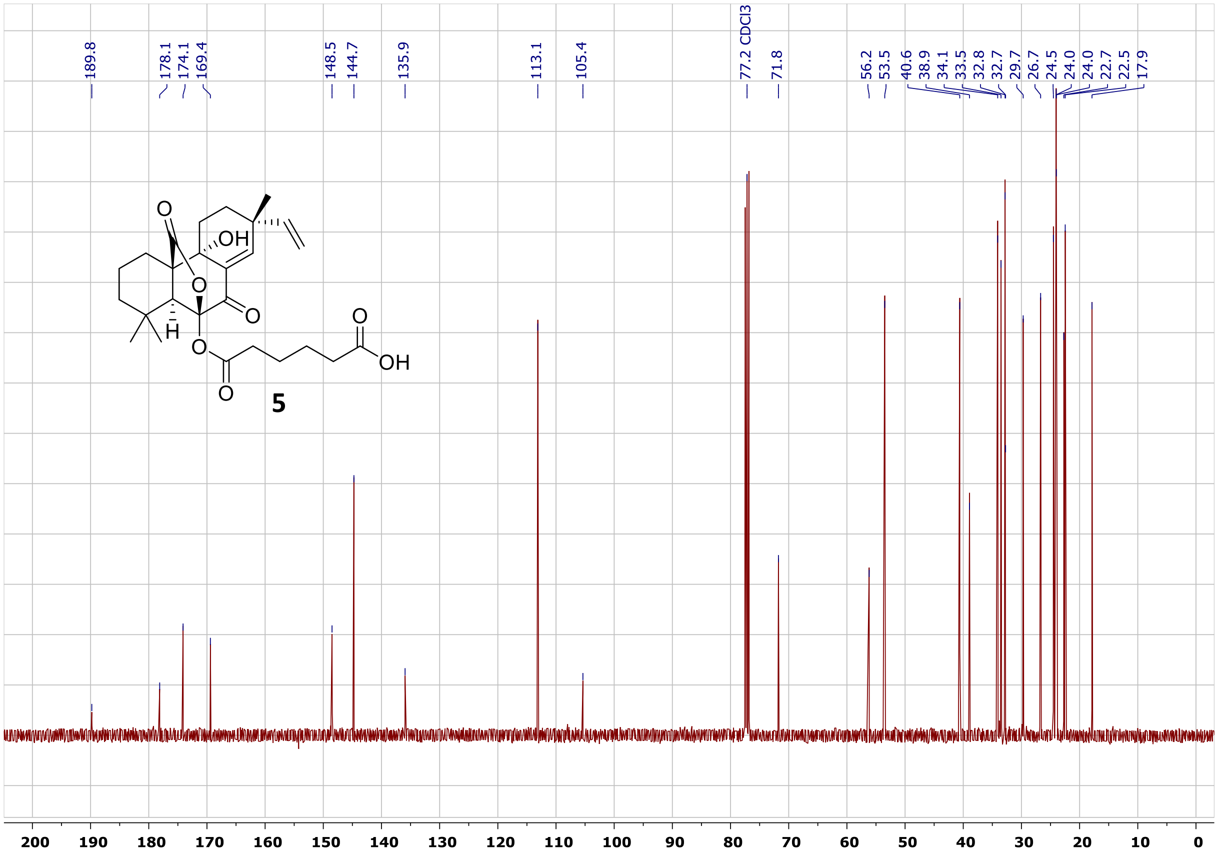

^{1}H NMR and spectrum of compound **6**

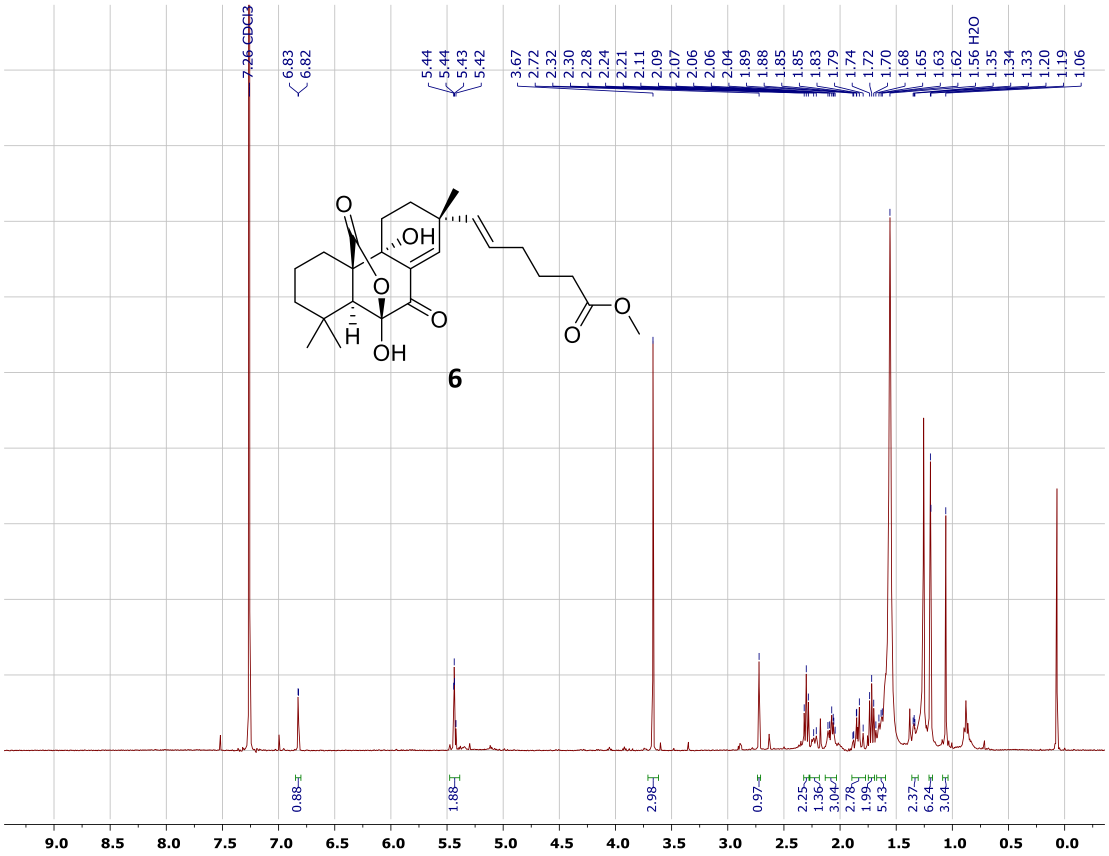

^{1}H NMR and spectrum of compound **7**

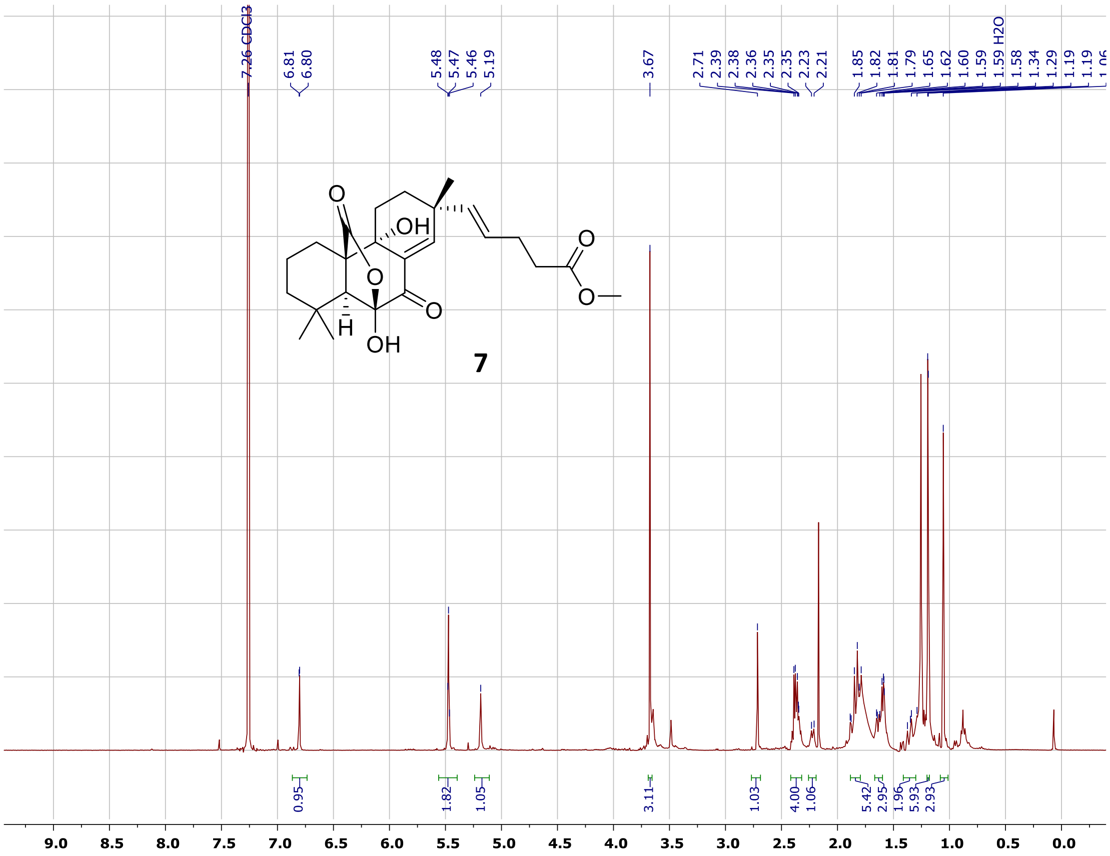

^{1}H NMR and ^{13}C NMR spectra of compound **8**

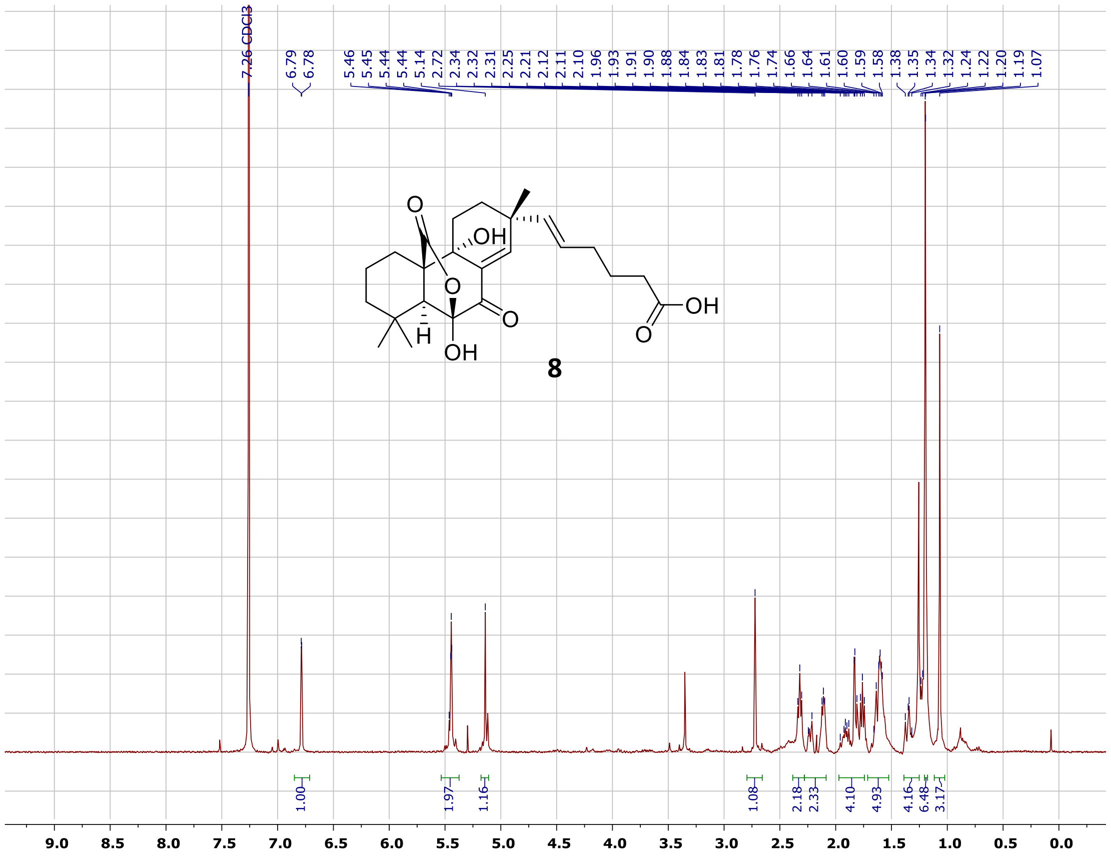

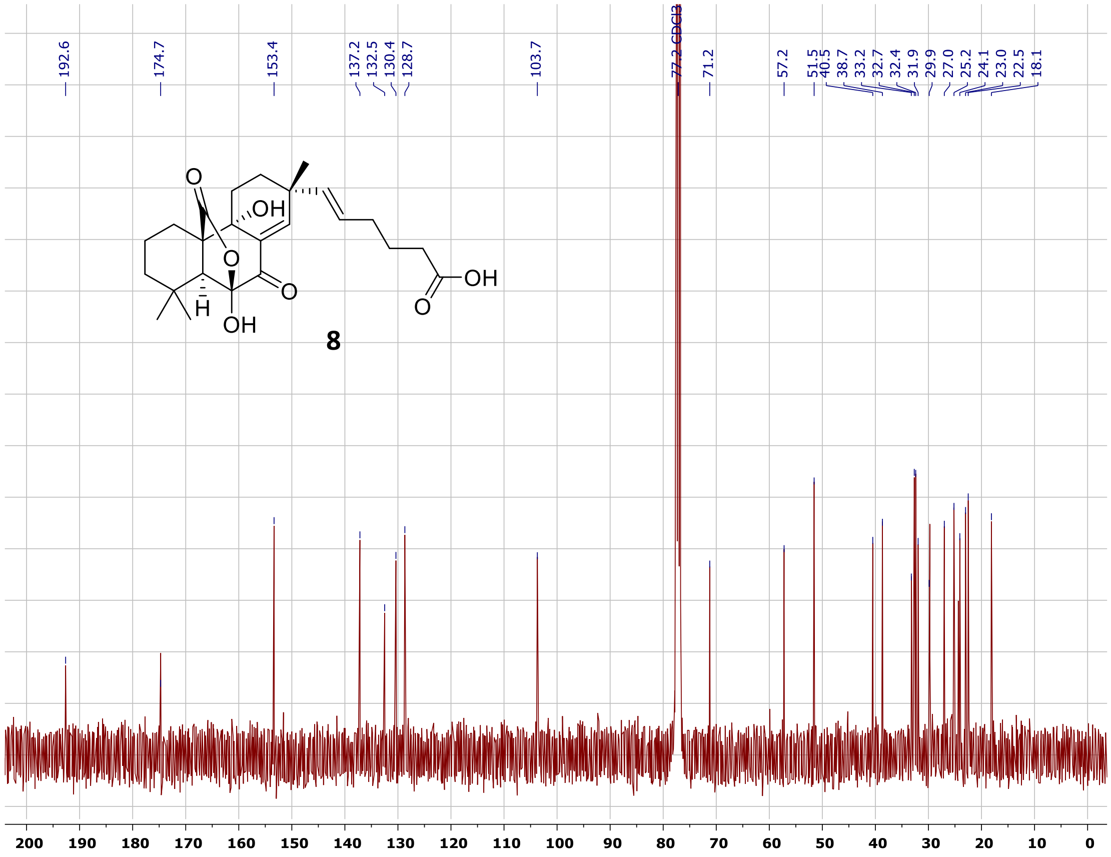

^{1}H NMR and ^{13}C NMR spectra of compound **9**

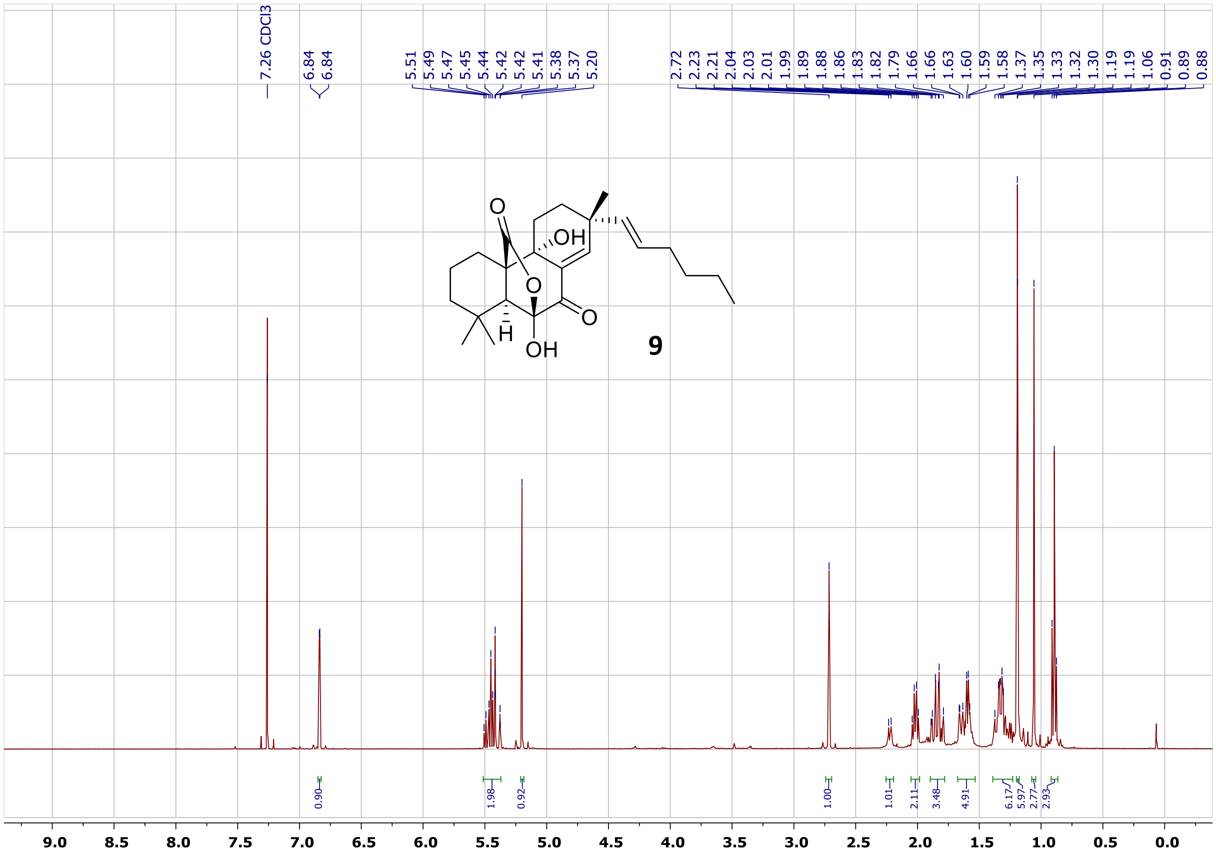

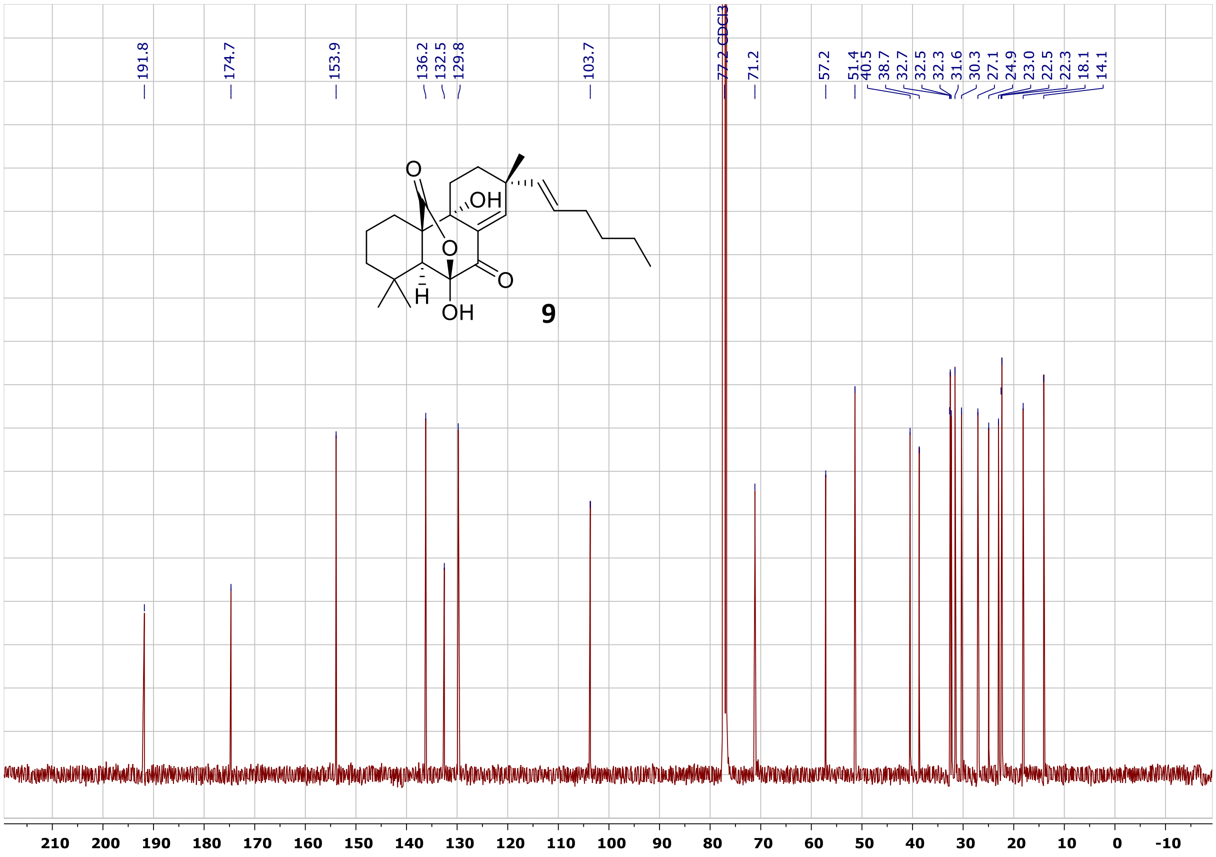

^{1}H NMR and spectrum of compound **10**

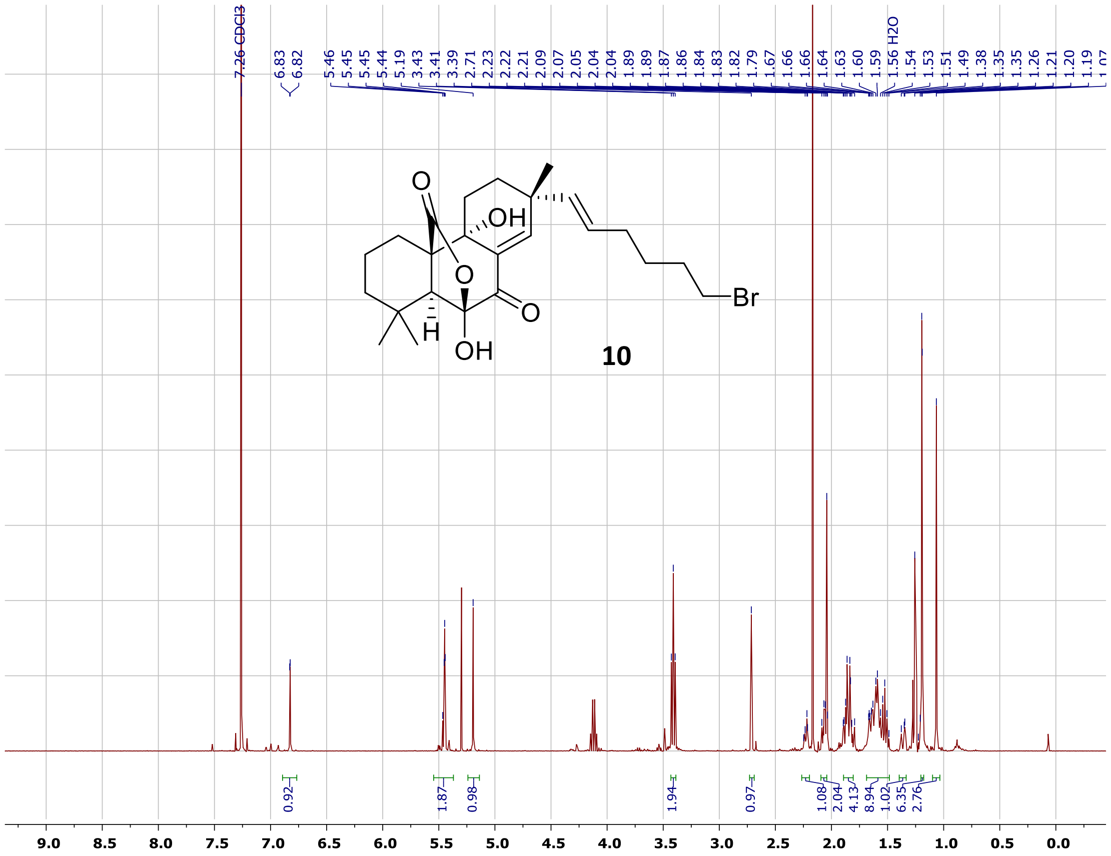

^{1}H NMR and spectrum of compound **11**

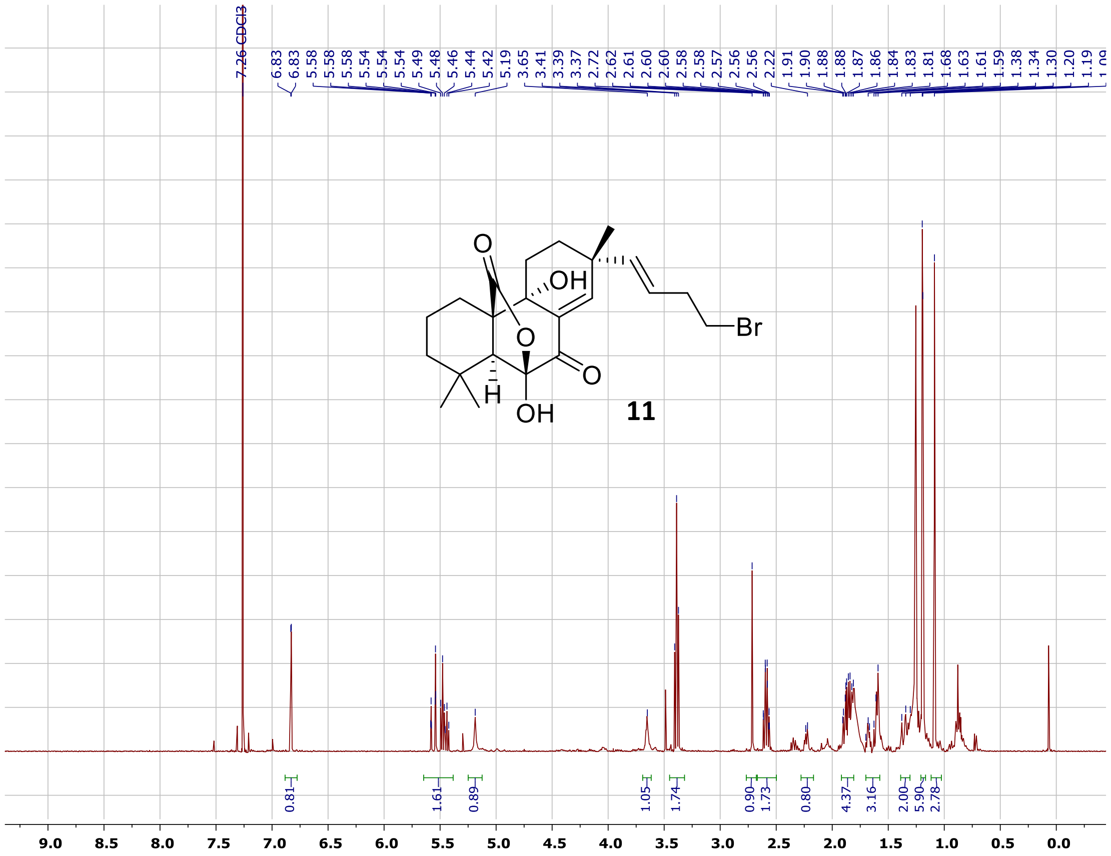

^{1}H NMR and spectrum of compound **12**

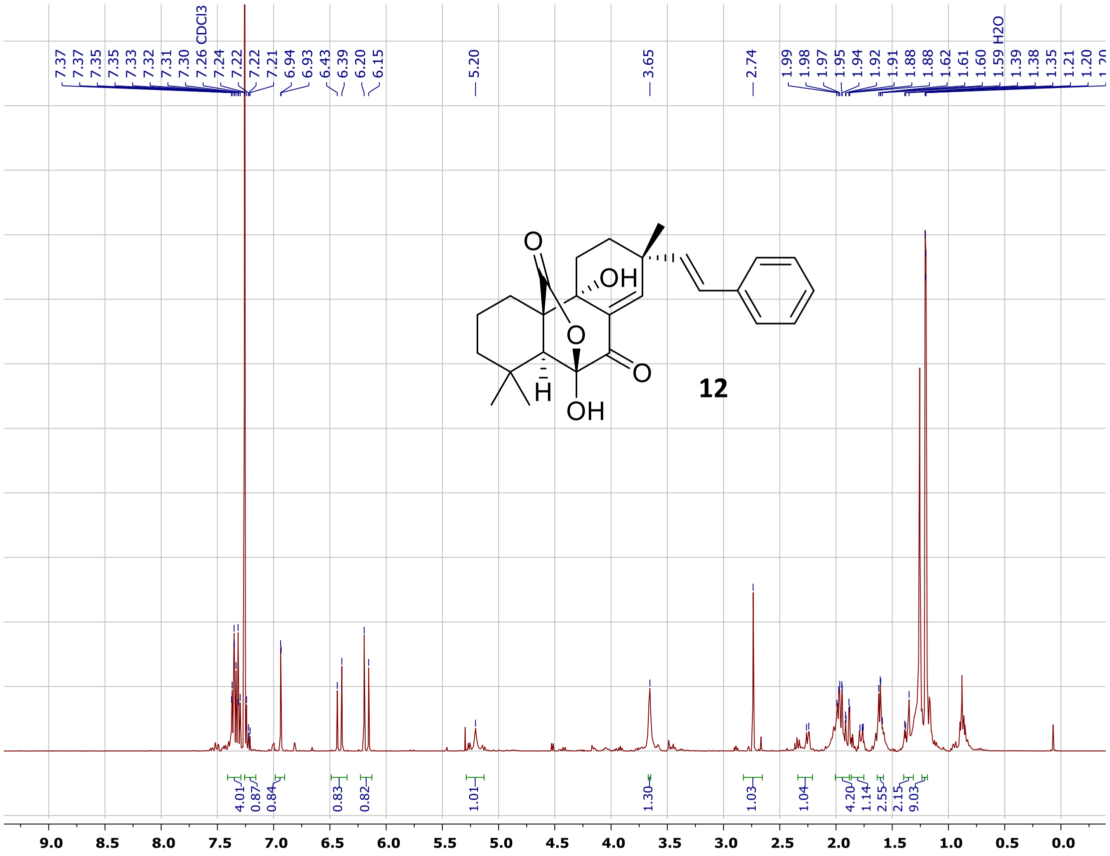

^{1}H NMR and ^{13}C NMR spectra of compound **13**

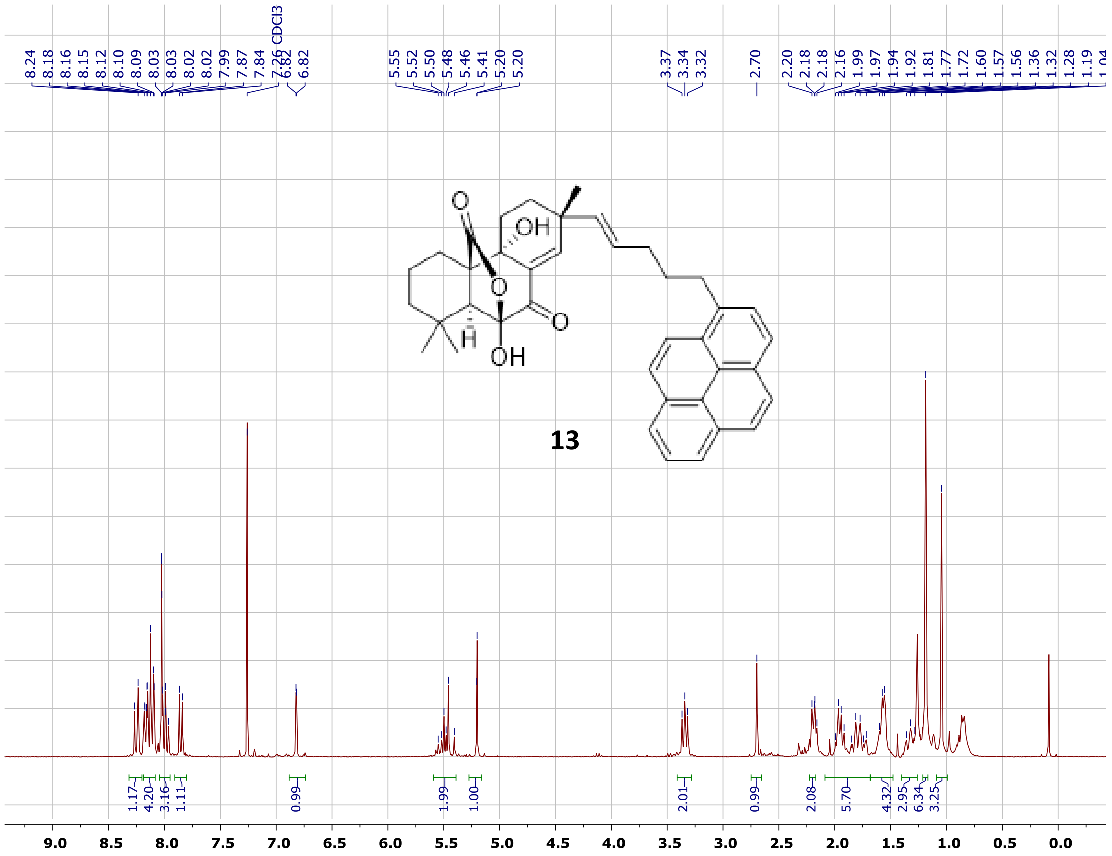

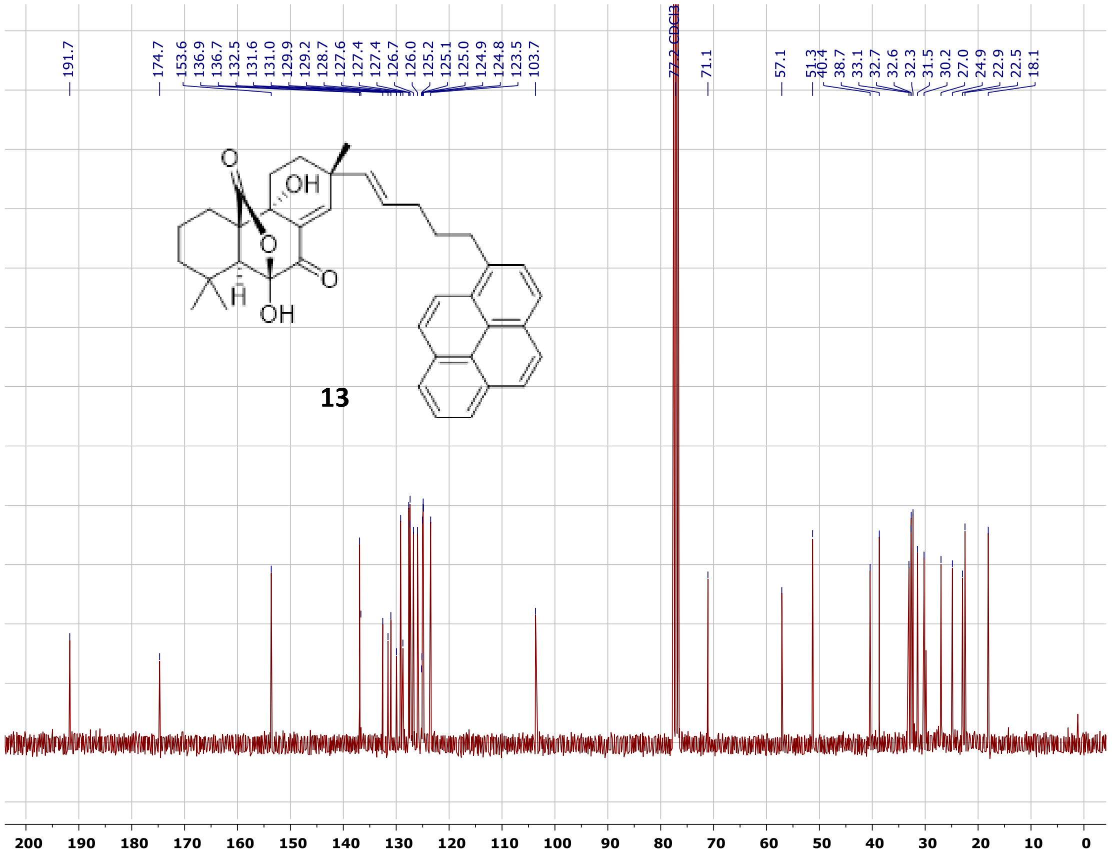

^{1}H NMR and spectrum of compound **16**

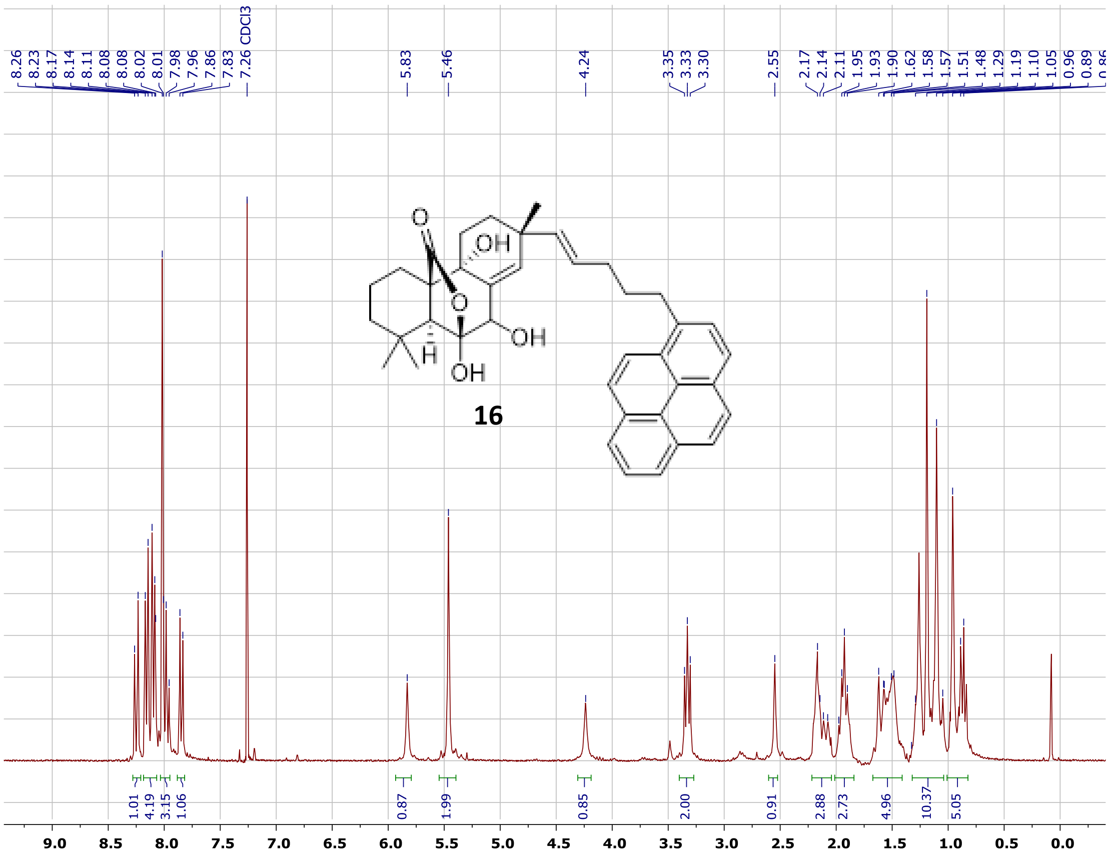

^{1}H NMR and ^{13}C NMR spectra of compound **18**

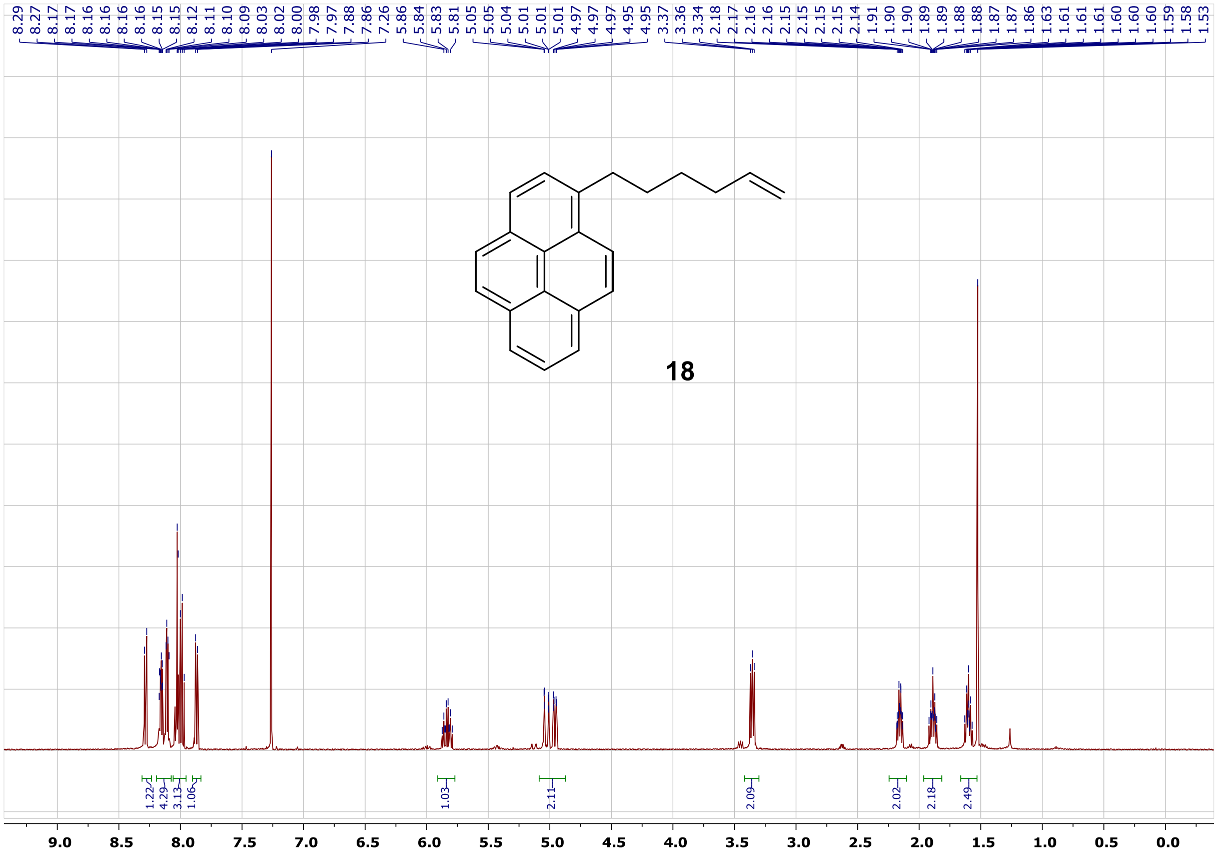

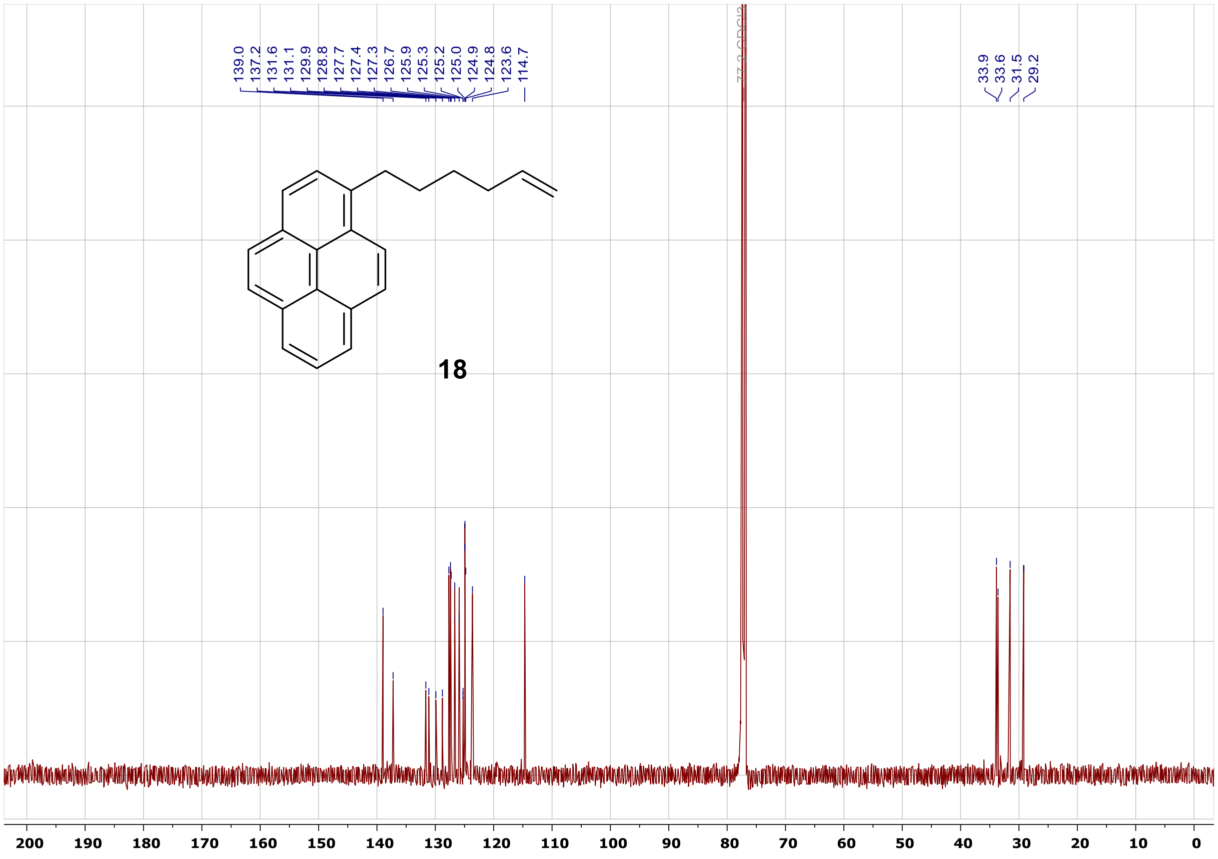

^{1}H NMR and ^{13}C NMR spectra of compound **19**

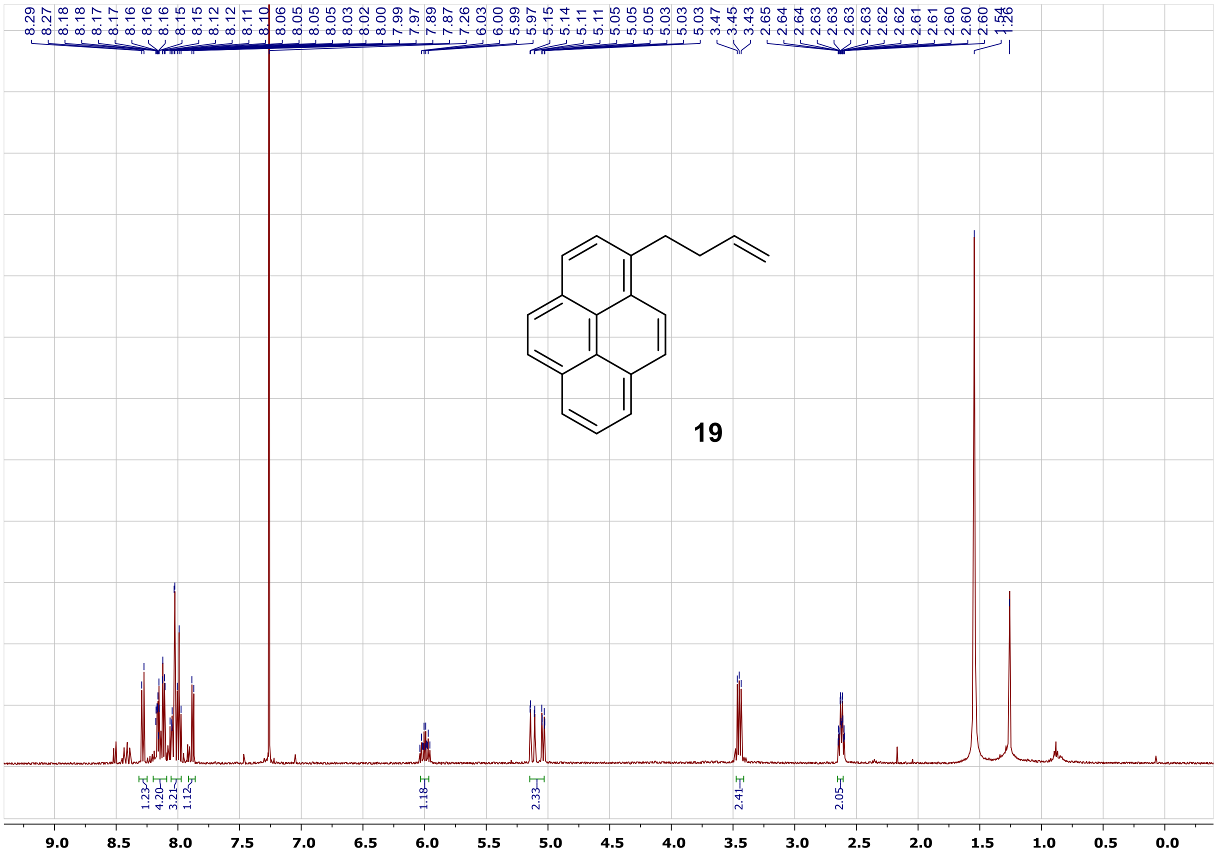

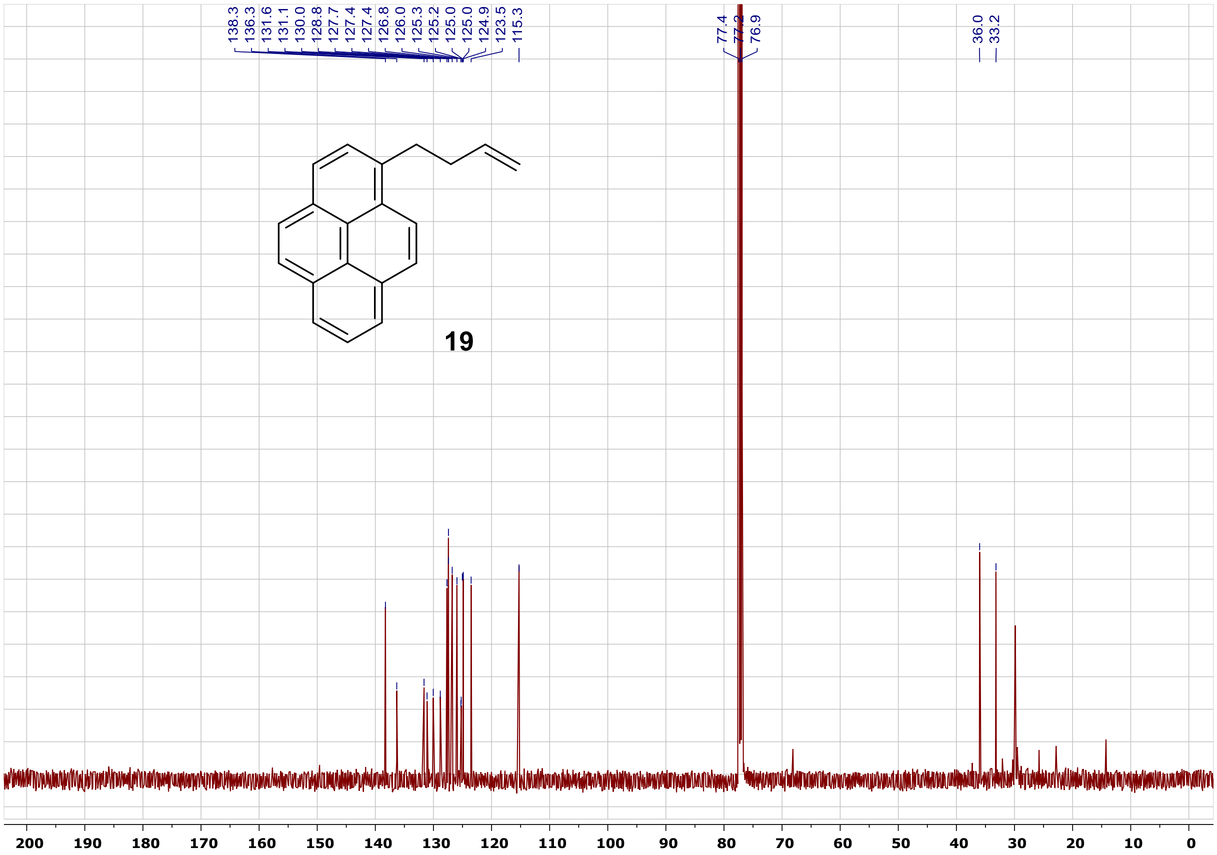

^{1}H NMR and ^{13}C NMR spectra of compound **23**

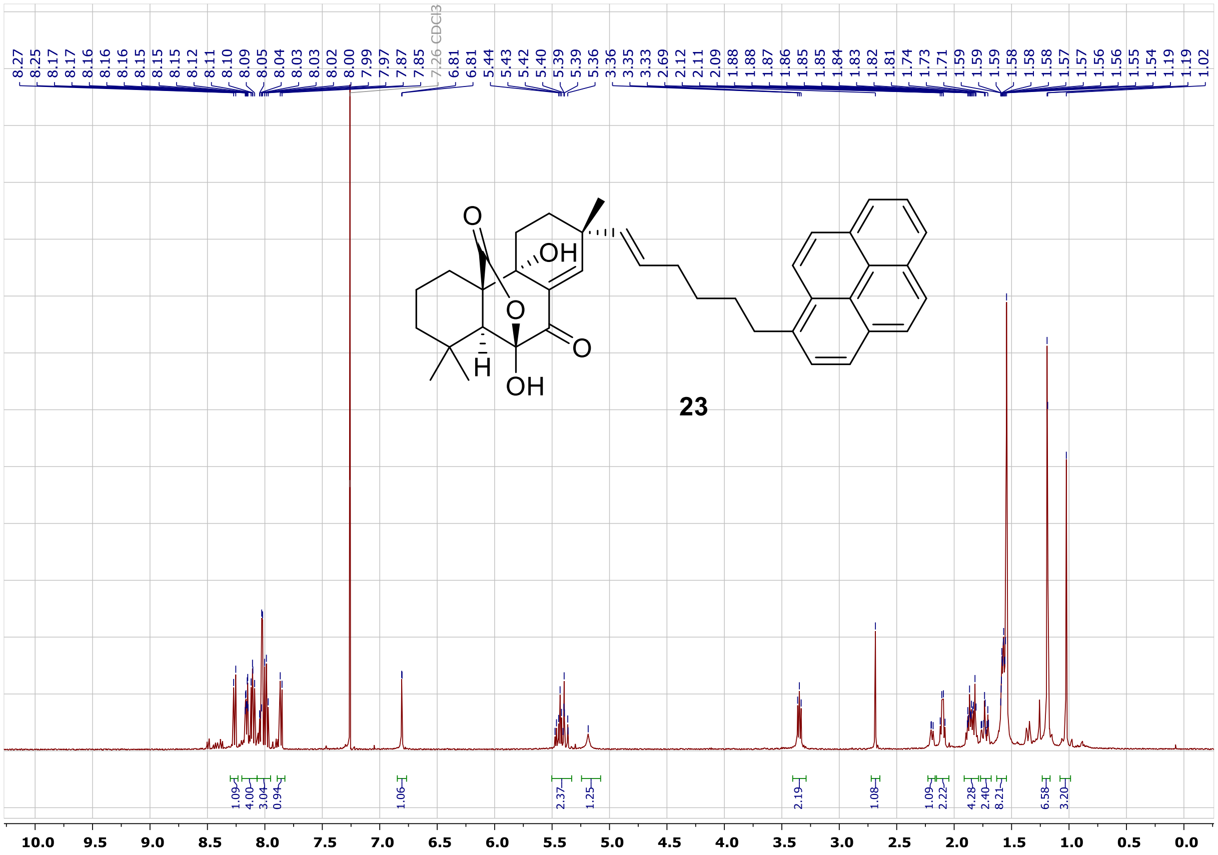

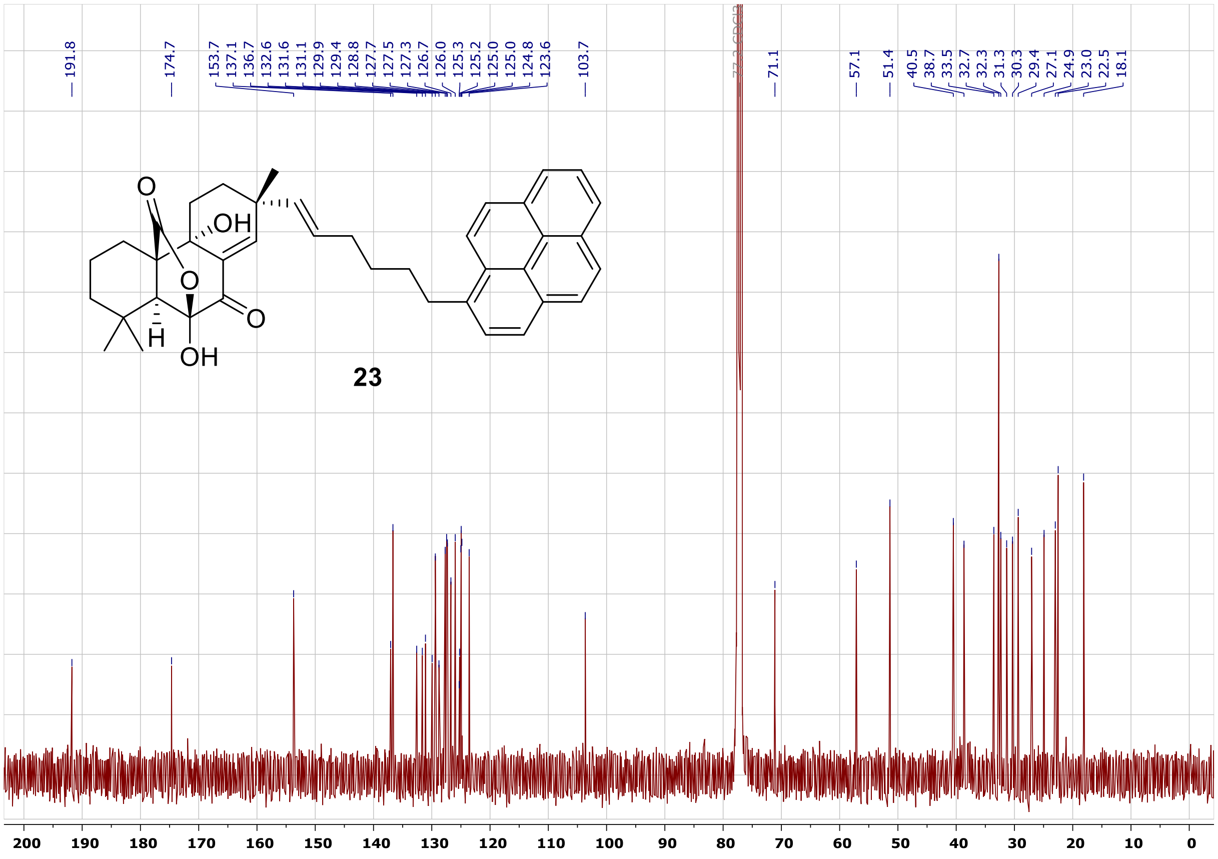

^{1}H NMR and spectrum of compound **24**

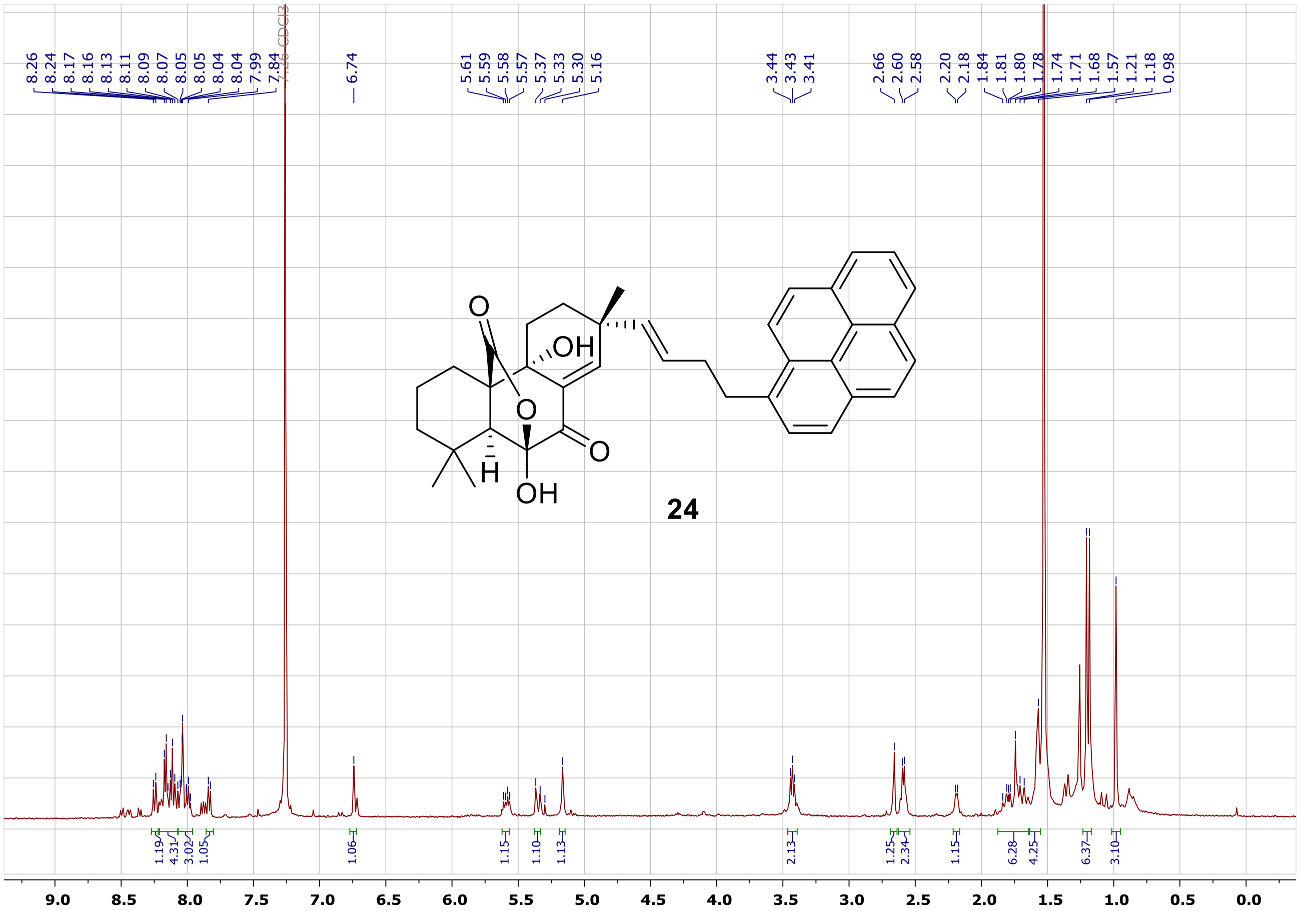

^{1}H NMR and spectrum of compound **25**

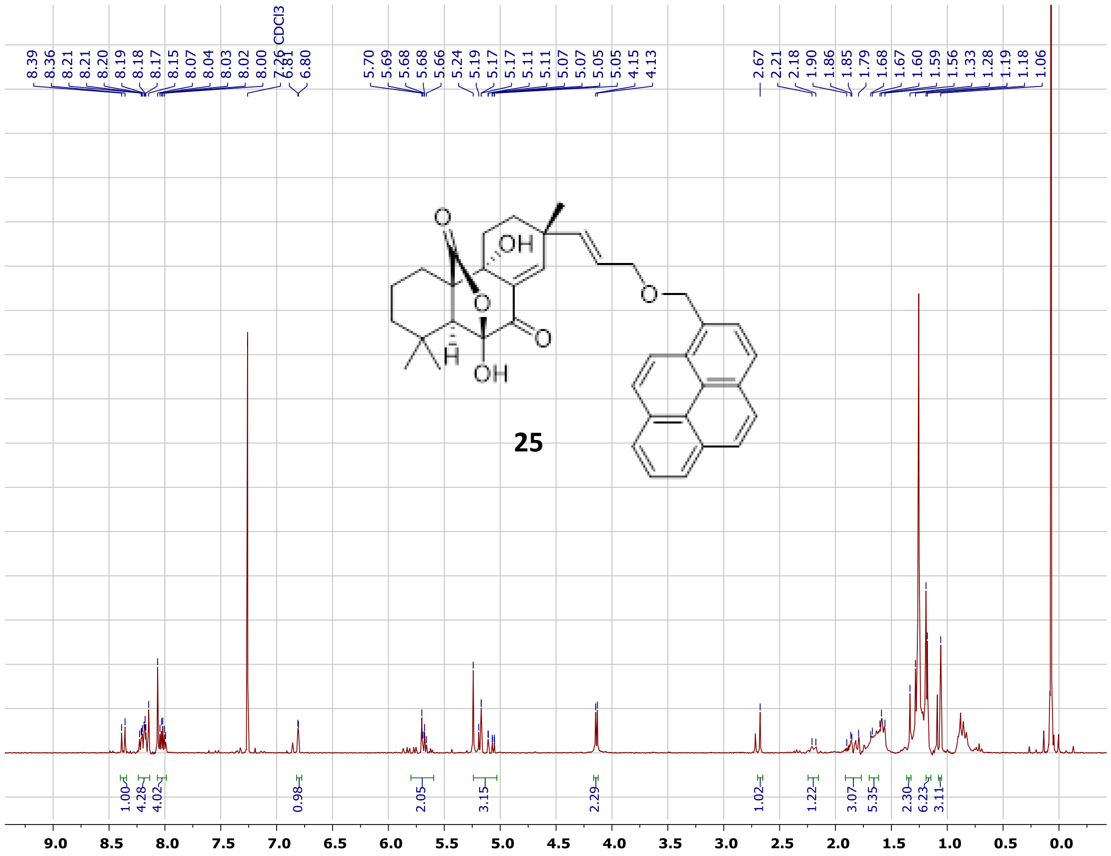

^{1}H NMR and spectrum of compound **26**

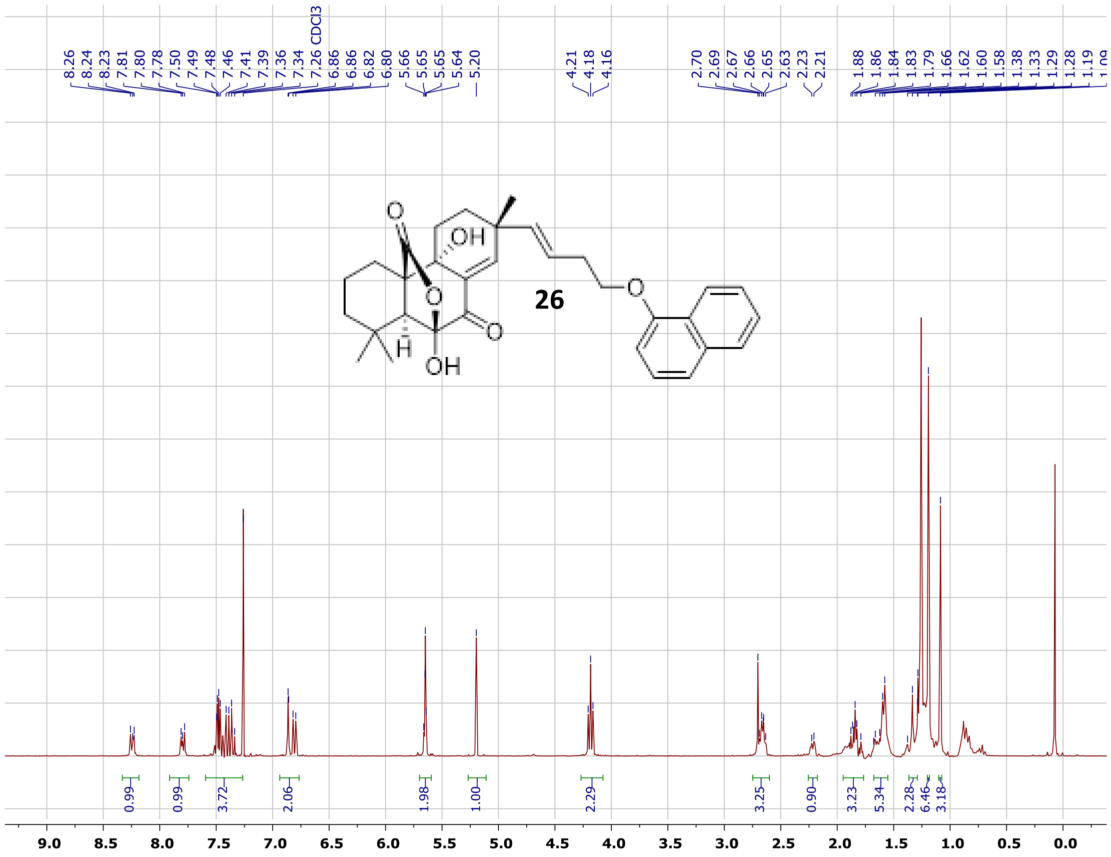

^{1}H NMR and ^{13}C NMR spectra of compound **27**

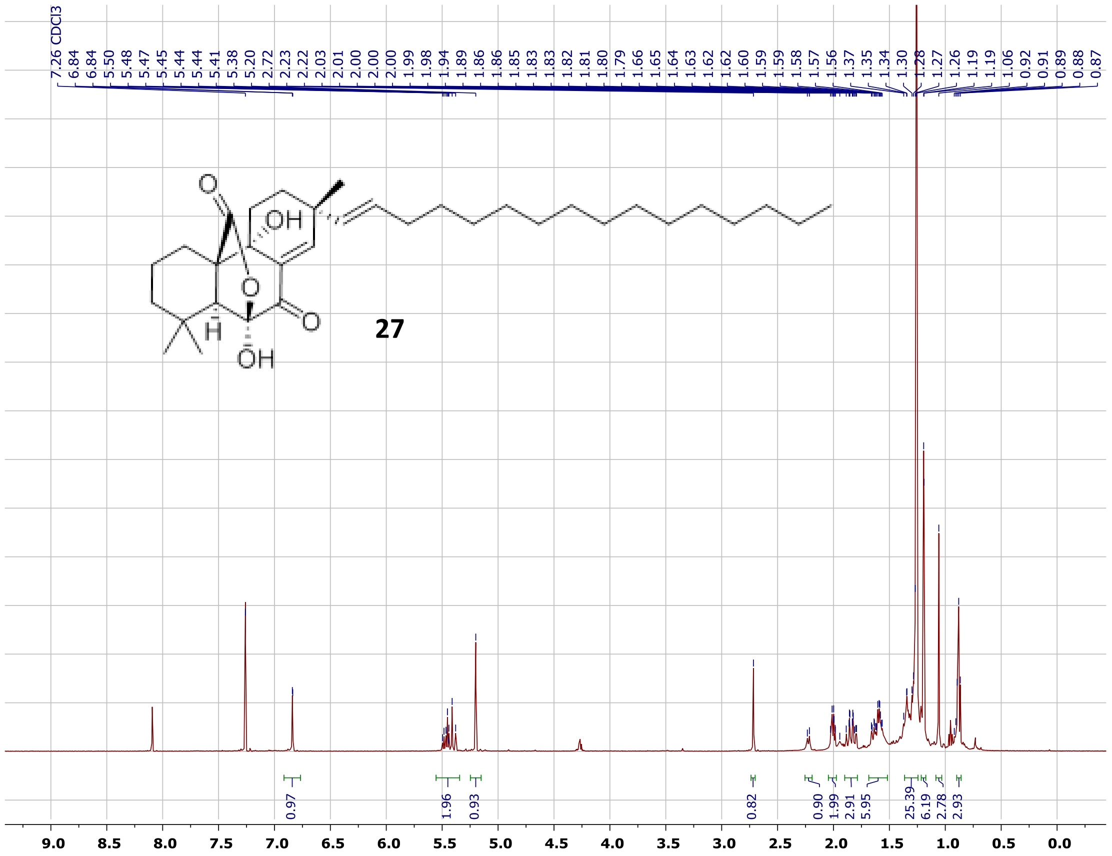

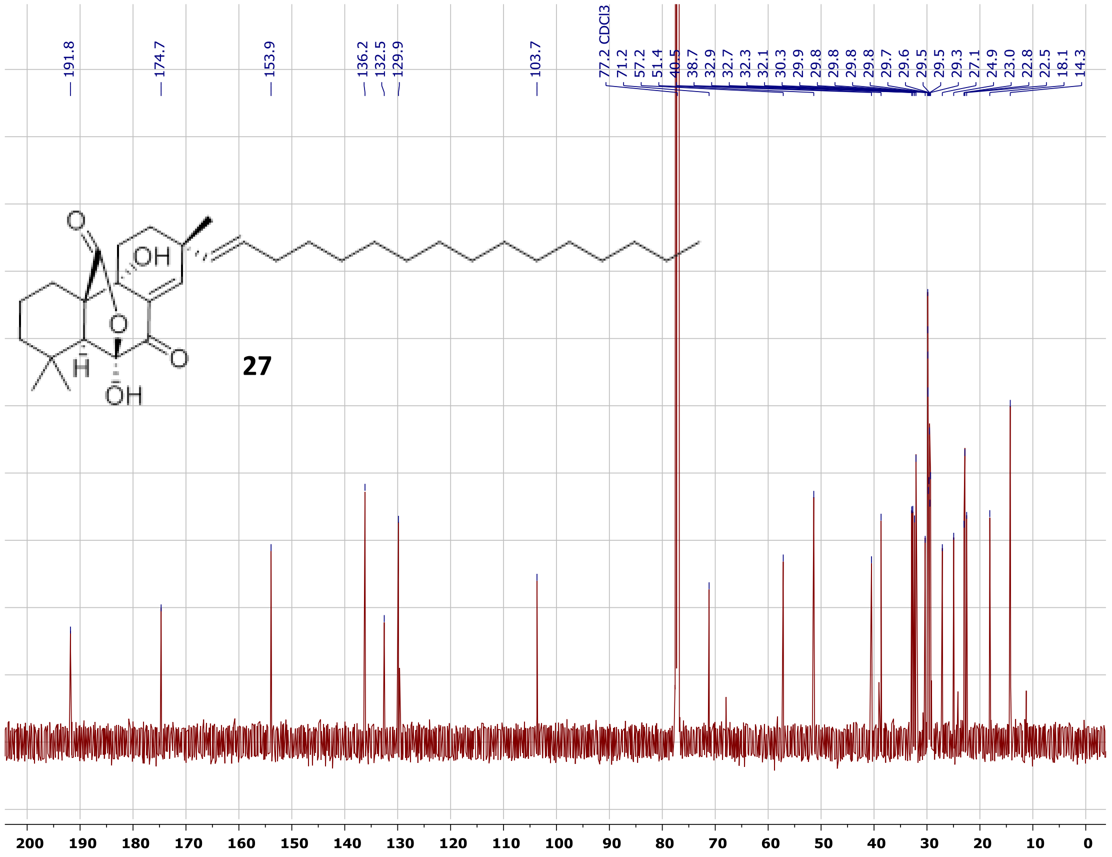

References

1. Dong, N., Du, Y., Zheng, Y., Zhang, H., Lv, H. & Yan, Z. Research progress on tamoxifen and its analogs associated with nuclear receptors. *Future Med Chem* **14**. (2023)

2. Baguley, B.C., Drummond, C.J., Chen, Y.Y. & Finlay, G.J.. DNA-Binding Anticancer Drugs: One Target, Two Actions. *Molecules* **26**,552. (2021)

3. Ni, Y.L., Hsieh, C.H., Wang, J.P. & Fang, K. Teroxirone motivates apoptotic death in tumorspheres of human lung cancer cells. *Chem Biol Interact*. **1**, 137-143. (2018).

4. Kim, S.H., Wang, W.H., Wang, J.P., Hsieh, C.H. & Fang, K. Teroxirone suppresses growth and motility of human hepatocellular carcinoma cells. *Biomed Pharmacother* **99**, 997-1008. (2018).

5. Wang, J.P. et al. Reactive oxygen species-driven mitochondrial injury induces apoptosis by teroxirone in human non-small cell lung cancer cells. *Oncol Lett* **14**, 3503-3509. (2017).

6. Wang, J.P. et al. Teroxirone inhibited growth of human non-small cell lung cancer cells by activating p53. *Toxicol Appl Pharmacol* **273**,110-20. (2013).

7. Ramaswamy, B., Mrozek, E., Kuebler, J.P., Bekaii-Saab, T. & Kraut, E.H. Phase II trial of pyrazoloacridine (NSC#366140) in patients with metastatic breast cancer. *Invest New Drugs* **29**, 347-51. (2011).

8. Witte, R.S. et al. A phase II trial of homoharringtonine and caracemide in the treatment of patients with advanced large bowel cancer. *Invest New Drugs* **17**,173-177. (1999).

9. Witte, R.S., Hsieh, P., Elson, P., Oken, M.M. & Trump, D.L. A phase II trial of amonafide, caracemide, and homoharringtonine in the treatment of patients with advanced renal cell cancer. *Invest New Drugs* **14**, 409-413. (1996).

10. Lad, T. et al. Phase II trial of caracemide (NSC 253272) in advanced unresectable non-small cell bronchogenic carcinoma. An Illinois Cancer Council study. *Invest New Drugs* **10**, 27-28. (1992).

11. Trump, D.L. et al. Phase I clinical trial and pharmacokinetic evaluation of acodazole (NSC 305884), an imidazoquinoline derivative with electrophysiological effects on the heart. *Cancer Res* **47**, 3895-3900. (1987).

12. Cassileth, P.A. & Gale, R.P. Amsacrine: a review. *Leuk Res* **10**, 1257-1265. (1986).

13. Moore, E.C. & Loo, T.L. Inhibition of ribonucleotide reductase by caracemide. *Cancer Treat Rep* **68**, 1293-124. (1984).

14. Clinical screening group of E.O.R.T.C. A phase II clinical trial of cytembena. *Biomedicine* ***26***, 392-395. (1977).

15. Berger, N.A. & Weber, G. Description of a permeable eukaryotic cell system to study agents affecting DNA synthesis: demonstration that cytembena is a direct inhibitor of replicative DNA synthesis. *J Natl Cancer Inst* **58**,1167-1169. (1977).

16. Falkson, H.C. & Falkson, G. Phase II trial of cytembena in patients with advanced ovarian and breast cancer. *Cancer Treat Rep* **60**, 1655-168. (1976).
